# Supplementary figures and images for: Genome-wide analysis reveals no evidence of trans chromosomal regulation of mammalian immune development
Source: PLoS Genet. 2018 Jun 8;14(6):e1007431. doi: 10.1371/journal.pgen.1007431 (PMC6010296; doi:10.1371/journal.pgen.1007431)

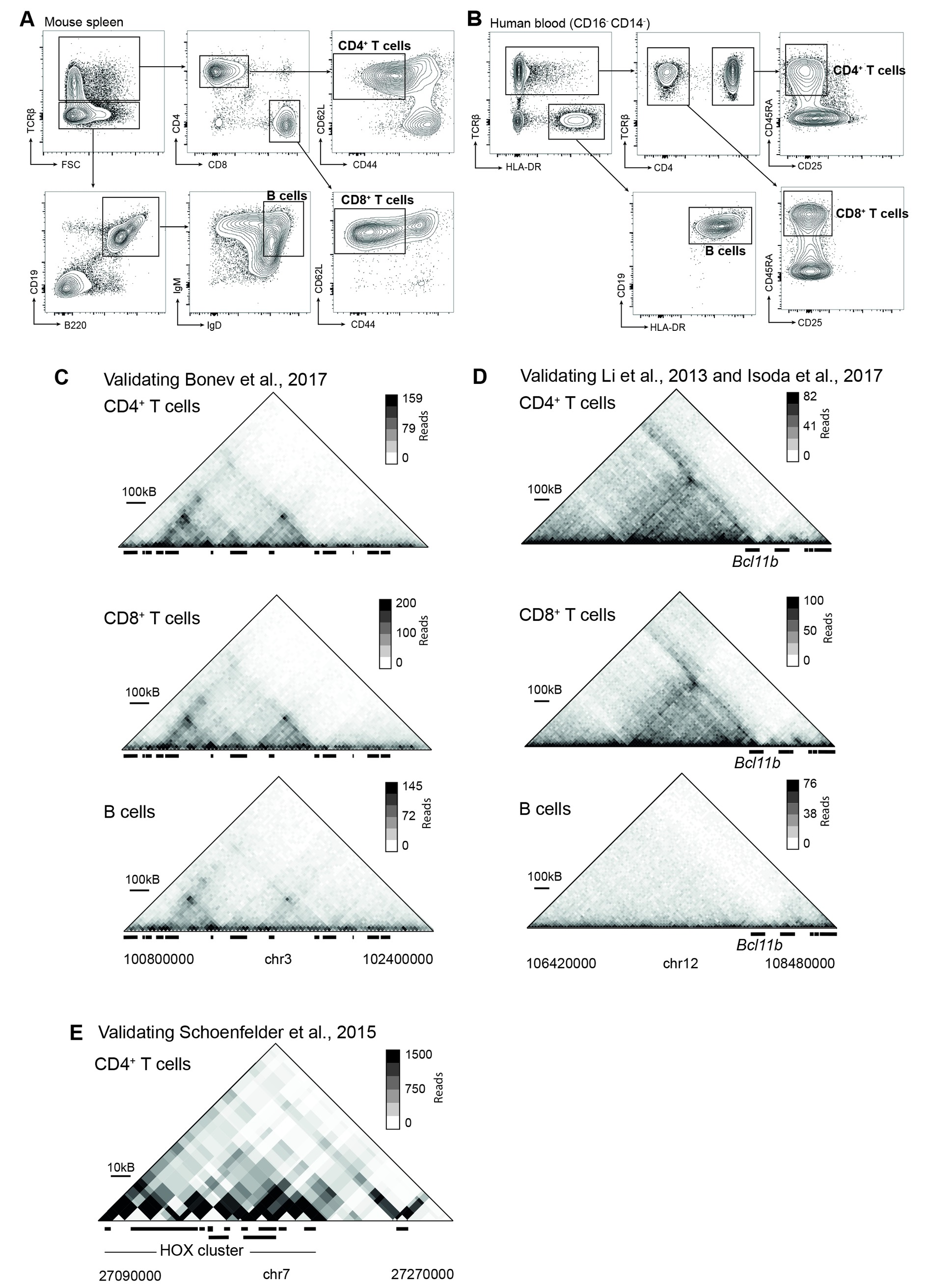

Supplement: S1 Fig — (A) Flow cytometry of homogenised C57BL/6 Pep3b mouse spleen stained with antibodies against TCRβ, CD4, CD8, CD62L, CD44, CD19, B220, IgD and IgM. CD4+ T cells were isolated as TCRβ+ CD4+ CD8- CD62L+ CD44-. CD8+ T cells were isolated as TCRβ+ CD4- CD8+ CD62L+ CD44-. B cells were isolated as TCRβ- CD19+ B220+ IgM+ IgD+. (B) Flow cytometry of human peripheral blood stained with antibodies against TCRβ, HLA-DR, CD4, CD45RA, CD25, and CD19. CD4+ T cells were isolated as TCRβ+ CD4+ CD45RA- CD25+. CD8+ T cells were isolated as TCRβ+ CD4- CD45RA- CD25+. B cells were isolated as TCRβ- HLA-DR+ CD19+. (C) HiC contact matrices of mouse immune cells confirming interactions previously reported during mouse neuronal development on mouse chromosome 3 (D) HiC contact matrices of mouse immune cells confirming T cell specific interactions previously reported on mouse chromosome 12 (E) Promoter capture HiC contact matrices in human CD4+ T cells confirming interactions previously reported in the mouse Hox cluster. (TIF) [file pgen.1007431.s001.tif]

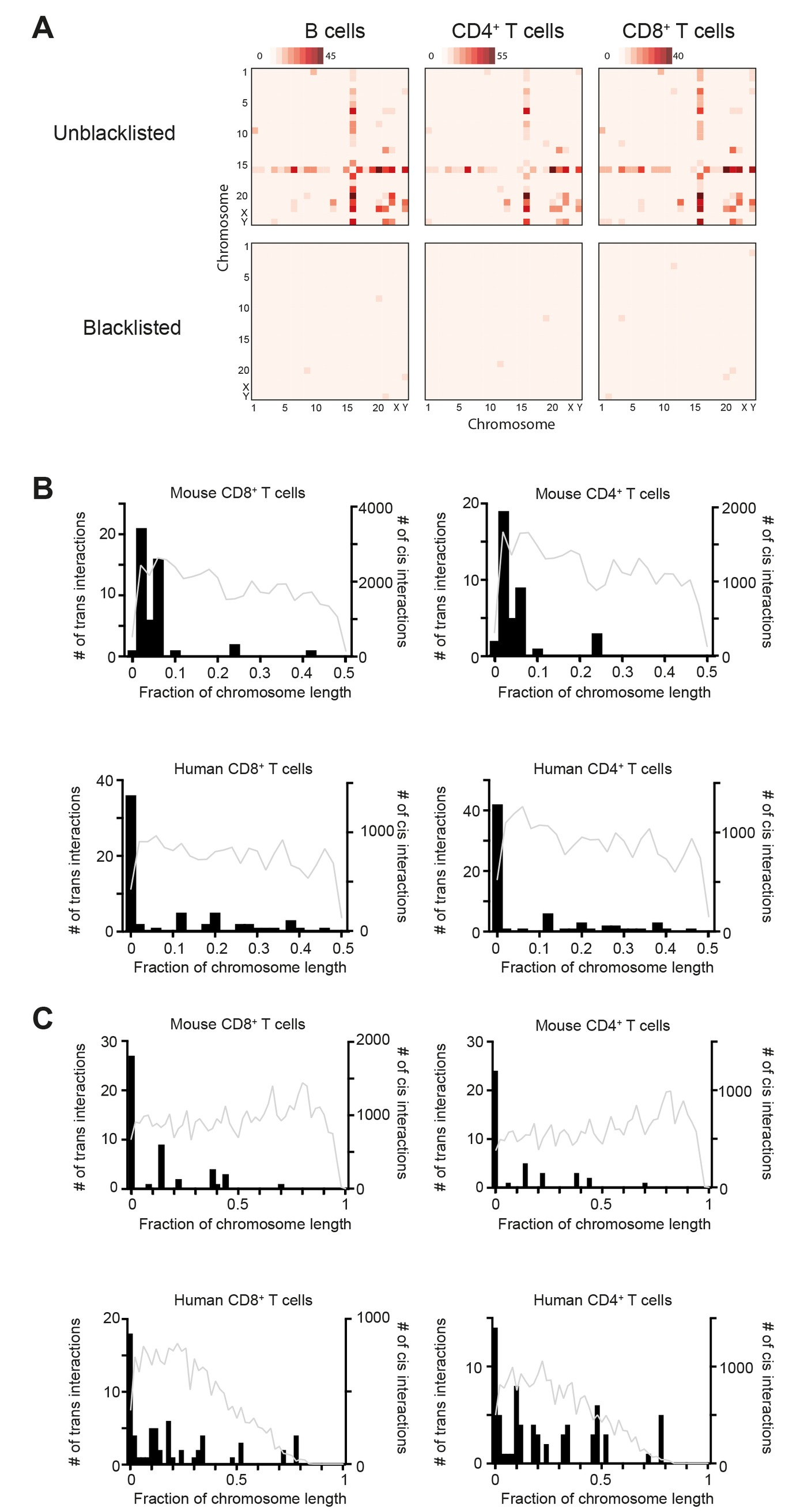

Supplement: S2 Fig — (A) Heatmap of chromosomes involved in detected transchromosomal interactions in human B cells, CD4+ and CD8+ T cells. (B) Association of transchromosomal (black histogram) and intrachromosomal interactions (grey line) in mouse or human CD8+ or CD4+ T cells with telomeres. The x-axis is normalised to chromosome length starting from the telomere. (C) Association of transchromosomal (black histogram) and intrachromosomal interactions (grey line) in mouse or human CD8+ or CD4+ T cells with centromeres. The x-axis is normalised to chromosome length starting from the centromere. (TIF) [file pgen.1007431.s002.tif]

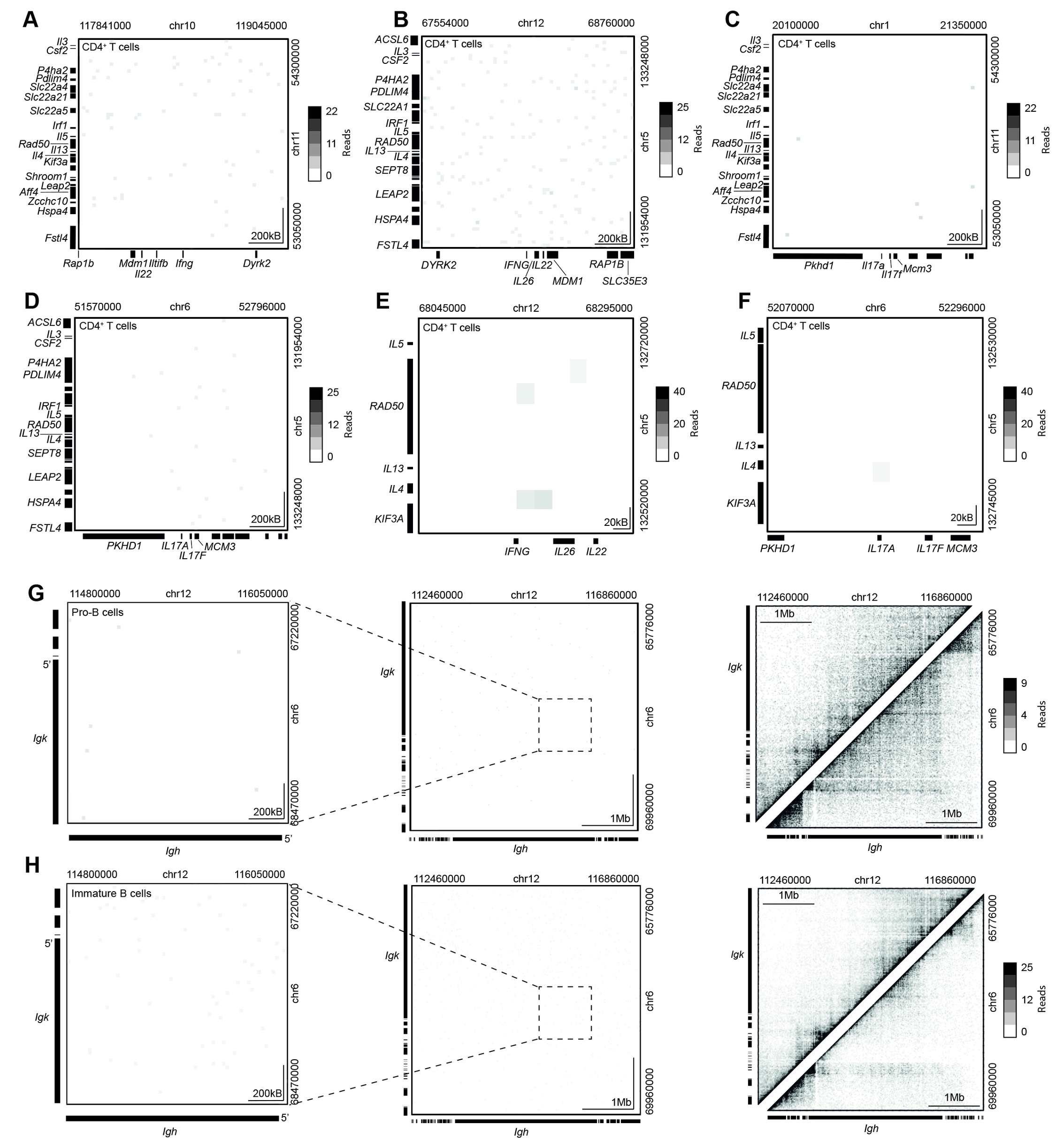

Supplement: S3 Fig — (A) HiC contact matrix of unfiltered data of regions on chromosome 10 and 11 in mouse CD4+ T cells previously reported to interact. Colour intensity represents interaction with white being absence of detected interaction and black being intense interaction. Pixels are 20kB. (B) HiC contact matrix of unfiltered data of regions on chromosome 12 and 5 in human CD4+ T cells previously reported to interact. (C) HiC contact matrix of unfiltered data of regions on chromosome 1 and 11 in mouse CD4+ T cells previously reported to interact. (D) HiC contact matrix of unfiltered data of regions on chromosome 6 and 5 in human CD4+ T cells previously reported to interact. (E) Promoter capture HiC contact matrix [15] of regions on chromosome 12 and 5 in human CD4+ T cells previously reported to interact. (F) Promoter capture HiC contact matrix [15] of regions on chromosome 6 and 5 in human CD4+ T cells previously reported to interact. (G) HiC contact matrices of regions on chromosome 12 and 6 in mouse pro-B cells previously reported to interact in these cells. The left panel is an expanded plot of the region enclosed by the dotted square in the central panel. The right panel shows the intrachromosomal interactions in the same regions. (H) HiC contact matrices of regions on chromosome 12 and 6 in mouse immature B cells previously reported to interact in these cells. The left panel is an expanded plot of the region enclosed by the dotted square in the central panel. The right panel shows the intrachromosomal interactions in the same regions. (TIF) [file pgen.1007431.s003.tif]
