# Supplementary material for: Genome-wide analysis reveals no evidence of trans chromosomal regulation of mammalian immune development
Source: PLoS Genet. 2018 Jun 8;14(6):e1007431. doi: 10.1371/journal.pgen.1007431 (PMC6010296; doi:10.1371/journal.pgen.1007431)
Supplement: S1 Table — (PDF) [file pgen.1007431.s004.pdf]

Supplemental Table 1: Detected transchromosomal interactions in human B cells							
Chromosome	Start	End	Chromosome	Start	End	Gene associated with first anchor	Gene associated with second anchor
chr10	38250149	38299893	chr1	242300215	242350177		PLD5
chr10	38299890	38350019	chr1	242249759	242300218		PLD5
chr10	41800237	41849796	chr1	125150424	125184388		NA,TRN-GTT7-1,TRN-GTT11-2
chr10	41800237	41849796	chr1	143199926	143250600		NA,TRN-GTT7-1,TRN-GTT11-2
chr10	41800237	41849796	chr1	143250597	143299572		NA,TRN-GTT7-1,TRN-GTT11-2
chr10	41849793	41903239	chr1	125150424	125184388		NA,TRN-GTT7-1,TRN-GTT11-2
chr10	41849793	41903239	chr1	143199926	143250600		NA,TRN-GTT7-1,TRN-GTT11-2
chr10	41849793	41903239	chr1	143250597	143299572		NA,TRN-GTT7-1,TRN-GTT11-2
chr10	42066912	42099793	chr1	125150424	125184388		NA,TRN-GTT7-1,TRN-GTT11-2
chr10	42066912	42099793	chr1	143199926	143250600		NA,TRN-GTT7-1,TRN-GTT11-2
chr10	42066912	42099793	chr1	143250597	143299572		NA,TRN-GTT7-1,TRN-GTT11-2
chr12	132999908	133050006	chr4	350263	399918	ZNF26,ZNF84,LOC101928597	ZNF141
chr12	133050003	133100009	chr4	350263	399918	ZNF84,ZNF140	ZNF141
chr12	133050003	133100009	chr4	450109	499872	ZNF84,ZNF140	PIGG,ABCA11P,ZNF721
chr12	133100006	133149598	chr4	150241	199975	ZNF10,ZNF140,ZNF891	ZNF718
chr12	133100006	133149598	chr4	299942	350266	ZNF10,ZNF140,ZNF891	ZNF141,ZNF732,MIR571
chr13	18200335	18249767	chr3	169550393	169599809	FAM230C	MECOM
chr13	114300127	114349859	chr4	190100265	190173349	UPF3A,CHAMP1,LINC01054	
chr13	18200335	18249767	chr6	118600560	118649801	FAM230C	CEP85L
chr13	18200335	18249767	chr7	152400017	152450104	FAM230C	KMT2C,FABP5P3
chr13	18200335	18249767	chr8	94649576	94700104	FAM230C	ESRP1
chr15	20349834	20399498	chr2	13400203	13449937	NA	
chr16	34571526	34599812	chr1	125150424	125184388		NA,TRN-GTT7-1,TRN-GTT11-2
chr16	34571526	34599812	chr1	143199926	143250600		NA,TRN-GTT7-1,TRN-GTT11-2
chr16	34571526	34599812	chr1	143250597	143299572		NA,TRN-GTT7-1,TRN-GTT11-2
chr16	46381705	46400141	chr1	121750817	121795330		NA,TRN-GTT7-1,TRN-GTT11-2
chr16	46381705	46400141	chr1	125150424	125184388		NA,TRN-GTT7-1,TRN-GTT11-2
chr16	46381705	46400141	chr1	143199926	143250600		NA,TRN-GTT7-1,TRN-GTT11-2

chr16	46381705	46400141	chr1	143250597	143299572	NA,TRN-GTT7-1,TRN-GTT11-2
chr16	46400138	46449969	chr1	125150424	125184388	NA,TRN-GTT7-1,TRN-GTT11-2
chr16	46400138	46449969	chr1	143199926	143250600	NA,TRN-GTT7-1,TRN-GTT11-2
chr16	46400138	46449969	chr1	143250597	143299572	NA,TRN-GTT7-1,TRN-GTT11-2
chr16	34571526	34599812	chr10	38527180	38598567	
chr16	34571526	34599812	chr10	38901593	38949591	
chr16	34571526	34599812	chr10	38949588	38999880	
chr16	34571526	34599812	chr10	39349650	39399138	
chr16	34571526	34599812	chr10	41800237	41849796	
chr16	34571526	34599812	chr10	41849793	41903239	
chr16	34571526	34599812	chr10	42066912	42099793	
chr16	46381705	46400141	chr10	38527180	38598567	
chr16	46381705	46400141	chr10	38901593	38949591	
chr16	46381705	46400141	chr10	38949588	38999880	
chr16	46381705	46400141	chr10	39349650	39399138	
chr16	46381705	46400141	chr10	41800237	41849796	
chr16	46381705	46400141	chr10	41849793	41903239	
chr16	46381705	46400141	chr10	42066912	42099793	
chr16	46400138	46449969	chr10	38527180	38598567	
chr16	46400138	46449969	chr10	41800237	41849796	
chr16	46400138	46449969	chr10	41849793	41903239	
chr16	46400138	46449969	chr10	42066912	42099793	
chr16	34571526	34599812	chr11	54550771	54599999	
chr16	34571526	34599812	chr11	54599996	54650482	OR4C46
chr16	34571526	34599812	chr11	55450163	55500044	
chr16	34571526	34599812	chr11	132200121	132249718	NTM
chr16	46381705	46400141	chr11	50500547	50550964	
chr16	46381705	46400141	chr11	54700022	54750790	OR4A5
chr16	46381705	46400141	chr11	54799991	54849895	
chr16	46381705	46400141	chr11	132400205	132450186	OPCML
chr16	34571526	34599812	chr12	34299583	34350089	
chr16	34571526	34599812	chr12	34350086	34400717	

chr16	34571526	34599812	chr12	34599960	34648905	
chr16	46381705	46400141	chr12	34299583	34350089	
chr16	46381705	46400141	chr12	34599960	34648905	
chr16	46381705	46400141	chr12	34648902	34699987	
chr16	34571526	34599812	chr13	18200335	18249767	FAM230C
chr16	46381705	46400141	chr13	18200335	18249767	FAM230C
chr16	46400138	46449969	chr13	18200335	18249767	FAM230C
chr16	34571526	34599812	chr14	98249696	98300156	
chr16	46381705	46400141	chr14	18649986	18699907	
chr16	46381705	46400141	chr14	98150205	98200072	
chr16	46381705	46400141	chr14	98800218	98849843	
chr16	33600042	33649866	chr15	20199888	20250373	
chr16	34571526	34599812	chr15	17000532	17049884	
chr16	34571526	34599812	chr15	17049881	17099598	
chr16	34571526	34599812	chr15	20199888	20250373	
chr16	34571526	34599812	chr15	20250370	20300061	CHEK2P2,NA
chr16	34571526	34599812	chr15	20349834	20399498	NA
chr16	46381705	46400141	chr15	17000532	17049884	
chr16	46381705	46400141	chr15	17049881	17099598	
chr16	46381705	46400141	chr15	20199888	20250373	
chr16	46381705	46400141	chr15	20250370	20300061	CHEK2P2,NA
chr16	46381705	46400141	chr15	20349834	20399498	NA
chr16	46381705	46400141	chr15	23799840	23850193	
chr16	46381705	46400141	chr15	25149613	25199834	3,SNORD115-4,SNORD115-5,SNORD115-6,SNORD115-7,SNORD115-8,SNORD115-9,SNORD115-10,SNORD115-11,SNORD115-12,SNORD115-
chr16	46381705	46400141	chr15	26699995	26749090	GABRB3
chr16	46381705	46400141	chr15	96149931	96200181	NR2F2-AS1
chr16	46381705	46400141	chr15	96900098	96950076	
chr16	46381705	46400141	chr15	97100305	97149028	
chr16	46381705	46400141	chr15	97499949	97549290	LINC02254

chr16	34571526	34599812	chr2	7599832	7650034	
chr16	34571526	34599812	chr2	89754345	89800402	
chr16	34571526	34599812	chr2	89800399	89849916	
chr16	34571526	34599812	chr2	91404693	91449981	
chr16	46381705	46400141	chr2	7599832	7650034	
chr16	46381705	46400141	chr2	89754345	89800402	
chr16	46381705	46400141	chr2	89800399	89849916	
chr16	46381705	46400141	chr2	90349790	90399647	LOC101060017
chr16	46381705	46400141	chr2	91404693	91449981	
chr16	46400138	46449969	chr2	91404693	91449981	
chr16	34571526	34599812	chr3	75649945	75700347	LINC00960,FRG2C
chr16	46381705	46400141	chr3	75649945	75700347	LINC00960,FRG2C
chr16	46381705	46400141	chr3	75700344	75750279	ZNF717,MIR4273
chr16	34571526	34599812	chr4	8850799	8900096	HMX1
chr16	34571526	34599812	chr4	49049741	49105571	CWH43
chr16	34571526	34599812	chr4	49147262	49199785	
chr16	34571526	34599812	chr4	49499867	49550044	
chr16	34571526	34599812	chr4	49599978	49650186	
chr16	46381705	46400141	chr4	49049741	49105571	CWH43
chr16	46381705	46400141	chr4	49147262	49199785	
chr16	46381705	46400141	chr4	49499867	49550044	
chr16	46381705	46400141	chr4	49550041	49599981	
chr16	46381705	46400141	chr4	49599978	49650186	
chr16	46381705	46400141	chr4	83999923	84050116	LOC101928978
chr16	46400138	46449969	chr4	49499867	49550044	
chr16	46400138	46449969	chr4	49599978	49650186	
chr16	34571526	34599812	chr5	3599893	3650005	IRX1
chr16	34571526	34599812	chr5	49600099	49649612	
chr16	34571526	34599812	chr5	49649609	49700100	
chr16	34571526	34599812	chr5	85799195	85850120	
chr16	46381705	46400141	chr5	2599873	2649964	
chr16	46381705	46400141	chr5	3100394	3149779	

chr16	46381705	46400141	chr5	46099933	46151464		
chr16	46381705	46400141	chr5	49550305	49600102		
chr16	46381705	46400141	chr5	49600099	49649612		
chr16	46381705	46400141	chr5	49649609	49700100		
chr16	33600042	33649866	chr6	300053	350084		DUSP22
chr16	33600042	33649866	chr6	350081	399537		IRF4,DUSP22,NA
chr16	33649863	33699800	chr6	300053	350084		DUSP22
chr16	33649863	33699800	chr6	350081	399537		IRF4,DUSP22,NA
chr16	33699797	33750323	chr6	300053	350084	LOC102724207	DUSP22
chr16	34571526	34599812	chr6	300053	350084		DUSP22
chr16	34571526	34599812	chr6	350081	399537		IRF4,DUSP22,NA
chr16	46381705	46400141	chr6	300053	350084		DUSP22
chr16	46381705	46400141	chr6	350081	399537		IRF4,DUSP22,NA
chr16	46400138	46449969	chr6	300053	350084		DUSP22
chr16	46400138	46449969	chr6	350081	399537		IRF4,DUSP22,NA
chr16	34571526	34599812	chr7	57199982	57250062		LOC105375297
chr16	34571526	34599812	chr7	57349864	57400201		
chr16	34571526	34599812	chr7	58000283	58049866		
chr16	34571526	34599812	chr7	58049863	58099928		
chr16	34571526	34599812	chr7	58099925	58118729		
chr16	34571526	34599812	chr7	60900242	60949799		
chr16	34571526	34599812	chr7	61000013	61050313		NA
chr16	34571526	34599812	chr7	61050310	61099460		
chr16	34571526	34599812	chr7	62300512	62349945		
chr16	34571526	34599812	chr7	62349942	62398972		
chr16	46381705	46400141	chr7	56400006	56450200		LOC650226
chr16	46381705	46400141	chr7	56549706	56599228		
chr16	46381705	46400141	chr7	57300004	57349867		
chr16	46381705	46400141	chr7	57400198	57449974		MIR3147,LOC100653233
chr16	46381705	46400141	chr7	58000283	58049866		
chr16	46381705	46400141	chr7	58099925	58118729		
chr16	46381705	46400141	chr7	60900242	60949799		

chr16	46381705	46400141	chr7	61000013	61050313	NA
chr16	46381705	46400141	chr7	61050310	61099460	
chr16	46381705	46400141	chr7	61899091	61949865	
chr16	46381705	46400141	chr7	62300512	62349945	
chr16	46381705	46400141	chr7	62349942	62398972	
chr16	46381705	46400141	chr7	63000284	63050047	
chr16	46381705	46400141	chr7	152400017	152450104	KMT2C,FABP5P3
chr16	46381705	46400141	chr7	154500198	154549846	DPP6
chr16	46400138	46449969	chr7	58000283	58049866	
chr16	46400138	46449969	chr7	58049863	58099928	
chr16	46400138	46449969	chr7	58099925	58118729	
chr16	46400138	46449969	chr7	60900242	60949799	
chr16	46400138	46449969	chr7	61000013	61050313	NA
chr16	46400138	46449969	chr7	61050310	61099460	
chr16	46400138	46449969	chr7	62300512	62349945	
chr16	46400138	46449969	chr7	62349942	62398972	
chr16	34571526	34599812	chr8	45999815	46050536	
chr16	46381705	46400141	chr8	45999815	46050536	
chr16	46381705	46400141	chr8	46050533	46101800	
chr16	46381705	46400141	chr8	46399461	46449845	
chr16	46381705	46400141	chr8	138449680	138499891	FAM135B
chr16	46381705	46400141	chr8	138499888	138550196	
chr16	34571526	34599812	chr9	40900547	40950215	MIR1299
chr16	34571526	34599812	chr9	43299394	43350642	
chr16	34571526	34599812	chr9	63799579	63849033	FRG1JP,MIR4477B
chr16	34571526	34599812	chr9	65399890	65450185	
chr16	46381705	46400141	chr9	16399958	16449702	BNC2
chr16	46381705	46400141	chr9	40900547	40950215	MIR1299
chr16	46381705	46400141	chr9	43299394	43350642	
chr16	46381705	46400141	chr9	60518655	60551476	
chr16	46381705	46400141	chr9	61599980	61650019	
chr16	46381705	46400141	chr9	61650016	61700040	

chr16	46381705	46400141	chr9	63799579	63849033		FRG1JP,MIR4477B
chr16	46381705	46400141	chr9	63849030	63899980		FRG1JP
chr16	46381705	46400141	chr9	65399890	65450185		
chr16	46381705	46400141	chr9	65450182	65500201		
chr16	46381705	46400141	chr9	116850136	116899788		ASTN2
chr16	46381705	46400141	chr9	117199418	117250265		ASTN2
chr17	22100059	22150046	chr13	62999985	63050165		
chr17	22100059	22150046	chr13	63050162	63100056		
chr17	22150043	22199946	chr13	62999985	63050165		
chr17	11050123	11099903	chr16	46381705	46400141		
chr17	11399660	11449951	chr16	46381705	46400141	SHISA6	
chr17	11449948	11499943	chr16	46381705	46400141	SHISA6	
chr17	11499940	11550121	chr16	34571526	34599812	SHISA6	
chr17	21299759	21350142	chr16	34571526	34599812	MAP2K3	
chr17	21299759	21350142	chr16	46381705	46400141	MAP2K3	
chr17	21400468	21449714	chr16	46381705	46400141	KCNJ12	
chr17	21649835	21700393	chr16	34571526	34599812	KCNJ18	
chr17	21649835	21700393	chr16	46381705	46400141	KCNJ18	
chr17	21795739	21849776	chr16	34571526	34599812		
chr17	21795739	21849776	chr16	46381705	46400141		
chr17	22100059	22150046	chr16	34571526	34599812		
chr17	22100059	22150046	chr16	46381705	46400141		
chr17	22299857	22349988	chr16	46381705	46400141	FAM27E5	
chr17	22399955	22449852	chr16	34571526	34599812	FLJ36000	
chr17	22449849	22500064	chr16	34571526	34599812		
chr17	22549991	22599755	chr16	34571526	34599812		
chr17	22699013	22750392	chr16	46381705	46400141		
chr17	26550768	26599758	chr16	34571526	34599812		
chr17	26599755	26651667	chr16	34571526	34599812		
chr17	26599755	26651667	chr16	46381705	46400141		
chr17	26599755	26651667	chr16	46400138	46449969		
chr17	26853610	26883808	chr16	34571526	34599812		

chr17	26853610	26883808	chr16	46381705	46400141		
chr17	26883805	26949879	chr16	34571526	34599812		
chr17	26883805	26949879	chr16	46381705	46400141		
chr17	53350213	53400068	chr16	46381705	46400141		
chr17	71900154	71950498	chr16	46381705	46400141		
chr17	21795739	21849776	chr7	63000284	63050047		
chr17	62450045	62500009	chr7	128500198	128549626	TLK2,METTL2A,NA	METTL2B
chr18	14400332	14449709	chr13	18599942	18650338		
chr18	100130	149971	chr16	34571526	34599812	ROCK1P1,MIR8078	
chr18	100130	149971	chr16	46381705	46400141	ROCK1P1,MIR8078	
chr18	7200118	7249929	chr16	46381705	46400141	LRRC30	
chr18	22150125	22199945	chr16	34571526	34599812	GATA6,GATA6-AS1	
chr18	22299835	22350046	chr16	34571526	34599812		
chr18	78350051	78399854	chr16	46381705	46400141		
chr18	78749762	78800022	chr16	46381705	46400141		
chr18	80249971	80261931	chr4	190100265	190173349		
chr18	15350147	15398346	chr9	65048966	65078822		
chr19	22299467	22350160	chr16	46381705	46400141	ZNF729	
chr19	22399956	22450040	chr16	46381705	46400141	ZNF98	
chr19	22450037	22499893	chr16	34571526	34599812	LOC105376917	
chr19	22450037	22499893	chr16	46381705	46400141	LOC105376917	
chr19	22749642	22799970	chr16	34571526	34599812	ZNF99	
chr19	23000145	23049949	chr16	46381705	46400141	ZNF728,LINC01859	
chr19	23049946	23100476	chr16	46381705	46400141	LINC01858	
chr19	24349967	24400077	chr16	34571526	34599812		
chr19	24349967	24400077	chr16	46381705	46400141		
chr19	27251090	27300061	chr16	46381705	46400141		
chr19	27300058	27347906	chr16	34571526	34599812		
chr19	27347903	27399930	chr16	34571526	34599812		
chr19	27347903	27399930	chr16	46381705	46400141		
chr19	27499796	27550248	chr16	46381705	46400141	LOC105376906	
chr19	27550245	27600083	chr16	46381705	46400141	LOC105376906	

chr19	28150203	28199839	chr16	34571526	34599812	
chr19	28299695	28350438	chr16	34571526	34599812	
chr19	28399679	28449187	chr16	46381705	46400141	LOC100420587
chr19	28849982	28900182	chr16	34571526	34599812	
chr19	28999850	29049662	chr16	46381705	46400141	LINC01532
chr19	29350132	29399920	chr16	34571526	34599812	LOC284395
chr19	29399917	29449916	chr16	46381705	46400141	LOC284395
chr19	29449913	29500515	chr16	34571526	34599812	LOC284395
chr19	30499988	30550238	chr16	34571526	34599812	ZNF536
chr19	31049908	31099816	chr16	46381705	46400141	
chr19	31349869	31399980	chr16	34571526	34599812	NA
chr19	31600007	31649794	chr16	46381705	46400141	
chr19	24349967	24400077	chr5	49600099	49649612	
chr19	24349967	24400077	chr5	50149752	50202047	
chr20	199977	250026	chr1	257716	297872	DEFB129
chr20	250023	299742	chr1	257716	297872	ZCCHC3,C20orf96,DEFB132
chr20	28841809	28899997	chr15	17049881	17099598	
chr20	7200006	7249980	chr16	46381705	46400141	LINC01428
chr20	22950254	22999935	chr16	34571526	34599812	
chr20	23700064	23749725	chr16	46381705	46400141	CST1
chr20	24299259	24350044	chr16	34571526	34599812	
chr20	24450057	24500630	chr16	46381705	46400141	SYNDIG1,LOC105372585
chr20	24749682	24800179	chr16	34571526	34599812	
chr20	28450988	28497552	chr16	34571526	34599812	
chr20	28549507	28600342	chr16	46381705	46400141	FRG1CP
chr20	28750636	28799609	chr16	34571526	34599812	
chr20	28750636	28799609	chr16	46381705	46400141	
chr20	28799606	28841812	chr16	34571526	34599812	
chr20	28799606	28841812	chr16	46381705	46400141	
chr20	29050337	29100005	chr16	34571526	34599812	FRG1DP
chr20	29299930	29350304	chr16	34099510	34149997	
chr20	29299930	29350304	chr16	34571526	34599812	

chr20	29299930	29350304	chr16	46381705	46400141		
chr20	29399861	29450169	chr16	34571526	34599812	FRG2EP	
chr20	29749995	29799987	chr16	34571526	34599812		
chr20	29749995	29799987	chr16	46381705	46400141		
chr20	29799984	29849785	chr16	46381705	46400141		
chr20	29849782	29899158	chr16	34571526	34599812		
chr20	29849782	29899158	chr16	46381705	46400141		
chr20	30457382	30500222	chr16	46381705	46400141		
chr20	30500219	30549941	chr16	34571526	34599812		
chr20	30500219	30549941	chr16	46381705	46400141		
chr20	30600017	30649635	chr16	46381705	46400141		
chr20	30811919	30850095	chr16	34571526	34599812		
chr20	30811919	30850095	chr16	46381705	46400141		
chr20	30811919	30850095	chr16	46400138	46449969		
chr20	30850092	30900063	chr16	34571526	34599812		
chr20	30850092	30900063	chr16	46381705	46400141		
chr20	31201485	31250812	chr16	34571526	34599812		
chr20	31250809	31299314	chr16	34571526	34599812	DEFB115	
chr20	31250809	31299314	chr16	46381705	46400141	DEFB115	
chr20	31299311	31350416	chr16	34571526	34599812	DEFB116	
chr20	31299311	31350416	chr16	46381705	46400141	DEFB116	
chr20	39700098	39750763	chr16	46381705	46400141		
chr20	42100258	42150055	chr16	34571526	34599812	PTPRT	
chr20	42249829	42300037	chr16	46381705	46400141	PTPRT	
chr20	42699999	42750144	chr16	46381705	46400141	PTPRT	
chr20	55499733	55549184	chr16	46381705	46400141		
chr20	55899454	55950204	chr16	46381705	46400141		
chr20	55950201	55999918	chr16	34571526	34599812	CBLN4	
chr20	59650827	59699916	chr16	46381705	46400141	PHACTR3	
chr20	60550081	60600046	chr16	34571526	34599812	MIR548AG2	
chr20	61000290	61050109	chr16	46381705	46400141		
chr20	29350301	29399864	chr3	75649945	75700347		LINC00960,FRG2C

chr20	29399861	29450169	chr3	75649945	75700347	FRG2EP	LINC00960,FRG2C
chr20	30811919	30850095	chr3	75649945	75700347		LINC00960,FRG2C
chr20	64299662	64334167	chr6	150175	199780	LINC00266-1	LOC285766
chr20	28600339	28650513	chr9	40950212	41000187	FRG1CP	FRG1HP
chr20	28600339	28650513	chr9	63849030	63899980	FRG1CP	FRG1JP
chr20	29050337	29100005	chr9	63799579	63849033	FRG1DP	FRG1JP,MIR4477B
chr20	29600069	29650090	chr9	61700037	61732933		
chr20	29849782	29899158	chr9	63799579	63849033		FRG1JP,MIR4477B
chr20	30811919	30850095	chr9	63799579	63849033		FRG1JP,MIR4477B
chr21	10399951	10449840	chr10	125900079	125949982	BAGE2	FANK1
chr21	5216622	5249633	chr13	18200335	18249767		FAM230C
chr21	9699932	9749962	chr13	18200335	18249767		FAM230C
chr21	9749959	9800076	chr13	18200335	18249767	LINC01667	FAM230C
chr21	9850430	9899522	chr13	18200335	18249767		FAM230C
chr21	9899519	9949540	chr13	18200335	18249767		FAM230C
chr21	10270164	10324331	chr13	18200335	18249767		FAM230C
chr21	10324328	10350227	chr13	18200335	18249767		FAM230C
chr21	10399951	10449840	chr13	18171295	18200338	BAGE2	FAM230C
chr21	10399951	10449840	chr13	18300237	18349910	BAGE2	
chr21	10449837	10500162	chr13	18171295	18200338	BAGE2,LOC105378260	FAM230C
chr21	10449837	10500162	chr13	18249764	18300240	BAGE2,LOC105378260	
chr21	10449837	10500162	chr13	18300237	18349910	BAGE2,LOC105378260	
chr21	10500159	10549836	chr13	18249764	18300240	TPTE,LOC105378260	
chr21	10500159	10549836	chr13	18300237	18349910	TPTE,LOC105378260	
chr21	10549833	10599924	chr13	18249764	18300240	TPTE	
chr21	10599921	10650007	chr13	18249764	18300240	TPTE	
chr21	10750041	10799904	chr13	18300237	18349910		
chr21	12999491	13049950	chr13	18408375	18449981	ANKRD30BP2	
chr21	42949635	42999720	chr13	18200335	18249767	PKNOX1,MIR5692B	FAM230C
chr21	10599921	10650007	chr15	19949994	19998717	TPTE	NA
chr21	5216622	5249633	chr16	34571526	34599812		
chr21	5216622	5249633	chr16	46381705	46400141		

chr21	7250367	7299248	chr16	34571526	34599812		
chr21	7250367	7299248	chr16	46381705	46400141		
chr21	7915747	7951129	chr16	46381705	46400141		
chr21	7951126	7999704	chr16	34571526	34599812		
chr21	7951126	7999704	chr16	46381705	46400141		
chr21	8756716	8799766	chr16	34571526	34599812		
chr21	8756716	8799766	chr16	46381705	46400141		
chr21	8986605	8999954	chr16	46381705	46400141	MIR3687-2,MIR3648-2	
chr21	8999951	9050237	chr16	34571526	34599812	LOC105372731	
chr21	8999951	9050237	chr16	46381705	46400141	LOC105372731	
chr21	9196084	9250605	chr16	34571526	34599812		
chr21	9749959	9800076	chr16	34571526	34599812	LINC01667	
chr21	9749959	9800076	chr16	46381705	46400141	LINC01667	
chr21	9800073	9850433	chr16	34571526	34599812	LINC01667	
chr21	9850430	9899522	chr16	34571526	34599812		
chr21	9949537	10000430	chr16	46381705	46400141		
chr21	10324328	10350227	chr16	34571526	34599812		
chr21	10324328	10350227	chr16	46381705	46400141		
chr21	10350224	10399954	chr16	34571526	34599812		
chr21	10350224	10399954	chr16	46381705	46400141		
chr21	10399951	10449840	chr16	34571526	34599812	BAGE2	
chr21	10399951	10449840	chr16	46381705	46400141	BAGE2	
chr21	10399951	10449840	chr16	46400138	46449969	BAGE2	
chr21	10449837	10500162	chr16	34571526	34599812	BAGE2,LOC105378260	
chr21	10750041	10799904	chr16	34571526	34599812		
chr21	10750041	10799904	chr16	46381705	46400141		
chr21	5216622	5249633	chr17	22100059	22150046		
chr21	9749959	9800076	chr17	22100059	22150046	LINC01667	
chr21	9850430	9899522	chr17	22100059	22150046		
chr21	10399951	10449840	chr17	22100059	22150046	BAGE2	
chr21	12999491	13049950	chr18	15350147	15398346	ANKRD30BP2	
chr21	13400435	13449816	chr18	14700077	14749818	MIR3156-3	ANKRD30B

chr21	13449813	13499787	chr18	14650050	14700080		
chr21	13499784	13549777	chr18	14600085	14650053	LINC01674	
chr21	13999919	14049708	chr18	14097774	14150045		ZNF519
chr21	10449837	10500162	chr2	97149974	97199975	BAGE2,LOC105378260	ANKRD36
chr21	5216622	5249633	chr20	28999722	29050340		
chr21	5216622	5249633	chr20	29050337	29100005		FRG1DP
chr21	5216622	5249633	chr20	29499581	29550142		
chr21	5216622	5249633	chr20	30349871	30400222		FRG1BP
chr21	9749959	9800076	chr20	29050337	29100005	LINC01667	FRG1DP
chr21	10350224	10399954	chr20	28799606	28841812		
chr21	10350224	10399954	chr20	29050337	29100005		FRG1DP
chr21	10399951	10449840	chr20	28799606	28841812	BAGE2	
chr21	10399951	10449840	chr20	29050337	29100005	BAGE2	FRG1DP
chr21	10750041	10799904	chr20	28799606	28841812		
chr21	32399927	32450047	chr3	75400266	75449982	EVA1C	FAM86DP
chr21	10350224	10399954	chr4	49499867	49550044		
chr21	10399951	10449840	chr4	49499867	49550044	BAGE2	
chr21	10350224	10399954	chr7	152400017	152450104		KMT2C,FABP5P3
chr21	10399951	10449840	chr7	152249727	152300220	BAGE2	KMT2C
chr21	10399951	10449840	chr7	152350028	152400020	BAGE2	KMT2C
chr21	10399951	10449840	chr7	152400017	152450104	BAGE2	KMT2C,FABP5P3
chr21	10449837	10500162	chr7	152249727	152300220	BAGE2,LOC105378260	KMT2C
chr21	10449837	10500162	chr7	152350028	152400020	BAGE2,LOC105378260	KMT2C
chr21	10324328	10350227	chr8	94649576	94700104		ESRP1
chr22	10700993	10750376	chr10	125850030	125900082		DHX32,BCCIP,FANK1
chr22	10700993	10750376	chr10	125900079	125949982		FANK1
chr22	10700993	10750376	chr13	18200335	18249767		FAM230C
chr22	10750373	10784643	chr13	18171295	18200338		FAM230C
chr22	10750373	10784643	chr13	18200335	18249767		FAM230C
chr22	11016725	11050158	chr13	18171295	18200338		FAM230C
chr22	11016725	11050158	chr13	18200335	18249767		FAM230C
chr22	11050155	11118991	chr13	18200335	18249767		FAM230C

chr22	18200307	18238906	chr13	18200335	18249767	LOC100996415	FAM230C
chr22	18709576	18750410	chr13	18200335	18249767	LINC01662	FAM230C
chr22	18850026	18899542	chr13	18200335	18249767		FAM230C
chr22	15599993	15650254	chr14	18649986	18699907		
chr22	15650251	15700110	chr14	18649986	18699907	POTEH	
chr22	15650251	15700110	chr14	19750088	19799931	POTEH	OR4N2,OR4M1
chr22	15650251	15700110	chr14	19799928	19850371	POTEH	OR4N2
chr22	10700993	10750376	chr16	34571526	34599812		
chr22	10700993	10750376	chr16	46381705	46400141		
chr22	10750373	10784643	chr16	34571526	34599812		
chr22	10750373	10784643	chr16	46381705	46400141		
chr22	11016725	11050158	chr16	34571526	34599812		
chr22	11016725	11050158	chr16	46381705	46400141		
chr22	11016725	11050158	chr16	46400138	46449969		
chr22	11050155	11118991	chr16	34571526	34599812		
chr22	11300002	11350065	chr16	34571526	34599812		
chr22	11300002	11350065	chr16	46381705	46400141		
chr22	11549683	11600131	chr16	34149994	34200257		LINC00273
chr22	11549683	11600131	chr16	34571526	34599812		
chr22	11549683	11600131	chr16	46381705	46400141		
chr22	11549683	11600131	chr16	46400138	46449969		
chr22	11600128	11631288	chr16	34149994	34200257		LINC00273
chr22	11600128	11631288	chr16	34571526	34599812		
chr22	11600128	11631288	chr16	46381705	46400141		
chr22	11899979	11950208	chr16	34571526	34599812	LOC102723769	
chr22	11899979	11950208	chr16	46381705	46400141	LOC102723769	
chr22	11977552	12049827	chr16	46381705	46400141		
chr22	12150612	12200552	chr16	34571526	34599812		
chr22	12150612	12200552	chr16	46381705	46400141		
chr22	12349531	12399174	chr16	46381705	46400141		
chr22	12549952	12600061	chr16	34571526	34599812		
chr22	12600058	12641510	chr16	34571526	34599812		

chr22	12600058	12641510	chr16	46381705	46400141		
chr22	12641507	12700005	chr16	46381705	46400141		
chr22	16249443	16302927	chr16	34571526	34599812		
chr22	16249443	16302927	chr16	46381705	46400141		
chr22	16302924	16349741	chr16	34571526	34599812		
chr22	16302924	16349741	chr16	46381705	46400141		
chr22	16302924	16349741	chr16	46400138	46449969		
chr22	18709576	18750410	chr16	34571526	34599812	LINC01662	
chr22	18850026	18899542	chr16	34571526	34599812		
chr22	18850026	18899542	chr16	46381705	46400141		
chr22	10750373	10784643	chr2	95899859	95949911		ANKRD36C
chr22	16400393	16449805	chr2	89754345	89800402		
chr22	10924573	10950031	chr20	28549507	28600342	LOC102723780	FRG1CP
chr22	10924573	10950031	chr20	28600339	28650513	LOC102723780	FRG1CP
chr22	10924573	10950031	chr20	29050337	29100005	LOC102723780	FRG1DP
chr22	10924573	10950031	chr20	30811919	30850095	LOC102723780	
chr22	10950028	11016728	chr20	28549507	28600342	LOC102723780	FRG1CP
chr22	10950028	11016728	chr20	28600339	28650513	LOC102723780	FRG1CP
chr22	10950028	11016728	chr20	30811919	30850095	LOC102723780	
chr22	11300002	11350065	chr20	29849782	29899158		
chr22	11300002	11350065	chr20	30811919	30850095		
chr22	11549683	11600131	chr20	28549507	28600342		FRG1CP
chr22	11549683	11600131	chr20	28600339	28650513		FRG1CP
chr22	11549683	11600131	chr20	29299930	29350304		
chr22	11549683	11600131	chr20	29749995	29799987		
chr22	11549683	11600131	chr20	29849782	29899158		
chr22	11549683	11600131	chr20	30811919	30850095		
chr22	11549683	11600131	chr20	30850092	30900063		
chr22	11600128	11631288	chr20	30811919	30850095		
chr22	11600128	11631288	chr20	30850092	30900063		
chr22	11600128	11631288	chr20	30900060	30949737		
chr22	12549952	12600061	chr20	29050337	29100005		FRG1DP

chr22	12549952	12600061	chr20	29499581	29550142		
chr22	12600058	12641510	chr20	28549507	28600342		FRG1CP
chr22	12600058	12641510	chr20	29050337	29100005		FRG1DP
chr22	12600058	12641510	chr20	29100002	29150103		FRG1DP
chr22	12600058	12641510	chr20	29849782	29899158		
chr22	12600058	12641510	chr20	30811919	30850095		
chr22	10700993	10750376	chr21	10350224	10399954		
chr22	10700993	10750376	chr21	10399951	10449840		BAGE2
chr22	10700993	10750376	chr21	10449837	10500162		BAGE2,LOC105378260
chr22	10750373	10784643	chr21	9749959	9800076		LINC01667
chr22	10750373	10784643	chr21	10350224	10399954		
chr22	10750373	10784643	chr21	10399951	10449840		BAGE2
chr22	10750373	10784643	chr21	10449837	10500162		BAGE2,LOC105378260
chr22	10750373	10784643	chr21	10750041	10799904		
chr22	11016725	11050158	chr21	5216622	5249633		
chr22	11016725	11050158	chr21	10324328	10350227		
chr22	11016725	11050158	chr21	10350224	10399954		
chr22	11016725	11050158	chr21	10399951	10449840		BAGE2
chr22	11050155	11118991	chr21	10350224	10399954		
chr22	11050155	11118991	chr21	10399951	10449840		BAGE2
chr22	11549683	11600131	chr21	10399951	10449840		BAGE2
chr22	12549952	12600061	chr21	5216622	5249633		
chr22	12600058	12641510	chr21	5216622	5249633		
chr22	12600058	12641510	chr21	10324328	10350227		
chr22	17000216	17050019	chr21	10324328	10350227	GAB4,CECR7	
chr22	17000216	17050019	chr21	10350224	10399954	GAB4,CECR7	
chr22	18200307	18238906	chr21	10270164	10324331	LOC100996415	
chr22	18709576	18750410	chr21	10270164	10324331	LINC01662	
chr22	18709576	18750410	chr21	10324328	10350227	LINC01662	
chr22	18850026	18899542	chr21	10270164	10324331		
chr22	18850026	18899542	chr21	10324328	10350227		
chr22	11549683	11600131	chr3	75649945	75700347		LINC00960,FRG2C

chr22	10700993	10750376	chr7	152400017	152450104		KMT2C,FABP5P3
chr22	10750373	10784643	chr7	152249727	152300220		KMT2C
chr22	10750373	10784643	chr7	152350028	152400020		KMT2C
chr22	10750373	10784643	chr7	152400017	152450104		KMT2C,FABP5P3
chr22	11016725	11050158	chr7	152400017	152450104		KMT2C,FABP5P3
chr22	11050155	11118991	chr7	152400017	152450104		KMT2C,FABP5P3
chr22	11549683	11600131	chr9	63799579	63849033		FRG1JP,MIR4477B
chr3	75649945	75700347	chr1	125150424	125184388	LINC00960,FRG2C	NA,TRN-GTT7-1,TRN-GTT11-2
chr3	75649945	75700347	chr1	143199926	143250600	LINC00960,FRG2C	NA,TRN-GTT7-1,TRN-GTT11-2
chr5	49600099	49649612	chr1	121750817	121795330		NA,TRN-GTT7-1,TRN-GTT11-2
chr5	49600099	49649612	chr1	125150424	125184388		NA,TRN-GTT7-1,TRN-GTT11-2
chr5	49600099	49649612	chr1	143199926	143250600		NA,TRN-GTT7-1,TRN-GTT11-2
chr5	49600099	49649612	chr1	143250597	143299572		NA,TRN-GTT7-1,TRN-GTT11-2
chrY	11296644	11349949	chr1	125150424	125184388		NA,TRN-GTT7-1,TRN-GTT11-2
chrY	11296644	11349949	chr1	143199926	143250600		NA,TRN-GTT7-1,TRN-GTT11-2
chrY	11296644	11349949	chr1	143250597	143299572		NA,TRN-GTT7-1,TRN-GTT11-2
chrY	56749632	56823171	chr1	143199926	143250600		NA,TRN-GTT7-1,TRN-GTT11-2
chrY	56823168	56850027	chr1	143199926	143250600		NA,TRN-GTT7-1,TRN-GTT11-2
chrY	11296644	11349949	chr10	42066912	42099793		
chrY	56823168	56850027	chr10	125900079	125949982		FANK1
chrY	56850024	56900380	chr10	125850030	125900082		DHX32,BCCIP,FANK1
chrY	56850024	56900380	chr10	125900079	125949982		FANK1
chrY	56699637	56749635	chr13	18200335	18249767		FAM230C
chrY	56749632	56823171	chr13	18200335	18249767		FAM230C
chrY	56850024	56900380	chr13	18200335	18249767		FAM230C
chrY	56850024	56900380	chr13	18300237	18349910		
chrY	10049859	10099764	chr16	46381705	46400141		
chrY	10099761	10150337	chr16	34571526	34599812		
chrY	10099761	10150337	chr16	46381705	46400141		
chrY	10150334	10200172	chr16	34571526	34599812		
chrY	10150334	10200172	chr16	46381705	46400141		
chrY	10649776	10694177	chr16	34571526	34599812		

chrY	10649776	10694177	chr16	46381705	46400141	
chrY	10649776	10694177	chr16	46400138	46449969	
chrY	10749748	10800130	chr16	34571526	34599812	
chrY	10749748	10800130	chr16	46381705	46400141	
chrY	10749748	10800130	chr16	46400138	46449969	
chrY	10800127	10849884	chr16	46381705	46400141	
chrY	10949852	11001474	chr16	34571526	34599812	
chrY	10949852	11001474	chr16	46381705	46400141	
chrY	11296644	11349949	chr16	34571526	34599812	
chrY	11296644	11349949	chr16	46381705	46400141	
chrY	11503537	11551330	chr16	34571526	34599812	
chrY	11503537	11551330	chr16	46381705	46400141	
chrY	11503537	11551330	chr16	46400138	46449969	
chrY	56699637	56749635	chr16	34571526	34599812	
chrY	56699637	56749635	chr16	46381705	46400141	
chrY	56699637	56749635	chr16	46400138	46449969	
chrY	56749632	56823171	chr16	34571526	34599812	
chrY	56749632	56823171	chr16	46381705	46400141	
chrY	56749632	56823171	chr16	46400138	46449969	
chrY	56823168	56850027	chr16	33699797	33750323	LOC102724207
chrY	56823168	56850027	chr16	34049728	34099513	
chrY	56823168	56850027	chr16	34571526	34599812	
chrY	56823168	56850027	chr16	46381705	46400141	
chrY	56823168	56850027	chr16	46400138	46449969	
chrY	56850024	56900380	chr16	33699797	33750323	LOC102724207
chrY	56850024	56900380	chr16	34571526	34599812	
chrY	56850024	56900380	chr16	46381705	46400141	
chrY	56850024	56900380	chr16	46400138	46449969	
chrY	56850024	56900380	chr17	22100059	22150046	
chrY	11296644	11349949	chr18	100130	149971	ROCK1P1,MIR8078
chrY	56823168	56850027	chr18	100130	149971	ROCK1P1,MIR8078
chrY	56823168	56850027	chr2	95899859	95949911	ANKRD36C

chrY	56823168	56850027	chr2	95949908	96000075	ANKRD36C
chrY	56823168	56850027	chr2	97149974	97199975	ANKRD36
chrY	56850024	56900380	chr2	95899859	95949911	ANKRD36C
chrY	11100271	11149871	chr21	8999951	9050237	LOC105372731
chrY	11296644	11349949	chr21	8999951	9050237	LOC105372731
chrY	11296644	11349949	chr21	10350224	10399954	
chrY	11296644	11349949	chr21	10399951	10449840	BAGE2
chrY	11296644	11349949	chr21	10449837	10500162	BAGE2,LOC105378260
chrY	11296644	11349949	chr21	10750041	10799904	
chrY	56699637	56749635	chr21	10270164	10324331	
chrY	56699637	56749635	chr21	10324328	10350227	
chrY	56749632	56823171	chr21	10270164	10324331	
chrY	56749632	56823171	chr21	10324328	10350227	
chrY	56823168	56850027	chr21	7250367	7299248	
chrY	56823168	56850027	chr21	7951126	7999704	
chrY	56823168	56850027	chr21	8999951	9050237	LOC105372731
chrY	56823168	56850027	chr21	10350224	10399954	
chrY	56823168	56850027	chr21	10399951	10449840	BAGE2
chrY	56823168	56850027	chr21	10449837	10500162	BAGE2,LOC105378260
chrY	56823168	56850027	chr21	10750041	10799904	
chrY	56850024	56900380	chr21	5216622	5249633	
chrY	56850024	56900380	chr21	7951126	7999704	
chrY	56850024	56900380	chr21	9749959	9800076	LINC01667
chrY	56850024	56900380	chr21	10350224	10399954	
chrY	56850024	56900380	chr21	10399951	10449840	BAGE2
chrY	56850024	56900380	chr21	10449837	10500162	BAGE2,LOC105378260
chrY	56850024	56900380	chr21	10750041	10799904	
chrY	10099761	10150337	chr22	11549683	11600131	
chrY	11296644	11349949	chr22	10750373	10784643	
chrY	11296644	11349949	chr22	11016725	11050158	
chrY	11296644	11349949	chr22	12641507	12700005	
chrY	56699637	56749635	chr22	18709576	18750410	LINC01662

chrY	56699637	56749635	chr22	18850026	18899542	
chrY	56749632	56823171	chr22	18200307	18238906	LOC100996415
chrY	56749632	56823171	chr22	18709576	18750410	LINC01662
chrY	56749632	56823171	chr22	18850026	18899542	
chrY	56823168	56850027	chr22	10700993	10750376	
chrY	56823168	56850027	chr22	10750373	10784643	
chrY	56823168	56850027	chr22	11016725	11050158	
chrY	56823168	56850027	chr22	11050155	11118991	
chrY	56823168	56850027	chr22	11549683	11600131	
chrY	56850024	56900380	chr22	10700993	10750376	
chrY	56850024	56900380	chr22	10750373	10784643	
chrY	56850024	56900380	chr22	11016725	11050158	
chrY	56850024	56900380	chr22	11050155	11118991	
chrY	56850024	56900380	chr22	11300002	11350065	
chrY	56850024	56900380	chr22	11549683	11600131	
chrY	11296644	11349949	chr3	75649945	75700347	LINC00960,FRG2C
chrY	56823168	56850027	chr3	75649945	75700347	LINC00960,FRG2C
chrY	10800127	10849884	chr4	49147262	49199785	
chrY	11296644	11349949	chr4	49499867	49550044	
chrY	11296644	11349949	chr4	49550041	49599981	
chrY	11296644	11349949	chr4	49599978	49650186	
chrY	56823168	56850027	chr4	49499867	49550044	
chrY	56823168	56850027	chr4	49599978	49650186	
chrY	56850024	56900380	chr4	49499867	49550044	
chrY	56850024	56900380	chr4	49599978	49650186	
chrY	11296644	11349949	chr5	49600099	49649612	
chrY	56823168	56850027	chr7	152400017	152450104	KMT2C,FABP5P3
chrY	56850024	56900380	chr7	152249727	152300220	KMT2C
chrY	56850024	56900380	chr7	152400017	152450104	KMT2C,FABP5P3

Supplemental Table 1: Detected transchromosomal interactions in human CD4+ T cells							
Chromosome	Start	End	Chromosome	Start	End	Gene associated with first anchor	Gene associated with second anchor
chr10	38299890	38350019	chr1	242249759	242300218		PLD5
chr10	41800237	41849796	chr1	125150424	125184388		NA,TRN-GTT7-1,TRN-GTT11-2
chr10	41800237	41849796	chr1	143199926	143250600		NA,TRN-GTT7-1,TRN-GTT11-2
chr10	41800237	41849796	chr1	143250597	143299572		NA,TRN-GTT7-1,TRN-GTT11-2
chr10	41849793	41903239	chr1	125150424	125184388		NA,TRN-GTT7-1,TRN-GTT11-2
chr10	41849793	41903239	chr1	143199926	143250600		NA,TRN-GTT7-1,TRN-GTT11-2
chr10	42066912	42099793	chr1	125150424	125184388		NA,TRN-GTT7-1,TRN-GTT11-2
chr10	42066912	42099793	chr1	143199926	143250600		NA,TRN-GTT7-1,TRN-GTT11-2
chr10	42066912	42099793	chr1	143250597	143299572		NA,TRN-GTT7-1,TRN-GTT11-2
chr10	38598564	38649852	chr2	89754345	89800402		
chr10	42066912	42099793	chr5	49600099	49649612		
chr12	132999908	133050006	chr4	299942	350266	ZNF26,ZNF84,LOC101928597	ZNF141,ZNF732,MIR571
chr12	132999908	133050006	chr4	399915	450112	ZNF26,ZNF84,LOC101928597	ABCA11P,ZNF721
chr12	133199960	133250080	chr4	150241	199975	ZNF268,ANHX	ZNF718
chr12	133199960	133250080	chr4	199972	249570	ZNF268,ANHX	ZNF718,ZNF876P
chr13	18200335	18249767	chr3	169550393	169599809	FAM230C	MECOM
chr13	18200335	18249767	chr4	49599978	49650186	FAM230C	
chr13	18200335	18249767	chr6	118600560	118649801	FAM230C	CEP85L
chr13	18200335	18249767	chr7	152400017	152450104	FAM230C	KMT2C,FABP5P3
chr16	34571526	34599812	chr1	125150424	125184388		NA,TRN-GTT7-1,TRN-GTT11-2
chr16	34571526	34599812	chr1	143199926	143250600		NA,TRN-GTT7-1,TRN-GTT11-2
chr16	34571526	34599812	chr1	143250597	143299572		NA,TRN-GTT7-1,TRN-GTT11-2
chr16	46381705	46400141	chr1	121750817	121795330		NA,TRN-GTT7-1,TRN-GTT11-2
chr16	46381705	46400141	chr1	125150424	125184388		NA,TRN-GTT7-1,TRN-GTT11-2
chr16	46381705	46400141	chr1	143199926	143250600		NA,TRN-GTT7-1,TRN-GTT11-2
chr16	46381705	46400141	chr1	143250597	143299572		NA,TRN-GTT7-1,TRN-GTT11-2
chr16	46400138	46449969	chr1	125150424	125184388		NA,TRN-GTT7-1,TRN-GTT11-2
chr16	46400138	46449969	chr1	143199926	143250600		NA,TRN-GTT7-1,TRN-GTT11-2
chr16	46400138	46449969	chr1	143250597	143299572		NA,TRN-GTT7-1,TRN-GTT11-2
chr16	70999961	71050384	chr1	146650529	146699783	HYDIN	NA,TRN-GTT7-1,TRN-GTT11-2,HYDIN2
chr16	34571526	34599812	chr10	38527180	38598567		
chr16	34571526	34599812	chr10	41800237	41849796		
chr16	34571526	34599812	chr10	41849793	41903239		
chr16	34571526	34599812	chr10	42066912	42099793		
chr16	46381705	46400141	chr10	38527180	38598567		

chr16	46381705	46400141	chr10	38999877	39049828	
chr16	46381705	46400141	chr10	41800237	41849796	
chr16	46381705	46400141	chr10	41849793	41903239	
chr16	46381705	46400141	chr10	42066912	42099793	
chr16	46400138	46449969	chr10	41800237	41849796	
chr16	46400138	46449969	chr10	41849793	41903239	
chr16	46400138	46449969	chr10	42066912	42099793	
chr16	34571526	34599812	chr11	50051120	50100348	
chr16	34571526	34599812	chr11	50400110	50449026	LOC646813
chr16	34571526	34599812	chr11	54550771	54599999	
chr16	46381705	46400141	chr11	50400110	50449026	LOC646813
chr16	46381705	46400141	chr11	54550771	54599999	
chr16	46381705	46400141	chr11	54599996	54650482	OR4C46
chr16	46381705	46400141	chr11	55349746	55400028	OR4A15
chr16	46381705	46400141	chr11	132500023	132550131	OPCML
chr16	34571526	34599812	chr13	18200335	18249767	FAM230C
chr16	46381705	46400141	chr13	18171295	18200338	FAM230C
chr16	46381705	46400141	chr13	18200335	18249767	FAM230C
chr16	46400138	46449969	chr13	18200335	18249767	FAM230C
chr16	34571526	34599812	chr15	17000532	17049884	
chr16	34571526	34599812	chr15	17049881	17099598	
chr16	34571526	34599812	chr15	23799840	23850193	
chr16	46381705	46400141	chr15	17000532	17049884	
chr16	46381705	46400141	chr15	17049881	17099598	
chr16	46381705	46400141	chr15	19949994	19998717	NA
chr16	46381705	46400141	chr15	20250370	20300061	CHEK2P2,NA
chr16	34571526	34599812	chr2	7599832	7650034	
chr16	34571526	34599812	chr2	89754345	89800402	
chr16	34571526	34599812	chr2	89800399	89849916	
chr16	34571526	34599812	chr2	91404693	91449981	
chr16	46381705	46400141	chr2	7599832	7650034	
chr16	46381705	46400141	chr2	89754345	89800402	
chr16	46381705	46400141	chr2	89800399	89849916	
chr16	46381705	46400141	chr2	91404693	91449981	
chr16	46400138	46449969	chr2	91404693	91449981	
chr16	34571526	34599812	chr3	75649945	75700347	LINC00960,FRG2C
chr16	46381705	46400141	chr3	75649945	75700347	LINC00960,FRG2C

chr16	34571526	34599812	chr4	49049741	49105571		CWH43
chr16	34571526	34599812	chr4	49147262	49199785		
chr16	34571526	34599812	chr4	49499867	49550044		
chr16	34571526	34599812	chr4	49550041	49599981		
chr16	34571526	34599812	chr4	49599978	49650186		
chr16	34571526	34599812	chr4	83999923	84050116		LOC101928978
chr16	46381705	46400141	chr4	49049741	49105571		CWH43
chr16	46381705	46400141	chr4	49147262	49199785		
chr16	46381705	46400141	chr4	49499867	49550044		
chr16	46381705	46400141	chr4	49550041	49599981		
chr16	46381705	46400141	chr4	49599978	49650186		
chr16	46400138	46449969	chr4	49147262	49199785		
chr16	46400138	46449969	chr4	49499867	49550044		
chr16	46400138	46449969	chr4	49550041	49599981		
chr16	46400138	46449969	chr4	49599978	49650186		
chr16	34571526	34599812	chr5	3100394	3149779		
chr16	34571526	34599812	chr5	3650002	3700114		
chr16	34571526	34599812	chr5	49600099	49649612		
chr16	34571526	34599812	chr5	49649609	49700100		
chr16	34571526	34599812	chr5	85799195	85850120		
chr16	46381705	46400141	chr5	3199894	3250248		
chr16	46381705	46400141	chr5	49550305	49600102		
chr16	46381705	46400141	chr5	49600099	49649612		
chr16	46381705	46400141	chr5	49649609	49700100		
chr16	46381705	46400141	chr5	85799195	85850120		
chr16	46400138	46449969	chr5	49600099	49649612		
chr16	33600042	33649866	chr6	300053	350084		DUSP22
chr16	33600042	33649866	chr6	350081	399537		IRF4,DUSP22,NA
chr16	33649863	33699800	chr6	300053	350084		DUSP22
chr16	33699797	33750323	chr6	300053	350084	LOC102724207	DUSP22
chr16	34571526	34599812	chr6	250087	300056		DUSP22
chr16	34571526	34599812	chr6	300053	350084		DUSP22
chr16	34571526	34599812	chr6	350081	399537		IRF4,DUSP22,NA
chr16	46381705	46400141	chr6	250087	300056		DUSP22
chr16	46381705	46400141	chr6	300053	350084		DUSP22
chr16	46381705	46400141	chr6	350081	399537		IRF4,DUSP22,NA
chr16	46400138	46449969	chr6	300053	350084		DUSP22

chr16	46400138	46449969	chr6	350081	399537	IRF4,DUSP22,NA
chr16	34571526	34599812	chr7	56450197	56499846	DKFZp434L192,LOC100240728
chr16	34571526	34599812	chr7	56649936	56699574	
chr16	34571526	34599812	chr7	57199982	57250062	LOC105375297
chr16	34571526	34599812	chr7	57250059	57300007	
chr16	34571526	34599812	chr7	58000283	58049866	
chr16	34571526	34599812	chr7	60900242	60949799	
chr16	34571526	34599812	chr7	61000013	61050313	NA
chr16	34571526	34599812	chr7	61050310	61099460	
chr16	34571526	34599812	chr7	61849435	61899094	
chr16	34571526	34599812	chr7	61899091	61949865	
chr16	34571526	34599812	chr7	62300512	62349945	
chr16	34571526	34599812	chr7	62349942	62398972	
chr16	34571526	34599812	chr7	62900085	62949916	
chr16	34571526	34599812	chr7	63050044	63099989	
chr16	34571526	34599812	chr7	152400017	152450104	KMT2C,FABP5P3
chr16	46381705	46400141	chr7	56450197	56499846	DKFZp434L192,LOC100240728
chr16	46381705	46400141	chr7	57199982	57250062	LOC105375297
chr16	46381705	46400141	chr7	57349864	57400201	
chr16	46381705	46400141	chr7	58000283	58049866	
chr16	46381705	46400141	chr7	58049863	58099928	
chr16	46381705	46400141	chr7	58099925	58118729	
chr16	46381705	46400141	chr7	60900242	60949799	
chr16	46381705	46400141	chr7	61000013	61050313	NA
chr16	46381705	46400141	chr7	61050310	61099460	
chr16	46381705	46400141	chr7	61699907	61751039	
chr16	46381705	46400141	chr7	61899091	61949865	
chr16	46381705	46400141	chr7	62300512	62349945	
chr16	46381705	46400141	chr7	62349942	62398972	
chr16	46381705	46400141	chr7	63000284	63050047	
chr16	46381705	46400141	chr7	63050044	63099989	
chr16	46381705	46400141	chr7	71399440	71449996	WBSCR17
chr16	46381705	46400141	chr7	152400017	152450104	KMT2C,FABP5P3
chr16	46400138	46449969	chr7	58000283	58049866	
chr16	46400138	46449969	chr7	60900242	60949799	
chr16	46400138	46449969	chr7	61000013	61050313	NA
chr16	46400138	46449969	chr7	61050310	61099460	

chr16	46400138	46449969	chr7	62300512	62349945		
chr16	46400138	46449969	chr7	62349942	62398972		
chr16	34571526	34599812	chr8	46349804	46399464		
chr16	34571526	34599812	chr9	43299394	43350642		
chr16	34571526	34599812	chr9	61650016	61700040		
chr16	34571526	34599812	chr9	63799579	63849033		FRG1JP,MIR4477B
chr16	34571526	34599812	chr9	65399890	65450185		
chr16	34571526	34599812	chr9	65450182	65500201		
chr16	34571526	34599812	chr9	117199418	117250265		ASTN2
chr16	34571526	34599812	chr9	117349261	117399699		ASTN2
chr16	46381705	46400141	chr9	40900547	40950215		MIR1299
chr16	46381705	46400141	chr9	43299394	43350642		
chr16	46381705	46400141	chr9	61650016	61700040		
chr16	46381705	46400141	chr9	63799579	63849033		FRG1JP,MIR4477B
chr16	46381705	46400141	chr9	65399890	65450185		
chr16	46381705	46400141	chr9	116999798	117049937		ASTN2
chr16	46381705	46400141	chr9	117650041	117700046		LOC101928797
chr16	46381705	46400141	chr9	119800166	119850258		
chr16	46381705	46400141	chr9	119949948	120000356		
chr16	46381705	46400141	chr9	120050123	120099700		
chr17	22100059	22150046	chr13	62999985	63050165		
chr17	22100059	22150046	chr13	63050162	63100056		
chr17	21649835	21700393	chr16	46381705	46400141	KCNJ18	
chr17	21795739	21849776	chr16	34571526	34599812		
chr17	21795739	21849776	chr16	46381705	46400141		
chr17	21849773	21898858	chr16	46381705	46400141		
chr17	22399955	22449852	chr16	34571526	34599812	FLJ36000	
chr17	22449849	22500064	chr16	46381705	46400141		
chr17	22699013	22750392	chr16	46381705	46400141		
chr17	26550768	26599758	chr16	34571526	34599812		
chr17	26599755	26651667	chr16	34571526	34599812		
chr17	26599755	26651667	chr16	46381705	46400141		
chr17	26599755	26651667	chr16	46400138	46449969		
chr17	26853610	26883808	chr16	34571526	34599812		
chr17	26853610	26883808	chr16	46381705	46400141		
chr17	26883805	26949879	chr16	34571526	34599812		
chr17	26883805	26949879	chr16	46381705	46400141		

chr17	21795739	21849776	chr7	63000284	63050047		
chr17	21795739	21849776	chr7	68149862	68200218		
chr18	14400332	14449709	chr13	18599942	18650338		
chr18	100130	149971	chr16	34571526	34599812	ROCK1P1,MIR8078	
chr18	100130	149971	chr16	46381705	46400141	ROCK1P1,MIR8078	
chr18	49982	100133	chr4	190100265	190173349		
chr18	80199883	80249974	chr4	190100265	190173349	PARD6G	
chr18	49982	100133	chr9	138100599	138149875		CACNA1B
chr18	15350147	15398346	chr9	65048966	65078822		
chr19	57550126	57599953	chr12	133100006	133149598	ZNF416,ZNF550,ZIK1,ZNF530	ZNF10,ZNF140,ZNF891
chr19	57650177	57700161	chr12	132999908	133050006	ZNF154,ZNF551,ZSCAN4	ZNF26,ZNF84,LOC101928597
chr19	57700158	57749489	chr12	133149595	133199963	ZNF154,ZNF671,ZNF776	ZNF10,ZNF268
chr19	58049944	58100067	chr12	133149595	133199963	ZNF135,ZSCAN18,ZSCAN1	ZNF10,ZNF268
chr19	58150249	58200159	chr12	133050003	133100009	ZNF274,ZNF329	ZNF84,ZNF140
chr19	58150249	58200159	chr12	133100006	133149598	ZNF274,ZNF329	ZNF10,ZNF140,ZNF891
chr19	58200156	58249738	chr12	133050003	133100009	ZNF274,ZNF544	ZNF84,ZNF140
chr19	58249735	58299565	chr12	133199960	133250080	ZNF8,ZNF544	ZNF268,ANHX
chr19	58550132	58599999	chr12	133199960	133250080	MZF1,UBE2M,TRIM28,CHMP2A,CENPBD1P1,MZF1-AS1,MIR6807	ZNF268,ANHX
chr19	22799967	22849688	chr16	46381705	46400141		
chr19	24349967	24400077	chr16	46381705	46400141		
chr19	27251090	27300061	chr16	34571526	34599812		
chr19	27251090	27300061	chr16	46381705	46400141		
chr19	27347903	27399930	chr16	34571526	34599812		
chr19	27347903	27399930	chr16	46381705	46400141		
chr19	27650224	27700193	chr16	34571526	34599812		
chr19	28199836	28249803	chr16	46381705	46400141		
chr19	28600380	28649391	chr16	34571526	34599812	LOC100420587,LOC101927210,LOC102724908	
chr19	24349967	24400077	chr5	50202044	50250887		
chr20	199977	250026	chr1	257716	297872	DEFB129	
chr20	250023	299742	chr1	257716	297872	ZCCHC3,C20orf96,DEFB132	
chr20	6649629	6700841	chr16	46381705	46400141		
chr20	6700838	6749674	chr16	34571526	34599812		
chr20	6700838	6749674	chr16	46381705	46400141		
chr20	7049489	7100092	chr16	46381705	46400141		
chr20	7350235	7400056	chr16	46381705	46400141	LINC01706,MIR8062	
chr20	9600273	9650196	chr16	46381705	46400141	PAK5	
chr20	12200139	12250171	chr16	46381705	46400141		

chr20	12250168	12300302	chr16	46381705	46400141	
chr20	12300299	12349806	chr16	46381705	46400141	
chr20	14301053	14349990	chr16	46381705	46400141	FLRT3,MACROD2
chr20	14749988	14801189	chr16	34571526	34599812	MACROD2
chr20	17050046	17100224	chr16	34571526	34599812	
chr20	19750084	19799960	chr16	46381705	46400141	RIN2
chr20	19849908	19900263	chr16	46381705	46400141	RIN2
chr20	21800136	21849778	chr16	46381705	46400141	
chr20	21900189	21949772	chr16	34571526	34599812	
chr20	22049574	22099716	chr16	46381705	46400141	LINC01432
chr20	22549369	22599857	chr16	46381705	46400141	FOXA2,LINC00261,LINC01384
chr20	22950254	22999935	chr16	34571526	34599812	
chr20	28450988	28497552	chr16	46381705	46400141	
chr20	28750636	28799609	chr16	34571526	34599812	
chr20	28750636	28799609	chr16	46381705	46400141	
chr20	28799606	28841812	chr16	34571526	34599812	
chr20	28799606	28841812	chr16	46381705	46400141	
chr20	29299930	29350304	chr16	34571526	34599812	
chr20	29299930	29350304	chr16	46381705	46400141	
chr20	29350301	29399864	chr16	34571526	34599812	
chr20	29350301	29399864	chr16	46381705	46400141	
chr20	29399861	29450169	chr16	34571526	34599812	FRG2EP
chr20	29749995	29799987	chr16	34571526	34599812	
chr20	29749995	29799987	chr16	46381705	46400141	
chr20	29799984	29849785	chr16	34571526	34599812	
chr20	29799984	29849785	chr16	46381705	46400141	
chr20	29849782	29899158	chr16	34571526	34599812	
chr20	29849782	29899158	chr16	46381705	46400141	
chr20	30500219	30549941	chr16	34571526	34599812	
chr20	30500219	30549941	chr16	46381705	46400141	
chr20	30811919	30850095	chr16	34571526	34599812	
chr20	30811919	30850095	chr16	46381705	46400141	
chr20	30850092	30900063	chr16	34571526	34599812	
chr20	31158022	31201488	chr16	34571526	34599812	
chr20	31158022	31201488	chr16	46381705	46400141	
chr20	31201485	31250812	chr16	46381705	46400141	
chr20	31250809	31299314	chr16	34571526	34599812	DEFB115

chr20	31250809	31299314	chr16	46381705	46400141	DEFB115	
chr20	31250809	31299314	chr16	46400138	46449969	DEFB115	
chr20	31299311	31350416	chr16	34571526	34599812	DEFB116	
chr20	39449904	39500403	chr16	46381705	46400141		
chr20	42150052	42200095	chr16	34571526	34599812	PTPRT	
chr20	42349905	42400190	chr16	34571526	34599812	PTPRT	
chr20	42699999	42750144	chr16	46381705	46400141	PTPRT	
chr20	55050228	55100215	chr16	46381705	46400141		
chr20	55150096	55199943	chr16	34571526	34599812		
chr20	55499733	55549184	chr16	46381705	46400141		
chr20	55999915	56050079	chr16	46381705	46400141	CBLN4	
chr20	29350301	29399864	chr3	75649945	75700347		LINC00960,FRG2C
chr20	29399861	29450169	chr3	75649945	75700347	FRG2EP	LINC00960,FRG2C
chr20	30811919	30850095	chr9	63799579	63849033		FRG1JP,MIR4477B
chr21	5216622	5249633	chr13	18200335	18249767		FAM230C
chr21	9749959	9800076	chr13	18200335	18249767	LINC01667	FAM230C
chr21	9899519	9949540	chr13	18200335	18249767		FAM230C
chr21	10270164	10324331	chr13	18200335	18249767		FAM230C
chr21	10324328	10350227	chr13	18200335	18249767		FAM230C
chr21	10350224	10399954	chr13	18171295	18200338		FAM230C
chr21	10399951	10449840	chr13	18171295	18200338	BAGE2	FAM230C
chr21	10399951	10449840	chr13	18249764	18300240	BAGE2	
chr21	10449837	10500162	chr13	18171295	18200338	BAGE2,LOC105378260	FAM230C
chr21	10500159	10549836	chr13	18249764	18300240	TPTE,LOC105378260	
chr21	10500159	10549836	chr13	18300237	18349910	TPTE,LOC105378260	
chr21	10549833	10599924	chr13	18249764	18300240	TPTE	
chr21	10549833	10599924	chr13	18300237	18349910	TPTE	
chr21	10599921	10650007	chr13	18249764	18300240	TPTE	
chr21	10599921	10650007	chr13	18300237	18349910	TPTE	
chr21	10750041	10799904	chr13	18200335	18249767		FAM230C
chr21	12999491	13049950	chr13	18408375	18449981	ANKRD30BP2	
chr21	42949635	42999720	chr13	18200335	18249767	PKNOX1,MIR5692B	FAM230C
chr21	46599801	46650009	chr13	114300127	114349859	PRMT2,S100B	UPF3A,CHAMP1,LINC01054
chr21	10599921	10650007	chr15	19949994	19998717	TPTE	NA
chr21	5216622	5249633	chr16	34571526	34599812		
chr21	5216622	5249633	chr16	46381705	46400141		
chr21	7250367	7299248	chr16	34571526	34599812		

chr21	7250367	7299248	chr16	46381705	46400141		
chr21	7915747	7951129	chr16	34571526	34599812		
chr21	7915747	7951129	chr16	46381705	46400141		
chr21	7951126	7999704	chr16	34571526	34599812		
chr21	7951126	7999704	chr16	46381705	46400141		
chr21	8756716	8799766	chr16	34571526	34599812		
chr21	8756716	8799766	chr16	46381705	46400141		
chr21	9196084	9250605	chr16	46381705	46400141		
chr21	9699932	9749962	chr16	46381705	46400141		
chr21	9749959	9800076	chr16	34571526	34599812	LINC01667	
chr21	9749959	9800076	chr16	46381705	46400141	LINC01667	
chr21	9850430	9899522	chr16	46381705	46400141		
chr21	10324328	10350227	chr16	34571526	34599812		
chr21	10324328	10350227	chr16	46381705	46400141		
chr21	10350224	10399954	chr16	34571526	34599812		
chr21	10350224	10399954	chr16	46381705	46400141		
chr21	10399951	10449840	chr16	34571526	34599812	BAGE2	
chr21	10399951	10449840	chr16	46381705	46400141	BAGE2	
chr21	10399951	10449840	chr16	46400138	46449969	BAGE2	
chr21	10449837	10500162	chr16	34571526	34599812	BAGE2,LOC105378260	
chr21	10449837	10500162	chr16	46400138	46449969	BAGE2,LOC105378260	
chr21	10750041	10799904	chr16	34571526	34599812		
chr21	10750041	10799904	chr16	46381705	46400141		
chr21	9549978	9599720	chr17	21649835	21700393		KCNJ18
chr21	9749959	9800076	chr17	22100059	22150046	LINC01667	
chr21	12999491	13049950	chr18	15350147	15398346	ANKRD30BP2	
chr21	13400435	13449816	chr18	14700077	14749818	MIR3156-3	ANKRD30B
chr21	5216622	5249633	chr20	28999722	29050340		
chr21	5216622	5249633	chr20	29050337	29100005		FRG1DP
chr21	5216622	5249633	chr20	29100002	29150103		FRG1DP
chr21	5216622	5249633	chr20	29499581	29550142		
chr21	5216622	5249633	chr20	29849782	29899158		
chr21	5216622	5249633	chr20	30349871	30400222		FRG1BP
chr21	10399951	10449840	chr20	29849782	29899158	BAGE2	
chr21	10750041	10799904	chr20	28799606	28841812		
chr21	32399927	32450047	chr3	75400266	75449982	EVA1C	FAM86DP
chr21	10399951	10449840	chr4	49499867	49550044	BAGE2	

chr21	10399951	10449840	chr4	49599978	49650186	BAGE2	
chr21	10350224	10399954	chr7	152400017	152450104		KMT2C,FABP5P3
chr21	10399951	10449840	chr7	152350028	152400020	BAGE2	KMT2C
chr21	10399951	10449840	chr7	152400017	152450104	BAGE2	KMT2C,FABP5P3
chr21	10449837	10500162	chr7	152249727	152300220	BAGE2,LOC105378260	KMT2C
chr21	10324328	10350227	chr8	94649576	94700104		ESRP1
chr22	12641507	12700005	chr1	143199926	143250600		NA,TRN-GTT7-1,TRN-GTT11-2
chr22	10700993	10750376	chr10	125850030	125900082		DHX32,BCCIP,FANK1
chr22	10700993	10750376	chr10	125900079	125949982		FANK1
chr22	10700993	10750376	chr13	18200335	18249767		FAM230C
chr22	10750373	10784643	chr13	18200335	18249767		FAM230C
chr22	11016725	11050158	chr13	18200335	18249767		FAM230C
chr22	11050155	11118991	chr13	18200335	18249767		FAM230C
chr22	18200307	18238906	chr13	18200335	18249767	LOC100996415	FAM230C
chr22	18709576	18750410	chr13	18200335	18249767	LINC01662	FAM230C
chr22	18850026	18899542	chr13	18200335	18249767		FAM230C
chr22	15599993	15650254	chr14	18649986	18699907		
chr22	15650251	15700110	chr14	19649866	19699865	POTEH	
chr22	10700993	10750376	chr16	34571526	34599812		
chr22	10700993	10750376	chr16	46381705	46400141		
chr22	10750373	10784643	chr16	34571526	34599812		
chr22	10750373	10784643	chr16	46381705	46400141		
chr22	11016725	11050158	chr16	34571526	34599812		
chr22	11016725	11050158	chr16	46381705	46400141		
chr22	11050155	11118991	chr16	34571526	34599812		
chr22	11050155	11118991	chr16	46381705	46400141		
chr22	11300002	11350065	chr16	34571526	34599812		
chr22	11300002	11350065	chr16	46381705	46400141		
chr22	11549683	11600131	chr16	34571526	34599812		
chr22	11549683	11600131	chr16	46381705	46400141		
chr22	11549683	11600131	chr16	46400138	46449969		
chr22	11600128	11631288	chr16	34149994	34200257		LINC00273
chr22	11600128	11631288	chr16	34571526	34599812		
chr22	11600128	11631288	chr16	46381705	46400141		
chr22	11899979	11950208	chr16	46381705	46400141	LOC102723769	
chr22	11977552	12049827	chr16	34571526	34599812		
chr22	11977552	12049827	chr16	46381705	46400141		

chr22	12150612	12200552	chr16	34571526	34599812		
chr22	12200549	12225477	chr16	46381705	46400141		
chr22	12399171	12437718	chr16	46381705	46400141		
chr22	12549952	12600061	chr16	34571526	34599812		
chr22	12600058	12641510	chr16	34571526	34599812		
chr22	12600058	12641510	chr16	46381705	46400141		
chr22	12641507	12700005	chr16	34571526	34599812		
chr22	12641507	12700005	chr16	46381705	46400141		
chr22	12700002	12726204	chr16	46381705	46400141		
chr22	16249443	16302927	chr16	34571526	34599812		
chr22	16249443	16302927	chr16	46381705	46400141		
chr22	16302924	16349741	chr16	34571526	34599812		
chr22	16302924	16349741	chr16	46381705	46400141		
chr22	16302924	16349741	chr16	46400138	46449969		
chr22	18709576	18750410	chr16	34571526	34599812	LINC01662	
chr22	18709576	18750410	chr16	46381705	46400141	LINC01662	
chr22	18850026	18899542	chr16	34571526	34599812		
chr22	18850026	18899542	chr16	46381705	46400141		
chr22	16400393	16449805	chr2	89754345	89800402		
chr22	16400393	16449805	chr2	91850124	91899939		
chr22	10924573	10950031	chr20	28549507	28600342	LOC102723780	FRG1CP
chr22	10924573	10950031	chr20	28600339	28650513	LOC102723780	FRG1CP
chr22	10924573	10950031	chr20	30811919	30850095	LOC102723780	
chr22	10950028	11016728	chr20	28549507	28600342	LOC102723780	FRG1CP
chr22	10950028	11016728	chr20	28600339	28650513	LOC102723780	FRG1CP
chr22	10950028	11016728	chr20	30811919	30850095	LOC102723780	
chr22	11300002	11350065	chr20	29849782	29899158		
chr22	11300002	11350065	chr20	30811919	30850095		
chr22	11350062	11377511	chr20	29849782	29899158		
chr22	11549683	11600131	chr20	28549507	28600342		FRG1CP
chr22	11549683	11600131	chr20	28600339	28650513		FRG1CP
chr22	11549683	11600131	chr20	29299930	29350304		
chr22	11549683	11600131	chr20	29849782	29899158		
chr22	11549683	11600131	chr20	30811919	30850095		
chr22	11549683	11600131	chr20	30850092	30900063		
chr22	11600128	11631288	chr20	28600339	28650513		FRG1CP
chr22	11600128	11631288	chr20	30811919	30850095		

chr22	11600128	11631288	chr20	30900060	30949737		
chr22	12549952	12600061	chr20	29050337	29100005		FRG1DP
chr22	12600058	12641510	chr20	28549507	28600342		FRG1CP
chr22	12600058	12641510	chr20	29100002	29150103		FRG1DP
chr22	12600058	12641510	chr20	29849782	29899158		
chr22	10700993	10750376	chr21	10324328	10350227		
chr22	10700993	10750376	chr21	10350224	10399954		
chr22	10700993	10750376	chr21	10399951	10449840		BAGE2
chr22	10700993	10750376	chr21	10449837	10500162		BAGE2,LOC105378260
chr22	10750373	10784643	chr21	10350224	10399954		
chr22	10750373	10784643	chr21	10399951	10449840		BAGE2
chr22	10750373	10784643	chr21	10449837	10500162		BAGE2,LOC105378260
chr22	10750373	10784643	chr21	10750041	10799904		
chr22	11016725	11050158	chr21	10324328	10350227		
chr22	11016725	11050158	chr21	10350224	10399954		
chr22	11016725	11050158	chr21	10399951	10449840		BAGE2
chr22	11016725	11050158	chr21	10750041	10799904		
chr22	11050155	11118991	chr21	10350224	10399954		
chr22	11050155	11118991	chr21	10399951	10449840		BAGE2
chr22	11549683	11600131	chr21	10399951	10449840		BAGE2
chr22	12300362	12349534	chr21	5216622	5249633		
chr22	12549952	12600061	chr21	5216622	5249633		
chr22	17000216	17050019	chr21	10350224	10399954	GAB4,CECR7	
chr22	18709576	18750410	chr21	10270164	10324331	LINC01662	
chr22	18709576	18750410	chr21	10324328	10350227	LINC01662	
chr22	18709576	18750410	chr21	10350224	10399954	LINC01662	
chr22	18850026	18899542	chr21	10270164	10324331		
chr22	18850026	18899542	chr21	10324328	10350227		
chr22	11899979	11950208	chr4	49199782	49251604	LOC102723769	
chr22	10700993	10750376	chr7	152400017	152450104		KMT2C,FABP5P3
chr22	10750373	10784643	chr7	152249727	152300220		KMT2C
chr22	10750373	10784643	chr7	152350028	152400020		KMT2C
chr22	10750373	10784643	chr7	152400017	152450104		KMT2C,FABP5P3
chr22	11016725	11050158	chr7	152400017	152450104		KMT2C,FABP5P3
chr22	11050155	11118991	chr7	152400017	152450104		KMT2C,FABP5P3
chr22	32149887	32200280	chr9	98700128	98749677	RFPL2,C22orf42	GABBR2,ANKS6
chr3	49649844	49700638	chr1	16599855	16649766	APEH,MST1,BSN,RNF123	MST1P2,NBPF1,CROCCP2,NA

chr3	75649945	75700347	chr1	143199926	143250600	LINC00960,FRG2C	NA,TRN-GTT7-1,TRN-GTT11-2
chr5	49600099	49649612	chr1	125150424	125184388		NA,TRN-GTT7-1,TRN-GTT11-2
chr5	49600099	49649612	chr1	143199926	143250600		NA,TRN-GTT7-1,TRN-GTT11-2
chr5	49600099	49649612	chr1	143250597	143299572		NA,TRN-GTT7-1,TRN-GTT11-2
chr7	62300512	62349945	chr1	143199926	143250600		NA,TRN-GTT7-1,TRN-GTT11-2
chr7	76900021	76950000	chr1	83450098	83499886		NA
chrY	11296644	11349949	chr1	125150424	125184388		NA,TRN-GTT7-1,TRN-GTT11-2
chrY	11296644	11349949	chr1	143199926	143250600		NA,TRN-GTT7-1,TRN-GTT11-2
chrY	11296644	11349949	chr1	143250597	143299572		NA,TRN-GTT7-1,TRN-GTT11-2
chrY	56749632	56823171	chr1	143199926	143250600		NA,TRN-GTT7-1,TRN-GTT11-2
chrY	56823168	56850027	chr1	125150424	125184388		NA,TRN-GTT7-1,TRN-GTT11-2
chrY	56823168	56850027	chr1	143199926	143250600		NA,TRN-GTT7-1,TRN-GTT11-2
chrY	56850024	56900380	chr1	143199926	143250600		NA,TRN-GTT7-1,TRN-GTT11-2
chrY	56850024	56900380	chr10	125850030	125900082		DHX32,BCCIP,FANK1
chrY	56850024	56900380	chr10	125900079	125949982		FANK1
chrY	56699637	56749635	chr13	18200335	18249767		FAM230C
chrY	56749632	56823171	chr13	18200335	18249767		FAM230C
chrY	10099761	10150337	chr16	34571526	34599812		
chrY	10099761	10150337	chr16	46381705	46400141		
chrY	10150334	10200172	chr16	46381705	46400141		
chrY	10649776	10694177	chr16	34571526	34599812		
chrY	10649776	10694177	chr16	46381705	46400141		
chrY	10649776	10694177	chr16	46400138	46449969		
chrY	10749748	10800130	chr16	34571526	34599812		
chrY	10749748	10800130	chr16	46381705	46400141		
chrY	10749748	10800130	chr16	46400138	46449969		
chrY	10800127	10849884	chr16	34571526	34599812		
chrY	10800127	10849884	chr16	46381705	46400141		
chrY	10800127	10849884	chr16	46400138	46449969		
chrY	10949852	11001474	chr16	46381705	46400141		
chrY	10949852	11001474	chr16	46400138	46449969		
chrY	11249766	11296647	chr16	46381705	46400141		
chrY	11296644	11349949	chr16	34571526	34599812		
chrY	11296644	11349949	chr16	46381705	46400141		
chrY	11296644	11349949	chr16	46400138	46449969		
chrY	11503537	11551330	chr16	46381705	46400141		
chrY	56699637	56749635	chr16	34571526	34599812		

chrY	56699637	56749635	chr16	46381705	46400141	
chrY	56699637	56749635	chr16	46400138	46449969	
chrY	56749632	56823171	chr16	34571526	34599812	
chrY	56749632	56823171	chr16	46381705	46400141	
chrY	56749632	56823171	chr16	46400138	46449969	
chrY	56823168	56850027	chr16	33699797	33750323	LOC102724207
chrY	56823168	56850027	chr16	34049728	34099513	
chrY	56823168	56850027	chr16	34571526	34599812	
chrY	56823168	56850027	chr16	46381705	46400141	
chrY	56823168	56850027	chr16	46400138	46449969	
chrY	56850024	56900380	chr16	34571526	34599812	
chrY	56850024	56900380	chr16	46381705	46400141	
chrY	56850024	56900380	chr16	46400138	46449969	
chrY	11296644	11349949	chr18	100130	149971	ROCK1P1,MIR8078
chrY	56823168	56850027	chr2	88750494	88799987	RPIA,ANKRD36BP2
chrY	56823168	56850027	chr2	95899859	95949911	ANKRD36C
chrY	56823168	56850027	chr2	95949908	96000075	ANKRD36C
chrY	56823168	56850027	chr2	97149974	97199975	ANKRD36
chrY	10749748	10800130	chr21	10399951	10449840	BAGE2
chrY	11100271	11149871	chr21	8999951	9050237	LOC105372731
chrY	11249766	11296647	chr21	9196084	9250605	
chrY	11296644	11349949	chr21	10350224	10399954	
chrY	11296644	11349949	chr21	10399951	10449840	BAGE2
chrY	11296644	11349949	chr21	10449837	10500162	BAGE2,LOC105378260
chrY	56699637	56749635	chr21	10270164	10324331	
chrY	56699637	56749635	chr21	10324328	10350227	
chrY	56749632	56823171	chr21	10270164	10324331	
chrY	56749632	56823171	chr21	10324328	10350227	
chrY	56823168	56850027	chr21	9749959	9800076	LINC01667
chrY	56823168	56850027	chr21	10350224	10399954	
chrY	56823168	56850027	chr21	10399951	10449840	BAGE2
chrY	56823168	56850027	chr21	10449837	10500162	BAGE2,LOC105378260
chrY	56850024	56900380	chr21	9749959	9800076	LINC01667
chrY	56850024	56900380	chr21	10350224	10399954	
chrY	56850024	56900380	chr21	10399951	10449840	BAGE2
chrY	56850024	56900380	chr21	10449837	10500162	BAGE2,LOC105378260
chrY	10099761	10150337	chr22	11549683	11600131	

chrY	11296644	11349949	chr22	10700993	10750376	
chrY	11296644	11349949	chr22	10750373	10784643	
chrY	11296644	11349949	chr22	11016725	11050158	
chrY	11296644	11349949	chr22	12641507	12700005	
chrY	56699637	56749635	chr22	18709576	18750410	LINC01662
chrY	56699637	56749635	chr22	18850026	18899542	
chrY	56749632	56823171	chr22	11016725	11050158	
chrY	56749632	56823171	chr22	18709576	18750410	LINC01662
chrY	56749632	56823171	chr22	18850026	18899542	
chrY	56823168	56850027	chr22	10700993	10750376	
chrY	56823168	56850027	chr22	10750373	10784643	
chrY	56823168	56850027	chr22	11016725	11050158	
chrY	56823168	56850027	chr22	11549683	11600131	
chrY	56850024	56900380	chr22	10700993	10750376	
chrY	56850024	56900380	chr22	10750373	10784643	
chrY	56850024	56900380	chr22	11016725	11050158	
chrY	56850024	56900380	chr22	11050155	11118991	
chrY	56850024	56900380	chr22	11549683	11600131	
chrY	11296644	11349949	chr3	75649945	75700347	LINC00960,FRG2C
chrY	11296644	11349949	chr4	49599978	49650186	
chrY	56823168	56850027	chr4	49147262	49199785	
chrY	56823168	56850027	chr4	49599978	49650186	
chrY	56850024	56900380	chr4	49499867	49550044	
chrY	56850024	56900380	chr4	49599978	49650186	
chrY	56850024	56900380	chr7	152350028	152400020	KMT2C
chrY	56850024	56900380	chr7	152400017	152450104	KMT2C,FABP5P3

Supplemental Table 1: Detected transchromosomal interactions in human CD8+ T cells							
Chromosome	Start	End	Chromosome	Start	End	Gene associated with first anchor	Gene associated with second anchor
chr10	38250149	38299893	chr1	242300215	242350177		PLD5
chr10	38299890	38350019	chr1	242249759	242300218		PLD5
chr10	41800237	41849796	chr1	125150424	125184388		NA,TRN-GTT7-1,TRN-GTT11-2
chr10	41800237	41849796	chr1	143199926	143250600		NA,TRN-GTT7-1,TRN-GTT11-2
chr10	41800237	41849796	chr1	143250597	143299572		NA,TRN-GTT7-1,TRN-GTT11-2
chr10	41849793	41903239	chr1	125150424	125184388		NA,TRN-GTT7-1,TRN-GTT11-2
chr10	41849793	41903239	chr1	143199926	143250600		NA,TRN-GTT7-1,TRN-GTT11-2
chr10	41849793	41903239	chr1	143250597	143299572		NA,TRN-GTT7-1,TRN-GTT11-2
chr10	42066912	42099793	chr1	125150424	125184388		NA,TRN-GTT7-1,TRN-GTT11-2
chr10	42066912	42099793	chr1	143199926	143250600		NA,TRN-GTT7-1,TRN-GTT11-2
chr10	42066912	42099793	chr1	143250597	143299572		NA,TRN-GTT7-1,TRN-GTT11-2
chr11	38749473	38799904	chr8	51799829	51850052		PCMTD1,PXDNL
chr12	133050003	133100009	chr4	450109	499872	ZNF84,ZNF140	PIGG,ABCA11P,ZNF721
chr12	133100006	133149598	chr4	350263	399918	ZNF10,ZNF140,ZNF891	ZNF141
chr12	133100006	133149598	chr4	450109	499872	ZNF10,ZNF140,ZNF891	PIGG,ABCA11P,ZNF721
chr12	133149595	133199963	chr4	99992	150244	ZNF10,ZNF268	ZNF718
chr12	133149595	133199963	chr4	350263	399918	ZNF10,ZNF268	ZNF141
chr12	133199960	133250080	chr4	150241	199975	ZNF268,ANHX	ZNF718
chr13	18200335	18249767	chr1	143199926	143250600	FAM230C	NA,TRN-GTT7-1,TRN-GTT11-2
chr13	18200335	18249767	chr3	169550393	169599809	FAM230C	MECOM
chr13	18200335	18249767	chr6	118600560	118649801	FAM230C	CEP85L
chr13	18200335	18249767	chr7	152400017	152450104	FAM230C	KMT2C,FABP5P3
chr16	34571526	34599812	chr1	125150424	125184388		NA,TRN-GTT7-1,TRN-GTT11-2
chr16	34571526	34599812	chr1	143199926	143250600		NA,TRN-GTT7-1,TRN-GTT11-2
chr16	34571526	34599812	chr1	143250597	143299572		NA,TRN-GTT7-1,TRN-GTT11-2
chr16	46381705	46400141	chr1	121750817	121795330		NA,TRN-GTT7-1,TRN-GTT11-2
chr16	46381705	46400141	chr1	125150424	125184388		NA,TRN-GTT7-1,TRN-GTT11-2
chr16	46381705	46400141	chr1	143199926	143250600		NA,TRN-GTT7-1,TRN-GTT11-2
chr16	46381705	46400141	chr1	143250597	143299572		NA,TRN-GTT7-1,TRN-GTT11-2
chr16	46400138	46449969	chr1	125150424	125184388		NA,TRN-GTT7-1,TRN-GTT11-2

chr16	46400138	46449969	chr1	143199926	143250600	NA,TRN-GTT7-1,TRN-GTT11-2
chr16	46400138	46449969	chr1	143250597	143299572	NA,TRN-GTT7-1,TRN-GTT11-2
chr16	34571526	34599812	chr10	38527180	38598567	
chr16	34571526	34599812	chr10	41800237	41849796	
chr16	34571526	34599812	chr10	41849793	41903239	
chr16	34571526	34599812	chr10	42066912	42099793	
chr16	46381705	46400141	chr10	38527180	38598567	
chr16	46381705	46400141	chr10	41800237	41849796	
chr16	46381705	46400141	chr10	41849793	41903239	
chr16	46381705	46400141	chr10	42066912	42099793	
chr16	46400138	46449969	chr10	38527180	38598567	
chr16	46400138	46449969	chr10	41800237	41849796	
chr16	46400138	46449969	chr10	42066912	42099793	
chr16	34571526	34599812	chr11	50500547	50550964	
chr16	34571526	34599812	chr11	53950304	53999958	
chr16	34571526	34599812	chr11	54550771	54599999	
chr16	34571526	34599812	chr11	55149696	55200416	
chr16	34571526	34599812	chr11	79699808	79749974	
chr16	46381705	46400141	chr11	42149418	42200029	LOC100507205
chr16	46381705	46400141	chr11	54550771	54599999	
chr16	46381705	46400141	chr11	54599996	54650482	OR4C46
chr16	46381705	46400141	chr11	54700022	54750790	OR4A5
chr16	46381705	46400141	chr11	55500041	55550186	
chr16	34571526	34599812	chr13	18200335	18249767	FAM230C
chr16	34571526	34599812	chr13	18249764	18300240	
chr16	46381705	46400141	chr13	18200335	18249767	FAM230C
chr16	46400138	46449969	chr13	18200335	18249767	FAM230C
chr16	34571526	34599812	chr15	17049881	17099598	
chr16	34571526	34599812	chr15	20349834	20399498	NA
chr16	34571526	34599812	chr15	26599435	26649846	GABRB3
chr16	46381705	46400141	chr15	17000532	17049884	
chr16	46381705	46400141	chr15	17049881	17099598	

chr16	46381705	46400141	chr15	20250370	20300061	CHEK2P2,NA
chr16	46381705	46400141	chr15	23750009	23799843	
chr16	46381705	46400141	chr15	23899666	23949764	
chr16	34571526	34599812	chr2	7599832	7650034	
chr16	34571526	34599812	chr2	89754345	89800402	
chr16	34571526	34599812	chr2	89800399	89849916	
chr16	34571526	34599812	chr2	91404693	91449981	
chr16	46381705	46400141	chr2	7599832	7650034	
chr16	46381705	46400141	chr2	89754345	89800402	
chr16	46381705	46400141	chr2	89800399	89849916	
chr16	46381705	46400141	chr2	90349790	90399647	LOC101060017
chr16	46381705	46400141	chr2	91404693	91449981	
chr16	46400138	46449969	chr2	91404693	91449981	
chr16	34571526	34599812	chr3	75649945	75700347	LINC00960,FRG2C
chr16	46381705	46400141	chr3	75649945	75700347	LINC00960,FRG2C
chr16	34571526	34599812	chr4	49049741	49105571	CWH43
chr16	34571526	34599812	chr4	49147262	49199785	
chr16	34571526	34599812	chr4	49499867	49550044	
chr16	34571526	34599812	chr4	49550041	49599981	
chr16	34571526	34599812	chr4	49599978	49650186	
chr16	46381705	46400141	chr4	22749901	22799809	GBA3
chr16	46381705	46400141	chr4	49049741	49105571	CWH43
chr16	46381705	46400141	chr4	49147262	49199785	
chr16	46381705	46400141	chr4	49499867	49550044	
chr16	46381705	46400141	chr4	49550041	49599981	
chr16	46381705	46400141	chr4	49599978	49650186	
chr16	46381705	46400141	chr4	58349971	58400134	
chr16	46381705	46400141	chr4	59350070	59400464	
chr16	46381705	46400141	chr4	83999923	84050116	LOC101928978
chr16	46381705	46400141	chr4	187449853	187499928	LOC339975
chr16	46381705	46400141	chr4	188200338	188250035	
chr16	46381705	46400141	chr4	189199897	189249839	

chr16	46381705	46400141	chr4	189249836	189298942		
chr16	46400138	46449969	chr4	49147262	49199785		
chr16	34571526	34599812	chr5	3250245	3299962		
chr16	34571526	34599812	chr5	3800382	3850110		
chr16	34571526	34599812	chr5	4550092	4600332		
chr16	34571526	34599812	chr5	4700011	4750236		
chr16	34571526	34599812	chr5	4849893	4899965		
chr16	34571526	34599812	chr5	49600099	49649612		
chr16	34571526	34599812	chr5	49649609	49700100		
chr16	34571526	34599812	chr5	85799195	85850120		
chr16	46381705	46400141	chr5	3999998	4049937		
chr16	46381705	46400141	chr5	15399643	15449820		
chr16	46381705	46400141	chr5	49600099	49649612		
chr16	46381705	46400141	chr5	49649609	49700100		
chr16	46381705	46400141	chr5	85799195	85850120		
chr16	33600042	33649866	chr6	300053	350084		DUSP22
chr16	33600042	33649866	chr6	350081	399537		IRF4,DUSP22,NA
chr16	33649863	33699800	chr6	300053	350084		DUSP22
chr16	33699797	33750323	chr6	300053	350084	LOC102724207	DUSP22
chr16	34571526	34599812	chr6	250087	300056		DUSP22
chr16	34571526	34599812	chr6	300053	350084		DUSP22
chr16	34571526	34599812	chr6	350081	399537		IRF4,DUSP22,NA
chr16	46381705	46400141	chr6	250087	300056		DUSP22
chr16	46381705	46400141	chr6	300053	350084		DUSP22
chr16	46381705	46400141	chr6	350081	399537		IRF4,DUSP22,NA
chr16	46400138	46449969	chr6	300053	350084		DUSP22
chr16	46400138	46449969	chr6	350081	399537		IRF4,DUSP22,NA
chr16	34571526	34599812	chr7	57250059	57300007		
chr16	34571526	34599812	chr7	58000283	58049866		
chr16	34571526	34599812	chr7	60900242	60949799		
chr16	34571526	34599812	chr7	61000013	61050313		NA
chr16	34571526	34599812	chr7	61050310	61099460		

chr16	34571526	34599812	chr7	62300512	62349945	
chr16	34571526	34599812	chr7	62349942	62398972	
chr16	34571526	34599812	chr7	62398969	62449171	
chr16	34571526	34599812	chr7	152400017	152450104	KMT2C,FABP5P3
chr16	46381705	46400141	chr7	58000283	58049866	
chr16	46381705	46400141	chr7	58049863	58099928	
chr16	46381705	46400141	chr7	58099925	58118729	
chr16	46381705	46400141	chr7	60900242	60949799	
chr16	46381705	46400141	chr7	61000013	61050313	NA
chr16	46381705	46400141	chr7	61050310	61099460	
chr16	46381705	46400141	chr7	61899091	61949865	
chr16	46381705	46400141	chr7	62300512	62349945	
chr16	46381705	46400141	chr7	62349942	62398972	
chr16	46381705	46400141	chr7	152400017	152450104	KMT2C,FABP5P3
chr16	46400138	46449969	chr7	58000283	58049866	
chr16	46400138	46449969	chr7	60900242	60949799	
chr16	46400138	46449969	chr7	61000013	61050313	NA
chr16	46400138	46449969	chr7	61050310	61099460	
chr16	46400138	46449969	chr7	62300512	62349945	
chr16	46400138	46449969	chr7	62349942	62398972	
chr16	46381705	46400141	chr8	46750260	46799739	
chr16	34571526	34599812	chr9	43299394	43350642	
chr16	34571526	34599812	chr9	61700037	61732933	
chr16	46381705	46400141	chr9	41199592	41249800	MIR4477A,LOC105376057
chr16	46381705	46400141	chr9	43299394	43350642	
chr16	46381705	46400141	chr9	61650016	61700040	
chr16	46381705	46400141	chr9	61700037	61732933	
chr16	46381705	46400141	chr9	63799579	63849033	FRG1JP,MIR4477B
chr16	46381705	46400141	chr9	63849030	63899980	FRG1JP
chr16	46381705	46400141	chr9	65399890	65450185	
chr16	46381705	46400141	chr9	65450182	65500201	
chr16	46381705	46400141	chr9	117299896	117349264	ASTN2

chr16	46381705	46400141	chr9	118850074	118900559		
chr16	46381705	46400141	chr9	119700010	119749994		
chr17	22100059	22150046	chr13	62999985	63050165		
chr17	22100059	22150046	chr13	63050162	63100056		
chr17	22150043	22199946	chr13	62999985	63050165		
chr17	11249949	11299941	chr16	46381705	46400141	SHISA6	
chr17	11399660	11449951	chr16	46381705	46400141	SHISA6	
chr17	21649835	21700393	chr16	34571526	34599812	KCNJ18	
chr17	21649835	21700393	chr16	46381705	46400141	KCNJ18	
chr17	21795739	21849776	chr16	34571526	34599812		
chr17	21898855	21949861	chr16	34571526	34599812		
chr17	22100059	22150046	chr16	34571526	34599812		
chr17	22100059	22150046	chr16	46381705	46400141		
chr17	22449849	22500064	chr16	34571526	34599812		
chr17	22650053	22699016	chr16	46381705	46400141		
chr17	22699013	22750392	chr16	46381705	46400141		
chr17	26599755	26651667	chr16	34571526	34599812		
chr17	26599755	26651667	chr16	46381705	46400141		
chr17	26599755	26651667	chr16	46400138	46449969		
chr17	26853610	26883808	chr16	34571526	34599812		
chr17	26853610	26883808	chr16	46381705	46400141		
chr17	26883805	26949879	chr16	34571526	34599812		
chr17	26883805	26949879	chr16	46381705	46400141		
chr17	52450287	52500110	chr16	34571526	34599812		
chr17	71400382	71449765	chr16	46381705	46400141		
chr17	21795739	21849776	chr7	63000284	63050047		
chr17	21795739	21849776	chr7	68149862	68200218		
chr17	62450045	62500009	chr7	128500198	128549626	TLK2,METTL2A,NA	METTL2B
chr18	100130	149971	chr16	34571526	34599812	ROCK1P1,MIR8078	
chr18	100130	149971	chr16	46381705	46400141	ROCK1P1,MIR8078	
chr18	6599979	6650009	chr16	34571526	34599812		
chr18	6999736	7049896	chr16	34571526	34599812	LAMA1	

chr18	7699930	7750046	chr16	46381705	46400141	PTPRM		
chr18	7850102	7899689	chr16	34571526	34599812	PTPRM		
chr18	7899686	7950143	chr16	46381705	46400141	PTPRM		
chr18	80249971	80261931	chr4	190100265	190173349			
chr18	15299582	15350150	chr9	65048966	65078822	LOC644669		
chr18	15350147	15398346	chr9	65048966	65078822			
chr19	22799967	22849688	chr16	34571526	34599812			
chr19	27300058	27347906	chr16	46381705	46400141			
chr19	27347903	27399930	chr16	46381705	46400141			
chr19	27550245	27600083	chr16	46381705	46400141	LOC105376906		
chr19	27950159	27999945	chr16	34571526	34599812			
chr19	28199836	28249803	chr16	46381705	46400141			
chr19	28299695	28350438	chr16	46381705	46400141			
chr19	58500129	58550135	chr16	49876	100016	TRIM28,SLC27A5,ZBTB45		MPG,NPRL3,POLR3K,RHBDF1,SNRNP25,NA
chr19	24349967	24400077	chr5	49600099	49649612			
chr19	24349967	24400077	chr5	50149752	50202047			
chr19	24349967	24400077	chr5	50299816	50349934			
chr19	27347903	27399930	chr5	49600099	49649612			
chr20	28841809	28899997	chr15	17049881	17099598			
chr20	6849679	6899957	chr16	34571526	34599812			
chr20	9149233	9200027	chr16	46381705	46400141	PLCB4		
chr20	12250168	12300302	chr16	34571526	34599812			
chr20	12651360	12700078	chr16	34571526	34599812			
chr20	14601268	14649919	chr16	34571526	34599812	MACROD2,MACROD2-IT1		
chr20	15699868	15749452	chr16	46381705	46400141	MACROD2		
chr20	15999399	16049481	chr16	46381705	46400141	MACROD2		
chr20	16199727	16249980	chr16	46381705	46400141			
chr20	21800136	21849778	chr16	34571526	34599812			
chr20	28750636	28799609	chr16	34571526	34599812			
chr20	28750636	28799609	chr16	46381705	46400141			
chr20	28799606	28841812	chr16	34571526	34599812			
chr20	28799606	28841812	chr16	46381705	46400141			

chr20	29050337	29100005	chr16	34571526	34599812	FRG1DP	
chr20	29100002	29150103	chr16	46381705	46400141	FRG1DP	
chr20	29299930	29350304	chr16	46381705	46400141		
chr20	29350301	29399864	chr16	34571526	34599812		
chr20	29399861	29450169	chr16	34571526	34599812	FRG2EP	
chr20	29399861	29450169	chr16	46381705	46400141	FRG2EP	
chr20	29749995	29799987	chr16	34571526	34599812		
chr20	29749995	29799987	chr16	46381705	46400141		
chr20	29799984	29849785	chr16	34571526	34599812		
chr20	29799984	29849785	chr16	46381705	46400141		
chr20	29849782	29899158	chr16	34571526	34599812		
chr20	29849782	29899158	chr16	46381705	46400141		
chr20	30549938	30600020	chr16	34571526	34599812		
chr20	30811919	30850095	chr16	34571526	34599812		
chr20	30811919	30850095	chr16	46381705	46400141		
chr20	30850092	30900063	chr16	34571526	34599812		
chr20	30850092	30900063	chr16	46381705	46400141		
chr20	31158022	31201488	chr16	46381705	46400141		
chr20	31201485	31250812	chr16	34571526	34599812		
chr20	31201485	31250812	chr16	46381705	46400141		
chr20	31250809	31299314	chr16	34571526	34599812	DEFB115	
chr20	31250809	31299314	chr16	46381705	46400141	DEFB115	
chr20	31299311	31350416	chr16	34571526	34599812	DEFB116	
chr20	42150052	42200095	chr16	46381705	46400141	PTPRT	
chr20	42349905	42400190	chr16	34571526	34599812	PTPRT	
chr20	42799961	42850171	chr16	46381705	46400141	PTPRT	
chr20	54849531	54899903	chr16	46381705	46400141		
chr20	56050076	56100219	chr16	46381705	46400141		
chr20	29350301	29399864	chr3	75649945	75700347		LINC00960,FRG2C
chr20	29399861	29450169	chr3	75649945	75700347	FRG2EP	LINC00960,FRG2C
chr20	64299662	64334167	chr6	150175	199780	LINC00266-1	LOC285766
chr20	28600339	28650513	chr9	40950212	41000187	FRG1CP	FRG1HP

chr20	30811919	30850095	chr9	63799579	63849033		FRG1JP,MIR4477B
chr21	10399951	10449840	chr1	143199926	143250600	BAGE2	NA,TRN-GTT7-1,TRN-GTT11-2
chr21	5216622	5249633	chr13	18200335	18249767		FAM230C
chr21	9749959	9800076	chr13	18200335	18249767	LINC01667	FAM230C
chr21	9850430	9899522	chr13	18200335	18249767		FAM230C
chr21	9949537	10000430	chr13	18200335	18249767		FAM230C
chr21	10270164	10324331	chr13	18200335	18249767		FAM230C
chr21	10324328	10350227	chr13	18200335	18249767		FAM230C
chr21	10350224	10399954	chr13	18171295	18200338		FAM230C
chr21	10399951	10449840	chr13	18171295	18200338	BAGE2	FAM230C
chr21	10449837	10500162	chr13	18171295	18200338	BAGE2,LOC105378260	FAM230C
chr21	10500159	10549836	chr13	18249764	18300240	TPTE,LOC105378260	
chr21	10500159	10549836	chr13	18300237	18349910	TPTE,LOC105378260	
chr21	10549833	10599924	chr13	18249764	18300240	TPTE	
chr21	10549833	10599924	chr13	18300237	18349910	TPTE	
chr21	10599921	10650007	chr13	18249764	18300240	TPTE	
chr21	10750041	10799904	chr13	18200335	18249767		FAM230C
chr21	12999491	13049950	chr13	18408375	18449981	ANKRD30BP2	
chr21	12999491	13049950	chr13	18950582	18999831	ANKRD30BP2	NA
chr21	42949635	42999720	chr13	18200335	18249767	PKNOX1,MIR5692B	FAM230C
chr21	46450088	46499859	chr13	114300127	114349859	DIP2A,DIP2A-IT1	UPF3A,CHAMP1,LINC01054
chr21	46599801	46650009	chr13	114300127	114349859	PRMT2,S100B	UPF3A,CHAMP1,LINC01054
chr21	10599921	10650007	chr15	19949994	19998717	TPTE	NA
chr21	5216622	5249633	chr16	34571526	34599812		
chr21	5216622	5249633	chr16	46381705	46400141		
chr21	7250367	7299248	chr16	34571526	34599812		
chr21	7250367	7299248	chr16	46381705	46400141		
chr21	7915747	7951129	chr16	34571526	34599812		
chr21	7915747	7951129	chr16	46381705	46400141		
chr21	7951126	7999704	chr16	34571526	34599812		
chr21	7951126	7999704	chr16	46381705	46400141		
chr21	8756716	8799766	chr16	34571526	34599812		

chr21	8756716	8799766	chr16	46381705	46400141		
chr21	9196084	9250605	chr16	46381705	46400141		
chr21	9699932	9749962	chr16	34571526	34599812		
chr21	9699932	9749962	chr16	46381705	46400141		
chr21	9749959	9800076	chr16	34571526	34599812	LINC01667	
chr21	9749959	9800076	chr16	46381705	46400141	LINC01667	
chr21	9850430	9899522	chr16	34571526	34599812		
chr21	9850430	9899522	chr16	46381705	46400141		
chr21	10324328	10350227	chr16	34571526	34599812		
chr21	10324328	10350227	chr16	46381705	46400141		
chr21	10324328	10350227	chr16	46400138	46449969		
chr21	10350224	10399954	chr16	34571526	34599812		
chr21	10350224	10399954	chr16	46381705	46400141		
chr21	10399951	10449840	chr16	34571526	34599812	BAGE2	
chr21	10399951	10449840	chr16	46381705	46400141	BAGE2	
chr21	10399951	10449840	chr16	46400138	46449969	BAGE2	
chr21	10750041	10799904	chr16	34571526	34599812		
chr21	10750041	10799904	chr16	46381705	46400141		
chr21	12999491	13049950	chr16	46381705	46400141	ANKRD30BP2	
chr21	40199719	40249911	chr16	46381705	46400141	DSCAM,MIR4760	
chr21	40500001	40549985	chr16	46381705	46400141	DSCAM	
chr21	40699631	40750009	chr16	46381705	46400141	DSCAM	
chr21	9749959	9800076	chr17	22100059	22150046	LINC01667	
chr21	13400435	13449816	chr18	14700077	14749818	MIR3156-3	ANKRD30B
chr21	13449813	13499787	chr18	14650050	14700080		
chr21	10449837	10500162	chr2	88750494	88799987	BAGE2,LOC105378260	RPIA,ANKRD36BP2
chr21	10449837	10500162	chr2	95899859	95949911	BAGE2,LOC105378260	ANKRD36C
chr21	5216622	5249633	chr20	28999722	29050340		
chr21	5216622	5249633	chr20	29050337	29100005		FRG1DP
chr21	5216622	5249633	chr20	29499581	29550142		
chr21	5216622	5249633	chr20	29849782	29899158		
chr21	5216622	5249633	chr20	30349871	30400222		FRG1BP

chr21	9749959	9800076	chr20	30811919	30850095	LINC01667	
chr21	10399951	10449840	chr20	28799606	28841812	BAGE2	
chr21	10399951	10449840	chr20	29050337	29100005	BAGE2	FRG1DP
chr21	10399951	10449840	chr4	49499867	49550044	BAGE2	
chr21	10449837	10500162	chr4	49599978	49650186	BAGE2,LOC105378260	
chr21	10350224	10399954	chr7	152400017	152450104		KMT2C,FABP5P3
chr21	10399951	10449840	chr7	152249727	152300220	BAGE2	KMT2C
chr21	10399951	10449840	chr7	152350028	152400020	BAGE2	KMT2C
chr21	10399951	10449840	chr7	152400017	152450104	BAGE2	KMT2C,FABP5P3
chr21	10449837	10500162	chr7	152249727	152300220	BAGE2,LOC105378260	KMT2C
chr21	10449837	10500162	chr7	152350028	152400020	BAGE2,LOC105378260	KMT2C
chr21	10324328	10350227	chr8	94649576	94700104		ESRP1
chr22	10700993	10750376	chr10	125850030	125900082		DHX32,BCCIP,FANK1
chr22	10700993	10750376	chr10	125900079	125949982		FANK1
chr22	10750373	10784643	chr13	18200335	18249767		FAM230C
chr22	11016725	11050158	chr13	18200335	18249767		FAM230C
chr22	11050155	11118991	chr13	18200335	18249767		FAM230C
chr22	18200307	18238906	chr13	18200335	18249767	LOC100996415	FAM230C
chr22	18709576	18750410	chr13	18200335	18249767	LINC01662	FAM230C
chr22	18850026	18899542	chr13	18200335	18249767		FAM230C
chr22	15599993	15650254	chr14	18649986	18699907		
chr22	15650251	15700110	chr14	19150120	19200142	POTEH	
chr22	15650251	15700110	chr14	19750088	19799931	POTEH	OR4N2,OR4M1
chr22	10700993	10750376	chr16	34571526	34599812		
chr22	10700993	10750376	chr16	46381705	46400141		
chr22	10750373	10784643	chr16	34571526	34599812		
chr22	10750373	10784643	chr16	46381705	46400141		
chr22	11016725	11050158	chr16	34571526	34599812		
chr22	11016725	11050158	chr16	46381705	46400141		
chr22	11050155	11118991	chr16	34571526	34599812		
chr22	11050155	11118991	chr16	46381705	46400141		
chr22	11300002	11350065	chr16	34571526	34599812		

chr22	11300002	11350065	chr16	46381705	46400141		
chr22	11549683	11600131	chr16	34571526	34599812		
chr22	11549683	11600131	chr16	46381705	46400141		
chr22	11549683	11600131	chr16	46400138	46449969		
chr22	11600128	11631288	chr16	34149994	34200257		LINC00273
chr22	11600128	11631288	chr16	34571526	34599812		
chr22	11600128	11631288	chr16	46381705	46400141		
chr22	11899979	11950208	chr16	34571526	34599812	LOC102723769	
chr22	11899979	11950208	chr16	46381705	46400141	LOC102723769	
chr22	11977552	12049827	chr16	34571526	34599812		
chr22	11977552	12049827	chr16	46381705	46400141		
chr22	12150612	12200552	chr16	46381705	46400141		
chr22	12549952	12600061	chr16	46381705	46400141		
chr22	12600058	12641510	chr16	34571526	34599812		
chr22	12600058	12641510	chr16	46381705	46400141		
chr22	16249443	16302927	chr16	34571526	34599812		
chr22	16249443	16302927	chr16	46381705	46400141		
chr22	16302924	16349741	chr16	34571526	34599812		
chr22	16302924	16349741	chr16	46381705	46400141		
chr22	16302924	16349741	chr16	46400138	46449969		
chr22	18709576	18750410	chr16	34571526	34599812	LINC01662	
chr22	18709576	18750410	chr16	46381705	46400141	LINC01662	
chr22	18850026	18899542	chr16	34571526	34599812		
chr22	18850026	18899542	chr16	46381705	46400141		
chr22	33500012	33549733	chr16	46381705	46400141	LARGE1	
chr22	16400393	16449805	chr2	89754345	89800402		
chr22	16400393	16449805	chr2	91850124	91899939		
chr22	10924573	10950031	chr20	28549507	28600342	LOC102723780	FRG1CP
chr22	10924573	10950031	chr20	28600339	28650513	LOC102723780	FRG1CP
chr22	10924573	10950031	chr20	30811919	30850095	LOC102723780	
chr22	10950028	11016728	chr20	28600339	28650513	LOC102723780	FRG1CP
chr22	11549683	11600131	chr20	28549507	28600342		FRG1CP

chr22	11549683	11600131	chr20	28600339	28650513		FRG1CP
chr22	11549683	11600131	chr20	29299930	29350304		
chr22	11549683	11600131	chr20	29849782	29899158		
chr22	11549683	11600131	chr20	30811919	30850095		
chr22	11549683	11600131	chr20	30850092	30900063		
chr22	11600128	11631288	chr20	30811919	30850095		
chr22	11600128	11631288	chr20	30900060	30949737		
chr22	12549952	12600061	chr20	29050337	29100005		FRG1DP
chr22	12600058	12641510	chr20	28549507	28600342		FRG1CP
chr22	12600058	12641510	chr20	29100002	29150103		FRG1DP
chr22	12600058	12641510	chr20	29849782	29899158		
chr22	10700993	10750376	chr21	10324328	10350227		
chr22	10700993	10750376	chr21	10350224	10399954		
chr22	10700993	10750376	chr21	10399951	10449840		BAGE2
chr22	10700993	10750376	chr21	10449837	10500162		BAGE2,LOC105378260
chr22	10700993	10750376	chr21	10750041	10799904		
chr22	10750373	10784643	chr21	5216622	5249633		
chr22	10750373	10784643	chr21	9749959	9800076		LINC01667
chr22	10750373	10784643	chr21	10350224	10399954		
chr22	10750373	10784643	chr21	10399951	10449840		BAGE2
chr22	10750373	10784643	chr21	10449837	10500162		BAGE2,LOC105378260
chr22	11016725	11050158	chr21	10324328	10350227		
chr22	11016725	11050158	chr21	10350224	10399954		
chr22	11016725	11050158	chr21	10399951	10449840		BAGE2
chr22	11050155	11118991	chr21	10350224	10399954		
chr22	11050155	11118991	chr21	10399951	10449840		BAGE2
chr22	12549952	12600061	chr21	5216622	5249633		
chr22	17000216	17050019	chr21	10350224	10399954	GAB4,CECR7	
chr22	18709576	18750410	chr21	10270164	10324331	LINC01662	
chr22	18709576	18750410	chr21	10324328	10350227	LINC01662	
chr22	18850026	18899542	chr21	10270164	10324331		
chr22	18850026	18899542	chr21	10324328	10350227		

chr22	10700993	10750376	chr7	152400017	152450104		KMT2C,FABP5P3
chr22	10750373	10784643	chr7	152249727	152300220		KMT2C
chr22	10750373	10784643	chr7	152350028	152400020		KMT2C
chr22	10750373	10784643	chr7	152400017	152450104		KMT2C,FABP5P3
chr22	11016725	11050158	chr7	152400017	152450104		KMT2C,FABP5P3
chr22	11050155	11118991	chr7	152350028	152400020		KMT2C
chr22	11050155	11118991	chr7	152400017	152450104		KMT2C,FABP5P3
chr22	11549683	11600131	chr9	63799579	63849033		FRG1JP,MIR4477B
chr22	32149887	32200280	chr9	98700128	98749677	RFPL2,C22orf42	GABBR2,ANKS6
chr3	49649844	49700638	chr1	16599855	16649766	APEH,MST1,BSN,RNF123	MST1P2,NBPF1,CROCCP2,NA
chr3	75649945	75700347	chr1	143199926	143250600	LINC00960,FRG2C	NA,TRN-GTT7-1,TRN-GTT11-2
chr5	49600099	49649612	chr1	125150424	125184388		NA,TRN-GTT7-1,TRN-GTT11-2
chr5	49600099	49649612	chr1	143199926	143250600		NA,TRN-GTT7-1,TRN-GTT11-2
chr5	49600099	49649612	chr1	143250597	143299572		NA,TRN-GTT7-1,TRN-GTT11-2
chr5	1	51038	chr4	190100265	190173349		
chr7	62300512	62349945	chr1	143199926	143250600		NA,TRN-GTT7-1,TRN-GTT11-2
chr7	76800089	76850042	chr1	83350033	83400125		NA
chr9	64100191	64134903	chr4	49199782	49251604		
chrY	11296644	11349949	chr1	125150424	125184388		NA,TRN-GTT7-1,TRN-GTT11-2
chrY	11296644	11349949	chr1	143199926	143250600		NA,TRN-GTT7-1,TRN-GTT11-2
chrY	11296644	11349949	chr1	143250597	143299572		NA,TRN-GTT7-1,TRN-GTT11-2
chrY	56699637	56749635	chr1	143199926	143250600		NA,TRN-GTT7-1,TRN-GTT11-2
chrY	56749632	56823171	chr1	143199926	143250600		NA,TRN-GTT7-1,TRN-GTT11-2
chrY	56823168	56850027	chr1	125150424	125184388		NA,TRN-GTT7-1,TRN-GTT11-2
chrY	56823168	56850027	chr1	143199926	143250600		NA,TRN-GTT7-1,TRN-GTT11-2
chrY	11296644	11349949	chr10	42066912	42099793		
chrY	56850024	56900380	chr10	125850030	125900082		DHX32,BCCIP,FANK1
chrY	56850024	56900380	chr10	125900079	125949982		FANK1
chrY	56699637	56749635	chr13	18200335	18249767		FAM230C
chrY	56749632	56823171	chr13	18200335	18249767		FAM230C
chrY	56850024	56900380	chr13	18300237	18349910		
chrY	10099761	10150337	chr16	46381705	46400141		

chrY	10150334	10200172	chr16	34571526	34599812	
chrY	10150334	10200172	chr16	46381705	46400141	
chrY	10649776	10694177	chr16	34571526	34599812	
chrY	10649776	10694177	chr16	46381705	46400141	
chrY	10649776	10694177	chr16	46400138	46449969	
chrY	10749748	10800130	chr16	34571526	34599812	
chrY	10749748	10800130	chr16	46381705	46400141	
chrY	10749748	10800130	chr16	46400138	46449969	
chrY	10800127	10849884	chr16	34571526	34599812	
chrY	10800127	10849884	chr16	46381705	46400141	
chrY	10900040	10949855	chr16	34571526	34599812	
chrY	10949852	11001474	chr16	34571526	34599812	
chrY	10949852	11001474	chr16	46381705	46400141	
chrY	11249766	11296647	chr16	34571526	34599812	
chrY	11249766	11296647	chr16	46381705	46400141	
chrY	11296644	11349949	chr16	34571526	34599812	
chrY	11296644	11349949	chr16	46381705	46400141	
chrY	11296644	11349949	chr16	46400138	46449969	
chrY	56699637	56749635	chr16	34571526	34599812	
chrY	56699637	56749635	chr16	46381705	46400141	
chrY	56699637	56749635	chr16	46400138	46449969	
chrY	56749632	56823171	chr16	34571526	34599812	
chrY	56749632	56823171	chr16	46381705	46400141	
chrY	56823168	56850027	chr16	33699797	33750323	LOC102724207
chrY	56823168	56850027	chr16	34049728	34099513	
chrY	56823168	56850027	chr16	34571526	34599812	
chrY	56823168	56850027	chr16	46381705	46400141	
chrY	56823168	56850027	chr16	46400138	46449969	
chrY	56850024	56900380	chr16	34571526	34599812	
chrY	56850024	56900380	chr16	46381705	46400141	
chrY	56850024	56900380	chr16	46400138	46449969	
chrY	11296644	11349949	chr18	100130	149971	ROCK1P1,MIR8078

chrY	56823168	56850027	chr18	100130	149971	ROCK1P1,MIR8078
chrY	56823168	56850027	chr2	88750494	88799987	RPIA,ANKRD36BP2
chrY	56823168	56850027	chr2	95899859	95949911	ANKRD36C
chrY	56823168	56850027	chr2	95949908	96000075	ANKRD36C
chrY	56823168	56850027	chr2	97149974	97199975	ANKRD36
chrY	56850024	56900380	chr2	95899859	95949911	ANKRD36C
chrY	11100271	11149871	chr21	8999951	9050237	LOC105372731
chrY	11249766	11296647	chr21	10399951	10449840	BAGE2
chrY	11296644	11349949	chr21	10350224	10399954	
chrY	11296644	11349949	chr21	10399951	10449840	BAGE2
chrY	11296644	11349949	chr21	10449837	10500162	BAGE2,LOC105378260
chrY	56699637	56749635	chr21	10270164	10324331	
chrY	56699637	56749635	chr21	10324328	10350227	
chrY	56749632	56823171	chr21	10270164	10324331	
chrY	56749632	56823171	chr21	10324328	10350227	
chrY	56823168	56850027	chr21	9749959	9800076	LINC01667
chrY	56823168	56850027	chr21	10350224	10399954	
chrY	56823168	56850027	chr21	10399951	10449840	BAGE2
chrY	56823168	56850027	chr21	10449837	10500162	BAGE2,LOC105378260
chrY	56823168	56850027	chr21	10750041	10799904	
chrY	56850024	56900380	chr21	5216622	5249633	
chrY	56850024	56900380	chr21	9749959	9800076	LINC01667
chrY	56850024	56900380	chr21	10350224	10399954	
chrY	56850024	56900380	chr21	10399951	10449840	BAGE2
chrY	56850024	56900380	chr21	10449837	10500162	BAGE2,LOC105378260
chrY	56850024	56900380	chr21	10750041	10799904	
chrY	11296644	11349949	chr22	12641507	12700005	
chrY	56699637	56749635	chr22	18709576	18750410	LINC01662
chrY	56699637	56749635	chr22	18850026	18899542	
chrY	56749632	56823171	chr22	18709576	18750410	LINC01662
chrY	56749632	56823171	chr22	18850026	18899542	
chrY	56823168	56850027	chr22	10700993	10750376	

chrY	56823168	56850027	chr22	10750373	10784643	
chrY	56823168	56850027	chr22	11016725	11050158	
chrY	56850024	56900380	chr22	10700993	10750376	
chrY	56850024	56900380	chr22	10750373	10784643	
chrY	56850024	56900380	chr22	11016725	11050158	
chrY	56850024	56900380	chr22	11050155	11118991	
chrY	56850024	56900380	chr22	11549683	11600131	
chrY	11296644	11349949	chr3	75649945	75700347	LINC00960,FRG2C
chrY	11296644	11349949	chr4	49147262	49199785	
chrY	11296644	11349949	chr4	49499867	49550044	
chrY	11296644	11349949	chr4	49599978	49650186	
chrY	56823168	56850027	chr4	49499867	49550044	
chrY	56823168	56850027	chr4	49599978	49650186	
chrY	56850024	56900380	chr4	49499867	49550044	
chrY	56850024	56900380	chr4	49599978	49650186	
chrY	11296644	11349949	chr5	49600099	49649612	
chrY	56850024	56900380	chr7	152400017	152450104	KMT2C,FABP5P3

Supplemental Table 1: Detected transchromosomal interactions in mouse B cells							
Chromosome	Start	End	Chromosome	Start	End	Gene associated with first anchor	Gene associated with second anchor
chr10	22099860	22149739	chr1	183249934	183299851	E030030I06Rik	Brox,Aida
chr10	22099860	22149739	chr1	195200096	195249561	E030030I06Rik	
chr10	22099860	22149739	chr2	181899774	181950034	E030030I06Rik	
chr10	22099860	22149739	chr3	8199969	8250010	E030030I06Rik	
chr10	22099860	22149739	chr4	3050773	3099791	E030030I06Rik	
chr10	11450721	11499362	chr5	146250715	146300005	Epm2a	Cdk8,NA
chr10	22099860	22149739	chr5	146250715	146300005	E030030I06Rik	Cdk8,NA
chr11	3149972	3199980	chr1	88200133	88249820	Sfi1	Dnajb3,Ugt1a2,Ugt1a6a,Ugt1a10,Ugt1a7c,Ugt1a5,Ugt1a9,Ugt1a6b,Ugt1a1,Mroh2a
chr11	3149972	3199980	chr1	88249817	88299855	Sfi1	6430706D22Rik,Hjurp,Mroh2a
chr11	3149972	3199980	chr10	22099860	22149739	Sfi1	E030030I06Rik
chr11	54100052	54150476	chr10	22099860	22149739	P4ha2	E030030I06Rik
chr11	108999946	109049739	chr10	22099860	22149739		E030030I06Rik
chr11	3102115	3149975	chr2	3050197	3099735	Sfi1,Pisd-ps1	
chr11	3149972	3199980	chr2	98649599	98700076	Sfi1	
chr11	3149972	3199980	chr2	181899774	181950034	Sfi1	
chr11	54100052	54150476	chr2	181899774	181950034	P4ha2	
chr11	108999946	109049739	chr2	181899774	181950034		
chr11	3149972	3199980	chr3	8199969	8250010	Sfi1	
chr11	54100052	54150476	chr3	8199969	8250010	P4ha2	
chr11	108999946	109049739	chr3	5849735	5900077		
chr11	108999946	109049739	chr3	8199969	8250010		
chr11	3149972	3199980	chr4	3050773	3099791	Sfi1	
chr11	3149972	3199980	chr4	3199973	3249853	Sfi1	
chr11	3149972	3199980	chr4	141100135	141149689	Sfi1	Szrd1,Fbxo42,Spata21
chr11	108999946	109049739	chr4	3050773	3099791		
chr11	3149972	3199980	chr5	135350081	135400045	Sfi1	Nsun5,Pom121,Trim50
chr11	3149972	3199980	chr5	146250715	146300005	Sfi1	Cdk8,NA
chr11	54100052	54150476	chr5	146250715	146300005	P4ha2	Cdk8,NA
chr11	108999946	109049739	chr5	146250715	146300005		Cdk8,NA
chr11	3149972	3199980	chr6	3199752	3250020	Sfi1	Rn18s
chr11	3149972	3199980	chr6	103599835	103649972	Sfi1	Chl1
chr11	3149972	3199980	chr9	3000001	3049479	Sfi1	Mir101c
chr11	3149972	3199980	chr9	6350052	6400267	Sfi1	Pdgfd
chr11	3149972	3199980	chr9	35299842	35350067	Sfi1	
chr12	51450299	51499933	chr1	88249817	88299855		6430706D22Rik,Hjurp,Mroh2a
chr12	67050146	67099887	chr11	3149972	3199980	Mdga2	Sfi1
chr12	20200108	20249248	chr4	146149438	146200078	NA	Zfp600
chr12	20200108	20249248	chr4	146450134	146499188	NA	Zfp992
chr12	20200108	20249248	chr4	146499185	146549996	NA	1700095A21Rik,Zfp981

chr12	20200108	20249248	chr4	146708801	146750182	NA		NA,Gm20875
chr12	20200108	20249248	chr4	147299595	147350006	NA		
chr12	20200108	20249248	chr4	147400251	147449702	NA		
chr12	3000001	3049529	chr5	3000001	3050004			V1rg10
chr13	3350341	3399887	chr1	183249934	183299851	NA		Brox,Aida
chr13	3350341	3399887	chr10	22099860	22149739	NA		E030030I06Rik
chr13	9000057	9050074	chr10	22099860	22149739			E030030I06Rik
chr13	99749094	99800064	chr10	22099860	22149739			E030030I06Rik
chr13	3350341	3399887	chr11	3149972	3199980	NA		Sfi1
chr13	3350341	3399887	chr11	54100052	54150476	NA		P4ha2
chr13	3350341	3399887	chr11	108999946	109049739	NA		
chr13	3350341	3399887	chr2	181899774	181950034	NA		
chr13	9000057	9050074	chr2	181899774	181950034			
chr13	44850383	44900261	chr2	181899774	181950034	Jarid2		
chr13	99749094	99800064	chr2	181899774	181950034			
chr13	3350341	3399887	chr3	8199969	8250010	NA		
chr13	9000057	9050074	chr3	8199969	8250010			
chr13	3000480	3050284	chr4	3199973	3249853			
chr13	3350341	3399887	chr4	3050773	3099791	NA		
chr13	3350341	3399887	chr5	146250715	146300005	NA		Cdk8,NA
chr13	9000057	9050074	chr5	146250715	146300005			Cdk8,NA
chr13	3000480	3050284	chr6	3149518	3199755			Rn18s
chr13	119595537	119599781	chr9	124250486	124300017	Tmem267		2010315B03Rik
chr13	119599778	119649908	chr9	124250486	124300017	Ccl28,Tmem267		2010315B03Rik
chr14	19350468	19399525	chr11	3149972	3199980			Sfi1
chr14	19399522	19469708	chr11	3149972	3199980			Sfi1
chr14	19399522	19469708	chr9	3000001	3049479			Mir101c
chr15	75049941	75099970	chr1	183249934	183299851			Brox,Aida
chr15	75049941	75099970	chr1	195200096	195249561			
chr15	75049941	75099970	chr10	22099860	22149739			E030030I06Rik
chr15	75049941	75099970	chr11	3149972	3199980			Sfi1
chr15	75049941	75099970	chr11	54100052	54150476			P4ha2
chr15	75049941	75099970	chr11	108999946	109049739			
chr15	103249683	103299695	chr11	54100052	54150476	Nfe2,Copz1,Mir148b,NA		P4ha2
chr15	75049941	75099970	chr13	3350341	3399887			NA
chr15	75049941	75099970	chr13	9000057	9050074			
chr15	75049941	75099970	chr13	99749094	99800064			
chr15	75049941	75099970	chr2	181899774	181950034			
chr15	75049941	75099970	chr3	8199969	8250010			
chr15	75049941	75099970	chr3	152699887	152749949			Pigk
chr15	3050001	3100097	chr4	3199973	3249853			

chr15	3050001	3100097	chr4	3249850	3299716		NA
chr15	75049941	75099970	chr4	3050773	3099791		
chr15	75049941	75099970	chr5	146250715	146300005		Cdk8,NA
chr15	75049941	75099970	chr6	3199752	3250020		Rn18s
chr15	75049941	75099970	chr9	123449722	123500196		Limd1,Lars2
chr16	3100168	3149912	chr10	22099860	22149739		E030030I06Rik
chr16	3350440	3400083	chr10	22099860	22149739		E030030I06Rik
chr16	3100168	3149912	chr11	54100052	54150476		P4ha2
chr16	3181571	3250185	chr11	3149972	3199980		Sfi1
chr16	3250182	3300027	chr11	3149972	3199980		Sfi1
chr16	3100168	3149912	chr13	3350341	3399887		NA
chr16	3181571	3250185	chr13	3350341	3399887		NA
chr16	3100168	3149912	chr15	75049941	75099970		
chr16	3350440	3400083	chr15	75049941	75099970		
chr16	11100294	11150718	chr15	75049941	75099970	Txndc11,Zc3h7a	
chr16	17199671	17250186	chr15	75049941	75099970	Ube2l3,Hic2,Rimbp3	
chr16	57350249	57400043	chr15	75049941	75099970	Cmss1,Filip1l	
chr16	3100168	3149912	chr2	181899774	181950034		
chr16	3350440	3400083	chr2	181899774	181950034		
chr16	11100294	11150718	chr2	181899774	181950034	Txndc11,Zc3h7a	
chr16	17199671	17250186	chr2	181899774	181950034	Ube2l3,Hic2,Rimbp3	
chr16	57350249	57400043	chr2	181899774	181950034	Cmss1,Filip1l	
chr16	91499244	91549980	chr2	181899774	181950034	Ifnar1,Ifngr2	
chr16	3100168	3149912	chr3	8199969	8250010		
chr16	57350249	57400043	chr3	8199969	8250010	Cmss1,Filip1l	
chr16	3181571	3250185	chr4	3050773	3099791		
chr16	3181571	3250185	chr5	79399974	79449996		
chr16	3250182	3300027	chr5	79399974	79449996		
chr16	57350249	57400043	chr5	146250715	146300005	Cmss1,Filip1l	Cdk8,NA
chr17	3000418	3050005	chr1	88200133	88249820		Dnajb3,Ugt1a2,Ugt1a6a,Ugt1a10,Ugt1a7c,Ugt1a5,Ugt1a9,Ugt1a6b,Ugt1a1,Mroh2a
chr17	3000418	3050005	chr1	88249817	88299855		6430706D22Rik,Hjurp,Mroh2a
chr17	23300132	23349848	chr1	88200133	88249820	Vmn2r115,Vmn2r114	Dnajb3,Ugt1a2,Ugt1a6a,Ugt1a10,Ugt1a7c,Ugt1a5,Ugt1a9,Ugt1a6b,Ugt1a1,Mroh2a
chr17	23300132	23349848	chr1	88249817	88299855	Vmn2r115,Vmn2r114	6430706D22Rik,Hjurp,Mroh2a
chr17	23450579	23499746	chr1	88200133	88249820	Vmn2r117	Dnajb3,Ugt1a2,Ugt1a6a,Ugt1a10,Ugt1a7c,Ugt1a5,Ugt1a9,Ugt1a6b,Ugt1a1,Mroh2a
chr17	23499743	23549799	chr1	88200133	88249820		Dnajb3,Ugt1a2,Ugt1a6a,Ugt1a10,Ugt1a7c,Ugt1a5,Ugt1a9,Ugt1a6b,Ugt1a1,Mroh2a
chr17	23499743	23549799	chr1	88249817	88299855		6430706D22Rik,Hjurp,Mroh2a
chr17	39800075	39850047	chr1	58599814	58650138	Rn45s	
chr17	39800075	39850047	chr1	102600222	102650516	Rn45s	
chr17	39800075	39850047	chr1	112449969	112500029	Rn45s	
chr17	39800075	39850047	chr1	183249934	183299851	Rn45s	Brox,Aida
chr17	39800075	39850047	chr1	195200096	195249561	Rn45s	

chr17	39800075	39850047	chr10	11450721	11499362	Rn45s	Epm2a
chr17	39800075	39850047	chr10	22099860	22149739	Rn45s	E030030I06Rik
chr17	39800075	39850047	chr10	70199844	70250250	Rn45s	Slc16a9,Mrln
chr17	3000418	3050005	chr11	3149972	3199980		Sfi1
chr17	39800075	39850047	chr11	3149972	3199980	Rn45s	Sfi1
chr17	39800075	39850047	chr11	54100052	54150476	Rn45s	P4ha2
chr17	39800075	39850047	chr11	106049959	106099962	Rn45s	Map3k3,Taco1,Dcaf7
chr17	39800075	39850047	chr11	108999946	109049739	Rn45s	
chr17	39800075	39850047	chr12	3349958	3399929	Rn45s	Kif3c
chr17	39800075	39850047	chr12	78350565	78400123	Rn45s	Gphn
chr17	39800075	39850047	chr13	3350341	3399887	Rn45s	NA
chr17	39800075	39850047	chr13	9000057	9050074	Rn45s	
chr17	39800075	39850047	chr13	44850383	44900261	Rn45s	Jarid2
chr17	39800075	39850047	chr13	99749094	99800064	Rn45s	
chr17	39800075	39850047	chr14	37850224	37900301	Rn45s	
chr17	36199879	36250086	chr15	75049941	75099970	Gm10074	
chr17	39800075	39850047	chr15	3249844	3300320	Rn45s	Selenop,Ccdc152
chr17	39800075	39850047	chr15	3649948	3700109	Rn45s	
chr17	39800075	39850047	chr15	75049941	75099970	Rn45s	
chr17	39800075	39850047	chr16	3100168	3149912	Rn45s	
chr17	39800075	39850047	chr16	3350440	3400083	Rn45s	
chr17	39800075	39850047	chr16	11100294	11150718	Rn45s	Txndc11,Zc3h7a
chr17	39800075	39850047	chr16	11499749	11549892	Rn45s	Snx29
chr17	39800075	39850047	chr16	17199671	17250186	Rn45s	Ube2l3,Hic2,Rimbp3
chr17	39800075	39850047	chr16	57350249	57400043	Rn45s	Cmss1,Filip1l
chr17	39800075	39850047	chr16	91499244	91549980	Rn45s	Ifnar1,Ifngr2
chr17	36199879	36250086	chr2	181899774	181950034	Gm10074	
chr17	39800075	39850047	chr2	22699938	22750211	Rn45s	
chr17	39800075	39850047	chr2	98649599	98700076	Rn45s	
chr17	39800075	39850047	chr2	102800197	102850133	Rn45s	Cd44
chr17	39800075	39850047	chr2	181899774	181950034	Rn45s	
chr17	56000102	56050299	chr2	181899774	181950034	Sh3gl1,Chaf1a,Mpnd,Stap2	
chr17	36199879	36250086	chr3	8199969	8250010	Gm10074	
chr17	39800075	39850047	chr3	5849735	5900077	Rn45s	
chr17	39800075	39850047	chr3	8199969	8250010	Rn45s	
chr17	39800075	39850047	chr3	63500060	63550836	Rn45s	
chr17	39800075	39850047	chr3	152699887	152749949	Rn45s	Pigk
chr17	3000418	3050005	chr4	3050773	3099791		
chr17	39800075	39850047	chr4	3050773	3099791	Rn45s	
chr17	39800075	39850047	chr4	34900007	34950368	Rn45s	Cga,Platr9,Mob3b
chr17	36199879	36250086	chr5	146250715	146300005	Gm10074	Cdk8,NA

chr17	39800075	39850047	chr5	109550198	109599891	Rn45s	Crlf2
chr17	39800075	39850047	chr5	146250715	146300005	Rn45s	Cdk8,NA
chr17	39800075	39850047	chr6	3199752	3250020	Rn45s	Rn18s
chr17	39800075	39850047	chr6	49199895	49250056	Rn45s	Tra2a,Igf2bp3
chr17	39800075	39850047	chr6	103599835	103649972	Rn45s	Chl1
chr17	39800075	39850047	chr7	128699982	128750169	Rn45s	Sec23ip,Mcmbp
chr17	39800075	39850047	chr7	130800084	130849819	Rn45s	Btbd16,NA
chr17	39800075	39850047	chr8	3750280	3800042	Rn45s	
chr17	39800075	39850047	chr8	15500197	15549866	Rn45s	
chr17	39800075	39850047	chr8	125750083	125800175	Rn45s	Pcnx2
chr17	39800075	39850047	chr9	3000001	3049479	Rn45s	Mir101c
chr17	39800075	39850047	chr9	110250011	110300485	Rn45s	Cspg5
chr17	39800075	39850047	chr9	123449722	123500196	Rn45s	Limd1,Lars2
chr18	3001227	3050237	chr1	195200096	195249561		
chr18	3001227	3050237	chr10	22099860	22149739		E030030I06Rik
chr18	40299987	40350143	chr10	22099860	22149739	Kctd16	E030030I06Rik
chr18	3001227	3050237	chr11	3149972	3199980		Sfi1
chr18	3001227	3050237	chr11	54100052	54150476		P4ha2
chr18	3001227	3050237	chr11	108999946	109049739		
chr18	40299987	40350143	chr11	3149972	3199980	Kctd16	Sfi1
chr18	40299987	40350143	chr11	54100052	54150476	Kctd16	P4ha2
chr18	3001227	3050237	chr13	3350341	3399887		NA
chr18	3001227	3050237	chr13	9000057	9050074		
chr18	3001227	3050237	chr13	99749094	99800064		
chr18	40299987	40350143	chr13	3350341	3399887	Kctd16	NA
chr18	3001227	3050237	chr15	75049941	75099970		
chr18	40299987	40350143	chr15	75049941	75099970	Kctd16	
chr18	3001227	3050237	chr16	3100168	3149912		
chr18	3001227	3050237	chr16	3181571	3250185		
chr18	3001227	3050237	chr16	3250182	3300027		
chr18	3001227	3050237	chr16	11100294	11150718		Txndc11,Zc3h7a
chr18	3001227	3050237	chr16	57350249	57400043		Cmss1,Filip1l
chr18	3050234	3099928	chr16	3250182	3300027		
chr18	3001227	3050237	chr17	36199879	36250086		Gm10074
chr18	3001227	3050237	chr17	39800075	39850047		Rn45s
chr18	3300451	3350460	chr17	39800075	39850047	Crem,Gm6225	Rn45s
chr18	12900001	12949796	chr17	39800075	39850047	Osbpl1a	Rn45s
chr18	14099959	14150138	chr17	39800075	39850047		Rn45s
chr18	40299987	40350143	chr17	39800075	39850047	Kctd16	Rn45s
chr18	68649747	68699965	chr17	39800075	39850047		Rn45s
chr18	85650030	85699893	chr17	39800075	39850047		Rn45s

chr18	3001227	3050237	chr2	181899774	181950034		
chr18	40299987	40350143	chr2	181899774	181950034	Kctd16	
chr18	68649747	68699965	chr2	181899774	181950034		
chr18	3001227	3050237	chr3	8199969	8250010		
chr18	40299987	40350143	chr3	8199969	8250010	Kctd16	
chr18	3001227	3050237	chr4	3050773	3099791		
chr18	3001227	3050237	chr5	146250715	146300005		Cdk8,NA
chr18	3001227	3050237	chr6	3199752	3250020		Rn18s
chr18	3001227	3050237	chr9	123449722	123500196		Limd1,Lars2
chr19	13650019	13699948	chr11	3149972	3199980	Olfr1490	Sfi1
chr19	19200407	19249814	chr11	3149972	3199980		Sfi1
chr19	19249811	19299644	chr11	3149972	3199980		Sfi1
chr19	19449922	19500127	chr11	3149972	3199980		Sfi1
chr19	22749586	22799508	chr11	3149972	3199980	Trpm3,Mir204	Sfi1
chr19	45649879	45699993	chr15	75049941	75099970	Fbxw4	
chr19	61149845	61200309	chr15	75049941	75099970	Gm7102	
chr19	61250119	61299557	chr15	75049941	75099970		
chr19	3150425	3200160	chr16	3250182	3300027	1700030N03Rik	
chr19	30400075	30450077	chr17	39800075	39850047		Rn45s
chr19	45649879	45699993	chr17	39800075	39850047	Fbxw4	Rn45s
chr19	61149845	61200309	chr17	39800075	39850047	Gm7102	Rn45s
chr19	61250119	61299557	chr17	39800075	39850047		Rn45s
chr19	45649879	45699993	chr18	3001227	3050237	Fbxw4	
chr19	45649879	45699993	chr18	40299987	40350143	Fbxw4	Kctd16
chr19	61149845	61200309	chr18	3001227	3050237	Gm7102	
chr19	61250119	61299557	chr18	3001227	3050237		
chr19	45649879	45699993	chr2	181899774	181950034	Fbxw4	
chr19	61149845	61200309	chr2	181899774	181950034	Gm7102	
chr19	61200306	61250122	chr2	181899774	181950034	Csf2ra,Gm4242	
chr19	61250119	61299557	chr2	181899774	181950034		
chr19	45649879	45699993	chr3	8199969	8250010	Fbxw4	
chr2	3150025	3200385	chr1	88200133	88249820	Fam171a1	Dnajb3,Ugt1a2,Ugt1a6a,Ugt1a10,Ugt1a7c,Ugt1a5,Ugt1a9,Ugt1a6b,Ugt1a1,Mroh2a
chr2	3150025	3200385	chr1	88249817	88299855	Fam171a1	6430706D22Rik,Hjurp,Mroh2a
chr2	4349880	4399984	chr1	88200133	88249820	Frmd4a	Dnajb3,Ugt1a2,Ugt1a6a,Ugt1a10,Ugt1a7c,Ugt1a5,Ugt1a9,Ugt1a6b,Ugt1a1,Mroh2a
chr2	181899774	181950034	chr1	58599814	58650138		
chr2	181899774	181950034	chr1	183249934	183299851		Brox,Aida
chr2	181899774	181950034	chr1	195200096	195249561		
chr3	3000788	3050004	chr1	88200133	88249820		Dnajb3,Ugt1a2,Ugt1a6a,Ugt1a10,Ugt1a7c,Ugt1a5,Ugt1a9,Ugt1a6b,Ugt1a1,Mroh2a
chr3	3000788	3050004	chr1	88249817	88299855		6430706D22Rik,Hjurp,Mroh2a
chr3	3050001	3099957	chr1	88200133	88249820		Dnajb3,Ugt1a2,Ugt1a6a,Ugt1a10,Ugt1a7c,Ugt1a5,Ugt1a9,Ugt1a6b,Ugt1a1,Mroh2a
chr3	3050001	3099957	chr1	88249817	88299855		6430706D22Rik,Hjurp,Mroh2a

chr3	3099954	3149828	chr1	88249817	88299855		6430706D22Rik,Hjurp,Mroh2a
chr3	3200068	3249959	chr1	88200133	88249820		Dnajb3,Ugt1a2,Ugt1a6a,Ugt1a10,Ugt1a7c,Ugt1a5,Ugt1a9,Ugt1a6b,Ugt1a1,Mroh2a
chr3	3200068	3249959	chr1	88249817	88299855		6430706D22Rik,Hjurp,Mroh2a
chr3	5849735	5900077	chr2	181899774	181950034		
chr3	8199969	8250010	chr2	181899774	181950034		
chr3	63500060	63550836	chr2	181899774	181950034		
chr3	152699887	152749949	chr2	181899774	181950034	Pigk	
chr4	3050773	3099791	chr1	88200133	88249820		Dnajb3,Ugt1a2,Ugt1a6a,Ugt1a10,Ugt1a7c,Ugt1a5,Ugt1a9,Ugt1a6b,Ugt1a1,Mroh2a
chr4	3050773	3099791	chr1	88249817	88299855		6430706D22Rik,Hjurp,Mroh2a
chr4	3050773	3099791	chr2	181899774	181950034		
chr4	3050773	3099791	chr3	8199969	8250010		
chr4	34900007	34950368	chr3	8199969	8250010	Cga,Platr9,Mob3b	
chr5	146250715	146300005	chr2	181899774	181950034	Cdk8,NA	
chr5	146250715	146300005	chr3	8199969	8250010	Cdk8,NA	
chr5	77400011	77449890	chr4	3199973	3249853	Igfbp7	
chr5	146250715	146300005	chr4	3050773	3099791	Cdk8,NA	
chr6	103599835	103649972	chr1	88200133	88249820	Chl1	Dnajb3,Ugt1a2,Ugt1a6a,Ugt1a10,Ugt1a7c,Ugt1a5,Ugt1a9,Ugt1a6b,Ugt1a1,Mroh2a
chr6	3199752	3250020	chr2	181899774	181950034	Rn18s	
chr6	49199895	49250056	chr2	181899774	181950034	Tra2a,Igf2bp3	
chr6	103599835	103649972	chr2	98649599	98700076	Chl1	
chr6	3050001	3099781	chr4	3050773	3099791		
chr6	3149518	3199755	chr4	3199973	3249853	Rn18s	
chr6	3349828	3399887	chr4	3050773	3099791	Samd9l	
chr6	3399884	3449758	chr4	3050773	3099791		
chr6	3449755	3499596	chr4	3050773	3099791	Vps50,Hepacam2	
chr6	58550148	58600110	chr4	147400251	147449702	Abcg2	
chr6	58600107	58650375	chr4	147400251	147449702	Abcg2	
chr6	67649862	67699998	chr4	146450134	146499188		Zfp992
chr6	67649862	67699998	chr4	147400251	147449702		
chr7	128699982	128750169	chr2	181899774	181950034	Sec23ip,Mcmbp	
chr8	15500197	15549866	chr2	181899774	181950034		
chr9	3300202	3349883	chr1	88200133	88249820	Alkbh8	Dnajb3,Ugt1a2,Ugt1a6a,Ugt1a10,Ugt1a7c,Ugt1a5,Ugt1a9,Ugt1a6b,Ugt1a1,Mroh2a
chr9	3300202	3349883	chr1	88249817	88299855	Alkbh8	6430706D22Rik,Hjurp,Mroh2a
chr9	3000001	3049479	chr2	98649599	98700076	Mir101c	
chr9	24501162	24550023	chr2	98649599	98700076	Dpy19l1	
chr9	123449722	123500196	chr2	181899774	181950034	Limd1,Lars2	
chr9	24501162	24550023	chr6	103599835	103649972	Dpy19l1	Chl1
chrX	143450048	143500816	chr11	3149972	3199980		Sfi1
chrX	4849977	4899691	chr17	39800075	39850047		Rn45s
chrX	87199968	87250042	chr17	39800075	39850047	Il1rapl1	Rn45s
chrX	143450048	143500816	chr2	98649599	98700076		

chrX	124100008	124149871	chr4	156306042	156349983	Vmn2r121	Vmn2r-ps159,Vmn2r125
chrX	143450048	143500816	chr6	103599835	103649972		Chl1
chrX	143450048	143500816	chr9	24501162	24550023		Dpy19l1
chrY	90699748	90750101	chr13	119599778	119649908	NA	Ccl28,Tmem267
chrY	90699748	90750101	chrX	169949960	169999781	NA	Mid1,G530011O06Rik

Supplemental Table 1: Detected transchromosomal interactions in mouse CD4+ T cells							
Chromosome	Start	End	Chromosome	Start	End	Gene associated with first anchor	Gene associated with second anchor
chr10	22099860	22149739	chr1	183249934	183299851	E030030I06Rik	Brox,Aida
chr10	22099860	22149739	chr2	181899774	181950034	E030030I06Rik	
chr10	22099860	22149739	chr3	8199969	8250010	E030030I06Rik	
chr10	22099860	22149739	chr4	3050773	3099791	E030030I06Rik	
chr10	11450721	11499362	chr5	146250715	146300005	Epm2a	Cdk8,NA
chr10	22099860	22149739	chr9	123449722	123500196	E030030I06Rik	Limd1,Lars2
chr11	3149972	3199980	chr1	88200133	88249820	Sfi1	Dnajb3,Ugt1a2,Ugt1a6a,Ugt1a10,Ugt1a7c,Ugt1a5,Ugt1a9,Ugt1a6b,Ugt1a1,Mroh2a
chr11	3149972	3199980	chr1	88249817	88299855	Sfi1	6430706D22Rik,Hjurp,Mroh2a
chr11	3149972	3199980	chr10	22099860	22149739	Sfi1	E030030I06Rik
chr11	54100052	54150476	chr10	22099860	22149739	P4ha2	E030030I06Rik
chr11	108999946	109049739	chr10	22099860	22149739		E030030I06Rik
chr11	3102115	3149975	chr2	3050197	3099735	Sfi1,Pisd-ps1	
chr11	3149972	3199980	chr2	98649599	98700076	Sfi1	
chr11	3149972	3199980	chr2	181899774	181950034	Sfi1	
chr11	54100052	54150476	chr2	181899774	181950034	P4ha2	
chr11	108999946	109049739	chr2	181899774	181950034		
chr11	3149972	3199980	chr3	8199969	8250010	Sfi1	
chr11	54100052	54150476	chr3	8199969	8250010	P4ha2	
chr11	108999946	109049739	chr3	8199969	8250010		
chr11	3149972	3199980	chr4	3050773	3099791	Sfi1	
chr11	3149972	3199980	chr4	3199973	3249853	Sfi1	
chr11	3149972	3199980	chr4	141100135	141149689	Sfi1	Szrd1,Fbxo42,Spata21
chr11	54100052	54150476	chr4	3050773	3099791	P4ha2	
chr11	108999946	109049739	chr4	3050773	3099791		
chr11	3149972	3199980	chr5	135350081	135400045	Sfi1	Nsun5,Pom121,Trim50
chr11	108999946	109049739	chr5	146250715	146300005		Cdk8,NA
chr11	3149972	3199980	chr6	103599835	103649972	Sfi1	Chl1
chr11	3149972	3199980	chr9	3000001	3049479	Sfi1	Mir101c
chr11	3149972	3199980	chr9	35299842	35350067	Sfi1	
chr11	108999946	109049739	chr9	123449722	123500196		Limd1,Lars2
chr12	20200108	20249248	chr4	146450134	146499188	NA	Zfp992
chr12	20200108	20249248	chr4	147299595	147350006	NA	
chr12	20200108	20249248	chr4	147400251	147449702	NA	
chr12	20200108	20249248	chr4	147750002	147800228	NA	Zfp984
chr13	3350341	3399887	chr1	183249934	183299851	NA	Brox,Aida
chr13	3350341	3399887	chr1	195200096	195249561	NA	
chr13	3350341	3399887	chr10	22099860	22149739	NA	E030030I06Rik
chr13	9000057	9050074	chr10	22099860	22149739		E030030I06Rik

chr13	99749094	99800064	chr10	22099860	22149739		E030030I06Rik
chr13	3350341	3399887	chr11	3149972	3199980	NA	Sfi1
chr13	3350341	3399887	chr11	54100052	54150476	NA	P4ha2
chr13	3350341	3399887	chr11	108999946	109049739	NA	
chr13	12900033	12949926	chr11	3149972	3199980		Sfi1
chr13	3350341	3399887	chr2	181899774	181950034	NA	
chr13	9000057	9050074	chr2	181899774	181950034		
chr13	44850383	44900261	chr2	181899774	181950034	Jarid2	
chr13	99749094	99800064	chr2	181899774	181950034		
chr13	3350341	3399887	chr3	8199969	8250010	NA	
chr13	9000057	9050074	chr3	8199969	8250010		
chr13	3000480	3050284	chr4	3199973	3249853		
chr13	3350341	3399887	chr4	3050773	3099791	NA	
chr13	3350341	3399887	chr5	146250715	146300005	NA	Cdk8,NA
chr13	3000480	3050284	chr6	3149518	3199755		Rn18s
chr13	3350341	3399887	chr9	123449722	123500196	NA	Limd1,Lars2
chr13	119595537	119599781	chr9	123949939	123999999	Tmem267	Ccr1,Ccr1l1
chr13	119595537	119599781	chr9	124250486	124300017	Tmem267	2010315B03Rik
chr13	119599778	119649908	chr9	124250486	124300017	Ccl28,Tmem267	2010315B03Rik
chr13	119599778	119649908	chr9	124300014	124349913	Ccl28,Tmem267	Nlrp4g,2010315B03Rik,NA
chr14	19399522	19469708	chr11	3149972	3199980		Sfi1
chr14	19399522	19469708	chr9	3000001	3049479		Mir101c
chr15	75049941	75099970	chr1	195200096	195249561		
chr15	75049941	75099970	chr10	22099860	22149739		E030030I06Rik
chr15	3599593	3649951	chr11	3149972	3199980		Sfi1
chr15	75049941	75099970	chr11	3149972	3199980		Sfi1
chr15	75049941	75099970	chr11	54100052	54150476		P4ha2
chr15	75049941	75099970	chr11	108999946	109049739		
chr15	75049941	75099970	chr13	3350341	3399887		NA
chr15	75049941	75099970	chr13	9000057	9050074		
chr15	75049941	75099970	chr13	99749094	99800064		
chr15	3149859	3199885	chr2	181899774	181950034		
chr15	75049941	75099970	chr2	181899774	181950034		
chr15	75049941	75099970	chr3	8199969	8250010		
chr15	75049941	75099970	chr3	152699887	152749949		Pigk
chr15	103249683	103299695	chr3	8199969	8250010	Nfe2,Copz1,Mir148b,NA	
chr15	3050001	3100097	chr4	3199973	3249853		
chr15	3050001	3100097	chr4	3249850	3299716		NA
chr15	3100094	3149862	chr4	3199973	3249853		
chr15	3149859	3199885	chr4	3199973	3249853		

chr15	75049941	75099970	chr4	3050773	3099791		
chr15	75049941	75099970	chr5	146250715	146300005		Cdk8,NA
chr15	75049941	75099970	chr6	3199752	3250020		Rn18s
chr15	75049941	75099970	chr9	123449722	123500196		Limd1,Lars2
chr16	3100168	3149912	chr10	22099860	22149739		E030030I06Rik
chr16	3350440	3400083	chr10	22099860	22149739		E030030I06Rik
chr16	3100168	3149912	chr11	54100052	54150476		P4ha2
chr16	3181571	3250185	chr11	3149972	3199980		Sfi1
chr16	3250182	3300027	chr11	3149972	3199980		Sfi1
chr16	3100168	3149912	chr13	3350341	3399887		NA
chr16	3350440	3400083	chr13	3350341	3399887		NA
chr16	3100168	3149912	chr15	75049941	75099970		
chr16	3350440	3400083	chr15	75049941	75099970		
chr16	57350249	57400043	chr15	75049941	75099970	Cmss1,Filip1l	
chr16	3100168	3149912	chr2	181899774	181950034		
chr16	3350440	3400083	chr2	181899774	181950034		
chr16	11100294	11150718	chr2	181899774	181950034	Txndc11,Zc3h7a	
chr16	17199671	17250186	chr2	181899774	181950034	Ube2l3,Hic2,Rimbp3	
chr16	57350249	57400043	chr2	181899774	181950034	Cmss1,Filip1l	
chr16	3100168	3149912	chr3	8199969	8250010		
chr16	3100168	3149912	chr4	3050773	3099791		
chr16	3181571	3250185	chr4	3050773	3099791		
chr16	3181571	3250185	chr5	79399974	79449996		
chr16	3250182	3300027	chr5	79399974	79449996		
chr16	57350249	57400043	chr5	146250715	146300005	Cmss1,Filip1l	Cdk8,NA
chr16	17199671	17250186	chr9	123449722	123500196	Ube2l3,Hic2,Rimbp3	Limd1,Lars2
chr17	3000418	3050005	chr1	88200133	88249820		Dnajb3,Ugt1a2,Ugt1a6a,Ugt1a10,Ugt1a7c,Ugt1a5,Ugt1a9,Ugt1a6b,Ugt1a1,Mroh2a
chr17	3000418	3050005	chr1	88249817	88299855		6430706D22Rik,Hjurp,Mroh2a
chr17	23300132	23349848	chr1	88200133	88249820	Vmn2r115,Vmn2r114	Dnajb3,Ugt1a2,Ugt1a6a,Ugt1a10,Ugt1a7c,Ugt1a5,Ugt1a9,Ugt1a6b,Ugt1a1,Mroh2a
chr17	23300132	23349848	chr1	88249817	88299855	Vmn2r115,Vmn2r114	6430706D22Rik,Hjurp,Mroh2a
chr17	23400235	23450582	chr1	88249817	88299855	Vmn2r116	6430706D22Rik,Hjurp,Mroh2a
chr17	23499743	23549799	chr1	88200133	88249820		Dnajb3,Ugt1a2,Ugt1a6a,Ugt1a10,Ugt1a7c,Ugt1a5,Ugt1a9,Ugt1a6b,Ugt1a1,Mroh2a
chr17	23499743	23549799	chr1	88249817	88299855		6430706D22Rik,Hjurp,Mroh2a
chr17	39800075	39850047	chr1	58599814	58650138	Rn45s	
chr17	39800075	39850047	chr1	102600222	102650516	Rn45s	
chr17	39800075	39850047	chr1	112449969	112500029	Rn45s	
chr17	39800075	39850047	chr1	183249934	183299851	Rn45s	Brox,Aida
chr17	39800075	39850047	chr1	195200096	195249561	Rn45s	
chr17	39800075	39850047	chr10	11450721	11499362	Rn45s	Epm2a
chr17	39800075	39850047	chr10	22099860	22149739	Rn45s	E030030I06Rik

chr17	36199879	36250086	chr11	108999946	109049739	Gm10074	
chr17	39800075	39850047	chr11	3149972	3199980	Rn45s	Sfi1
chr17	39800075	39850047	chr11	54100052	54150476	Rn45s	P4ha2
chr17	39800075	39850047	chr11	106049959	106099962	Rn45s	Map3k3,Taco1,Dcaf7
chr17	39800075	39850047	chr11	108999946	109049739	Rn45s	
chr17	39800075	39850047	chr12	78350565	78400123	Rn45s	Gphn
chr17	39800075	39850047	chr13	3350341	3399887	Rn45s	NA
chr17	39800075	39850047	chr13	9000057	9050074	Rn45s	
chr17	39800075	39850047	chr13	44850383	44900261	Rn45s	Jarid2
chr17	39800075	39850047	chr13	99749094	99800064	Rn45s	
chr17	39800075	39850047	chr15	3149859	3199885	Rn45s	
chr17	39800075	39850047	chr15	3199882	3249847	Rn45s	
chr17	39800075	39850047	chr15	3249844	3300320	Rn45s	Selenop,Ccdc152
chr17	39800075	39850047	chr15	4000420	4049859	Rn45s	Oxct1,A630020A06,BC037032
chr17	39800075	39850047	chr15	4049856	4099829	Rn45s	Oxct1
chr17	39800075	39850047	chr15	5499808	5550210	Rn45s	5430437J10Rik
chr17	39800075	39850047	chr15	5700098	5750113	Rn45s	
chr17	39800075	39850047	chr15	75049941	75099970	Rn45s	
chr17	39800075	39850047	chr16	3100168	3149912	Rn45s	
chr17	39800075	39850047	chr16	3350440	3400083	Rn45s	
chr17	39800075	39850047	chr16	11100294	11150718	Rn45s	Txndc11,Zc3h7a
chr17	39800075	39850047	chr16	17199671	17250186	Rn45s	Ube2l3,Hic2,Rimbp3
chr17	39800075	39850047	chr16	57350249	57400043	Rn45s	Cmss1,Filip1l
chr17	39800075	39850047	chr16	91499244	91549980	Rn45s	Ifnar1,Ifngr2
chr17	36199879	36250086	chr2	181899774	181950034	Gm10074	
chr17	39800075	39850047	chr2	22699938	22750211	Rn45s	
chr17	39800075	39850047	chr2	98649599	98700076	Rn45s	
chr17	39800075	39850047	chr2	102800197	102850133	Rn45s	Cd44
chr17	39800075	39850047	chr2	181899774	181950034	Rn45s	
chr17	39800075	39850047	chr3	5849735	5900077	Rn45s	
chr17	39800075	39850047	chr3	8199969	8250010	Rn45s	
chr17	39800075	39850047	chr3	63500060	63550836	Rn45s	
chr17	39800075	39850047	chr3	152699887	152749949	Rn45s	Pigk
chr17	3000418	3050005	chr4	3050773	3099791		
chr17	39800075	39850047	chr4	3050773	3099791	Rn45s	
chr17	39800075	39850047	chr4	34900007	34950368	Rn45s	Cga,Platr9,Mob3b
chr17	36199879	36250086	chr5	146250715	146300005	Gm10074	Cdk8,NA
chr17	39800075	39850047	chr5	46099875	46149746	Rn45s	
chr17	39800075	39850047	chr5	109550198	109599891	Rn45s	Crlf2
chr17	39800075	39850047	chr5	146250715	146300005	Rn45s	Cdk8,NA

chr17	39800075	39850047	chr6	3199752	3250020	Rn45s	Rn18s
chr17	39800075	39850047	chr6	49199895	49250056	Rn45s	Tra2a,Igf2bp3
chr17	39800075	39850047	chr7	110050177	110100455	Rn45s	Zfp143,Ipo7,AA474408
chr17	39800075	39850047	chr7	128699982	128750169	Rn45s	Sec23ip,Mcmbp
chr17	39800075	39850047	chr7	130800084	130849819	Rn45s	Btbd16,NA
chr17	39800075	39850047	chr8	3750280	3800042	Rn45s	
chr17	39800075	39850047	chr8	15500197	15549866	Rn45s	
chr17	39800075	39850047	chr8	125750083	125800175	Rn45s	Pcnx2
chr17	39800075	39850047	chr9	110250011	110300485	Rn45s	Cspg5
chr17	39800075	39850047	chr9	123449722	123500196	Rn45s	Limd1,Lars2
chr18	3001227	3050237	chr1	195200096	195249561		
chr18	3001227	3050237	chr10	22099860	22149739		E030030I06Rik
chr18	40299987	40350143	chr10	22099860	22149739	Kctd16	E030030I06Rik
chr18	3001227	3050237	chr11	3149972	3199980		Sfi1
chr18	3001227	3050237	chr11	54100052	54150476		P4ha2
chr18	3001227	3050237	chr11	108999946	109049739		
chr18	40299987	40350143	chr11	54100052	54150476	Kctd16	P4ha2
chr18	40299987	40350143	chr11	108999946	109049739	Kctd16	
chr18	3001227	3050237	chr13	3350341	3399887		NA
chr18	3001227	3050237	chr13	9000057	9050074		
chr18	3001227	3050237	chr13	99749094	99800064		
chr18	40299987	40350143	chr13	3350341	3399887	Kctd16	NA
chr18	3001227	3050237	chr15	75049941	75099970		
chr18	40299987	40350143	chr15	75049941	75099970	Kctd16	
chr18	3001227	3050237	chr16	3100168	3149912		
chr18	3001227	3050237	chr16	3181571	3250185		
chr18	3001227	3050237	chr16	3250182	3300027		
chr18	3001227	3050237	chr16	57350249	57400043		Cmss1,Filip1l
chr18	3001227	3050237	chr17	36199879	36250086		Gm10074
chr18	3001227	3050237	chr17	39800075	39850047		Rn45s
chr18	3300451	3350460	chr17	39800075	39850047	Crem,Gm6225	Rn45s
chr18	14099959	14150138	chr17	39800075	39850047		Rn45s
chr18	40299987	40350143	chr17	39800075	39850047	Kctd16	Rn45s
chr18	68649747	68699965	chr17	39800075	39850047		Rn45s
chr18	85650030	85699893	chr17	39800075	39850047		Rn45s
chr18	3001227	3050237	chr2	181899774	181950034		
chr18	40299987	40350143	chr2	181899774	181950034	Kctd16	
chr18	3001227	3050237	chr3	8199969	8250010		
chr18	40299987	40350143	chr3	8199969	8250010	Kctd16	
chr18	3001227	3050237	chr4	3050773	3099791		

chr18	3001227	3050237	chr5	146250715	146300005		Cdk8,NA
chr18	3001227	3050237	chr6	3199752	3250020		Rn18s
chr18	3001227	3050237	chr9	123449722	123500196		Limd1,Lars2
chr19	45649879	45699993	chr10	22099860	22149739	Fbxw4	E030030I06Rik
chr19	61149845	61200309	chr10	22099860	22149739	Gm7102	E030030I06Rik
chr19	6650028	6687996	chr11	22999972	23049848		Cct4,Fam161a
chr19	45649879	45699993	chr11	54100052	54150476	Fbxw4	P4ha2
chr19	52049454	52100277	chr11	3149972	3199980		Sfi1
chr19	45649879	45699993	chr13	3350341	3399887	Fbxw4	NA
chr19	61149845	61200309	chr13	3350341	3399887	Gm7102	NA
chr19	45649879	45699993	chr15	75049941	75099970	Fbxw4	
chr19	61149845	61200309	chr15	75049941	75099970	Gm7102	
chr19	61200306	61250122	chr15	75049941	75099970	Csf2ra,Gm4242	
chr19	61250119	61299557	chr15	75049941	75099970		
chr19	3098793	3150428	chr16	3250182	3300027		
chr19	31299604	31350062	chr17	39800075	39850047	Prkg1	Rn45s
chr19	45649879	45699993	chr17	39800075	39850047	Fbxw4	Rn45s
chr19	61149845	61200309	chr17	39800075	39850047	Gm7102	Rn45s
chr19	61200306	61250122	chr17	39800075	39850047	Csf2ra,Gm4242	Rn45s
chr19	61250119	61299557	chr17	39800075	39850047		Rn45s
chr19	45649879	45699993	chr18	3001227	3050237	Fbxw4	
chr19	45649879	45699993	chr18	40299987	40350143	Fbxw4	Kctd16
chr19	61149845	61200309	chr18	3001227	3050237	Gm7102	
chr19	61250119	61299557	chr18	3001227	3050237		
chr19	45649879	45699993	chr2	181899774	181950034	Fbxw4	
chr19	61149845	61200309	chr2	181899774	181950034	Gm7102	
chr19	61200306	61250122	chr2	181899774	181950034	Csf2ra,Gm4242	
chr19	61250119	61299557	chr2	181899774	181950034		
chr19	45649879	45699993	chr3	8199969	8250010	Fbxw4	
chr2	181899774	181950034	chr1	58599814	58650138		
chr2	181899774	181950034	chr1	183249934	183299851		Brox,Aida
chr2	181899774	181950034	chr1	195200096	195249561		
chr3	3000788	3050004	chr1	88200133	88249820		Dnajb3,Ugt1a2,Ugt1a6a,Ugt1a10,Ugt1a7c,Ugt1a5,Ugt1a9,Ugt1a6b,Ugt1a1,Mroh2a
chr3	3000788	3050004	chr1	88249817	88299855		6430706D22Rik,Hjurp,Mroh2a
chr3	3050001	3099957	chr1	88249817	88299855		6430706D22Rik,Hjurp,Mroh2a
chr3	3200068	3249959	chr1	88200133	88249820		Dnajb3,Ugt1a2,Ugt1a6a,Ugt1a10,Ugt1a7c,Ugt1a5,Ugt1a9,Ugt1a6b,Ugt1a1,Mroh2a
chr3	3200068	3249959	chr1	88249817	88299855		6430706D22Rik,Hjurp,Mroh2a
chr3	8199969	8250010	chr1	195200096	195249561		
chr3	8199969	8250010	chr2	181899774	181950034		
chr3	63500060	63550836	chr2	181899774	181950034		

chr3	152699887	152749949	chr2	181899774	181950034	Pigk		
chr4	3050773	3099791	chr1	88200133	88249820			Dnajb3,Ugt1a2,Ugt1a6a,Ugt1a10,Ugt1a7c,Ugt1a5,Ugt1a9,Ugt1a6b,Ugt1a1,Mroh2a
chr4	3050773	3099791	chr1	88249817	88299855			6430706D22Rik,Hjurp,Mroh2a
chr4	3050773	3099791	chr2	181899774	181950034			
chr4	34900007	34950368	chr2	181899774	181950034	Cga,Platr9,Mob3b		
chr4	3050773	3099791	chr3	8199969	8250010			
chr5	146250715	146300005	chr2	181899774	181950034	Cdk8,NA		
chr5	146250715	146300005	chr3	8199969	8250010	Cdk8,NA		
chr5	77400011	77449890	chr4	3199973	3249853	Igfbp7		
chr6	3199752	3250020	chr2	181899774	181950034	Rn18s		
chr6	103599835	103649972	chr2	98649599	98700076	Chl1		
chr6	3050001	3099781	chr4	3050773	3099791			
chr6	3149518	3199755	chr4	3199973	3249853	Rn18s		
chr6	3399884	3449758	chr4	3050773	3099791			
chr6	58550148	58600110	chr4	147400251	147449702	Abcg2		
chr6	58600107	58650375	chr4	146450134	146499188	Abcg2		Zfp992
chr6	58600107	58650375	chr4	147400251	147449702	Abcg2		
chr6	67649862	67699998	chr4	146450134	146499188			Zfp992
chr6	67649862	67699998	chr4	147400251	147449702			
chr7	128699982	128750169	chr2	181899774	181950034	Sec23ip,Mcmbp		
chr7	3000001	3049771	chr4	3199973	3249853			
chr8	125750083	125800175	chr2	181899774	181950034	Pcnx2		
chr9	3300202	3349883	chr1	88200133	88249820	Alkbh8		Dnajb3,Ugt1a2,Ugt1a6a,Ugt1a10,Ugt1a7c,Ugt1a5,Ugt1a9,Ugt1a6b,Ugt1a1,Mroh2a
chr9	3300202	3349883	chr1	88249817	88299855	Alkbh8		6430706D22Rik,Hjurp,Mroh2a
chr9	3000001	3049479	chr2	98649599	98700076	Mir101c		
chr9	24501162	24550023	chr2	98649599	98700076	Dpy19l1		
chr9	123449722	123500196	chr2	181899774	181950034	Limd1,Lars2		
chr9	123449722	123500196	chr3	5849735	5900077	Limd1,Lars2		
chr9	123449722	123500196	chr3	8199969	8250010	Limd1,Lars2		
chr9	24501162	24550023	chr6	103599835	103649972	Dpy19l1		Chl1
chrX	4849977	4899691	chr17	39800075	39850047			Rn45s
chrX	87199968	87250042	chr17	39800075	39850047	Il1rapl1		Rn45s
chrX	143450048	143500816	chr2	98649599	98700076			
chrX	124100008	124149871	chr4	156306042	156349983	Vmn2r121		Vmn2r-ps159,Vmn2r125
chrX	143450048	143500816	chr6	103599835	103649972			Chl1
chrX	143450048	143500816	chr9	24501162	24550023			Dpy19l1

Supplemental Table 1: Detected transchromosomal interactions in mouse CD8+ T cells							
Chromosome	Start	End	Chromosome	Start	End	Gene associated with first anchor	Gene associated with second anchor
chr10	22099860	22149739	chr1	183249934	183299851	E030030I06Rik	Brox,Aida
chr10	22099860	22149739	chr2	181899774	181950034	E030030I06Rik	
chr10	22099860	22149739	chr3	8199969	8250010	E030030I06Rik	
chr10	22099860	22149739	chr4	3050773	3099791	E030030I06Rik	
chr10	11450721	11499362	chr5	146250715	146300005	Epm2a	Cdk8,NA
chr10	22099860	22149739	chr5	146250715	146300005	E030030I06Rik	Cdk8,NA
chr11	3149972	3199980	chr1	88200133	88249820	Sfi1	Dnajb3,Ugt1a2,Ugt1a6a,Ugt1a10,Ugt1a7c,Ugt1a5,Ugt1a9,Ugt1a6b,Ugt1a1,Mroh2a
chr11	3149972	3199980	chr1	88249817	88299855	Sfi1	6430706D22Rik,Hjurp,Mroh2a
chr11	54100052	54150476	chr1	183249934	183299851	P4ha2	Brox,Aida
chr11	3149972	3199980	chr10	22099860	22149739	Sfi1	E030030I06Rik
chr11	54100052	54150476	chr10	22099860	22149739	P4ha2	E030030I06Rik
chr11	108999946	109049739	chr10	22099860	22149739		E030030I06Rik
chr11	3102115	3149975	chr2	3050197	3099735	Sfi1,Pisd-ps1	
chr11	3149972	3199980	chr2	98649599	98700076	Sfi1	
chr11	3149972	3199980	chr2	181899774	181950034	Sfi1	
chr11	54100052	54150476	chr2	181899774	181950034	P4ha2	
chr11	108999946	109049739	chr2	181899774	181950034		
chr11	3149972	3199980	chr3	8199969	8250010	Sfi1	
chr11	54100052	54150476	chr3	8199969	8250010	P4ha2	
chr11	108999946	109049739	chr3	5849735	5900077		
chr11	108999946	109049739	chr3	8199969	8250010		
chr11	3149972	3199980	chr4	3050773	3099791	Sfi1	
chr11	3149972	3199980	chr4	3199973	3249853	Sfi1	
chr11	3149972	3199980	chr4	141100135	141149689	Sfi1	Szrd1,Fbxo42,Spata21
chr11	108999946	109049739	chr4	3050773	3099791		
chr11	3149972	3199980	chr5	135350081	135400045	Sfi1	Nsun5,Pom121,Trim50
chr11	54100052	54150476	chr5	146250715	146300005	P4ha2	Cdk8,NA
chr11	108999946	109049739	chr5	146250715	146300005		Cdk8,NA
chr11	3149972	3199980	chr6	3199752	3250020	Sfi1	Rn18s
chr11	3149972	3199980	chr6	3349828	3399887	Sfi1	Samd9l
chr11	3149972	3199980	chr6	103599835	103649972	Sfi1	Chl1
chr11	3149972	3199980	chr9	3000001	3049479	Sfi1	Mir101c
chr11	3149972	3199980	chr9	35299842	35350067	Sfi1	
chr11	108999946	109049739	chr9	123449722	123500196		Limd1,Lars2
chr12	51450299	51499933	chr1	88249817	88299855		6430706D22Rik,Hjurp,Mroh2a
chr12	119550007	119600030	chr1	88200133	88249820		Dnajb3,Ugt1a2,Ugt1a6a,Ugt1a10,Ugt1a7c,Ugt1a5,Ugt1a9,Ugt1a6b,Ugt1a1,Mroh2a
chr12	27000075	27050265	chr11	3149972	3199980		Sfi1
chr12	20200108	20249248	chr4	145650000	145699976	NA	Zfp980

chr12	20200108	20249248	chr4	146149438	146200078	NA	Zfp600
chr12	20200108	20249248	chr4	146450134	146499188	NA	Zfp992
chr12	20200108	20249248	chr4	146499185	146549996	NA	1700095A21Rik,Zfp981
chr12	20200108	20249248	chr4	146708801	146750182	NA	NA,Gm20875
chr12	20200108	20249248	chr4	146900733	146950126	NA	Gm13149,NA
chr12	20200108	20249248	chr4	147299595	147350006	NA	
chr12	20200108	20249248	chr4	147400251	147449702	NA	
chr12	20200108	20249248	chr4	147750002	147800228	NA	Zfp984
chr12	3000001	3049529	chr5	3000001	3050004		V1rg10
chr13	3350341	3399887	chr1	183249934	183299851	NA	Brox,Aida
chr13	3350341	3399887	chr1	195200096	195249561	NA	
chr13	9000057	9050074	chr1	195200096	195249561		
chr13	3350341	3399887	chr10	22099860	22149739	NA	E030030I06Rik
chr13	9000057	9050074	chr10	22099860	22149739		E030030I06Rik
chr13	99749094	99800064	chr10	22099860	22149739		E030030I06Rik
chr13	3350341	3399887	chr11	3149972	3199980	NA	Sfi1
chr13	3350341	3399887	chr11	54100052	54150476	NA	P4ha2
chr13	3350341	3399887	chr11	108999946	109049739	NA	
chr13	9000057	9050074	chr11	54100052	54150476		P4ha2
chr13	9000057	9050074	chr11	108999946	109049739		
chr13	12849190	12900036	chr11	3149972	3199980		Sfi1
chr13	3350341	3399887	chr2	181899774	181950034	NA	
chr13	9000057	9050074	chr2	181899774	181950034		
chr13	44850383	44900261	chr2	181899774	181950034	Jarid2	
chr13	99749094	99800064	chr2	181899774	181950034		
chr13	3350341	3399887	chr3	5849735	5900077	NA	
chr13	3350341	3399887	chr3	8199969	8250010	NA	
chr13	9000057	9050074	chr3	8199969	8250010		
chr13	99749094	99800064	chr3	8199969	8250010		
chr13	3000480	3050284	chr4	3199973	3249853		
chr13	3350341	3399887	chr4	3050773	3099791	NA	
chr13	3350341	3399887	chr5	146250715	146300005	NA	Cdk8,NA
chr13	9000057	9050074	chr5	146250715	146300005		Cdk8,NA
chr13	3000480	3050284	chr6	3149518	3199755		Rn18s
chr13	3350341	3399887	chr9	123449722	123500196	NA	Limd1,Lars2
chr13	119595537	119599781	chr9	123650116	123700427	Tmem267	Lztfl1,Slc6a20a,Gm10052
chr13	119595537	119599781	chr9	123949939	123999999	Tmem267	Ccr1,Ccr1l1
chr13	119595537	119599781	chr9	124250486	124300017	Tmem267	2010315B03Rik
chr13	119599778	119649908	chr9	124100072	124149813	Ccl28,Tmem267	Ccr2,Ccr5
chr13	119599778	119649908	chr9	124250486	124300017	Ccl28,Tmem267	2010315B03Rik

chr14	19350468	19399525	chr11	3149972	3199980		Sfi1
chr14	19399522	19469708	chr11	3149972	3199980		Sfi1
chr14	19399522	19469708	chr9	3000001	3049479		Mir101c
chr15	75049941	75099970	chr1	58599814	58650138		
chr15	75049941	75099970	chr1	183249934	183299851		Brox,Aida
chr15	75049941	75099970	chr1	195200096	195249561		
chr15	75049941	75099970	chr10	22099860	22149739		E030030I06Rik
chr15	13799860	13850087	chr11	3149972	3199980		Sfi1
chr15	75049941	75099970	chr11	3149972	3199980		Sfi1
chr15	75049941	75099970	chr11	54100052	54150476		P4ha2
chr15	75049941	75099970	chr11	108999946	109049739		
chr15	103249683	103299695	chr11	54100052	54150476	Nfe2,Copz1,Mir148b,NA	P4ha2
chr15	75049941	75099970	chr13	3350341	3399887		NA
chr15	75049941	75099970	chr13	9000057	9050074		
chr15	75049941	75099970	chr13	99749094	99800064		
chr15	75049941	75099970	chr2	22699938	22750211		
chr15	75049941	75099970	chr2	181899774	181950034		
chr15	75049941	75099970	chr3	5849735	5900077		
chr15	75049941	75099970	chr3	8199969	8250010		
chr15	75049941	75099970	chr3	63500060	63550836		
chr15	75049941	75099970	chr3	152699887	152749949		Pigk
chr15	3050001	3100097	chr4	3199973	3249853		
chr15	3050001	3100097	chr4	3249850	3299716		NA
chr15	3149859	3199885	chr4	3199973	3249853		
chr15	75049941	75099970	chr4	3050773	3099791		
chr15	75049941	75099970	chr4	34900007	34950368		Cga,Platr9,Mob3b
chr15	75049941	75099970	chr5	146250715	146300005		Cdk8,NA
chr15	75049941	75099970	chr6	3199752	3250020		Rn18s
chr15	75049941	75099970	chr6	49199895	49250056		Tra2a,Igf2bp3
chr15	75049941	75099970	chr8	125750083	125800175		Pcnx2
chr15	75049941	75099970	chr9	123449722	123500196		Limd1,Lars2
chr16	3100168	3149912	chr10	22099860	22149739		E030030I06Rik
chr16	3350440	3400083	chr10	22099860	22149739		E030030I06Rik
chr16	57350249	57400043	chr10	22099860	22149739	Cmss1,Filip1l	E030030I06Rik
chr16	3100168	3149912	chr11	54100052	54150476		P4ha2
chr16	3100168	3149912	chr11	108999946	109049739		
chr16	3181571	3250185	chr11	3149972	3199980		Sfi1
chr16	3250182	3300027	chr11	3149972	3199980		Sfi1
chr16	11100294	11150718	chr11	108999946	109049739	Txndc11,Zc3h7a	
chr16	17199671	17250186	chr11	108999946	109049739	Ube2l3,Hic2,Rimbp3	

chr16	57350249	57400043	chr11	108999946	109049739	Cmss1,Filip1l	
chr16	3100168	3149912	chr13	3350341	3399887		NA
chr16	3100168	3149912	chr13	9000057	9050074		
chr16	11100294	11150718	chr13	3350341	3399887	Txndc11,Zc3h7a	NA
chr16	17199671	17250186	chr13	3350341	3399887	Ube2l3,Hic2,Rimbp3	NA
chr16	57350249	57400043	chr13	3350341	3399887	Cmss1,Filip1l	NA
chr16	57350249	57400043	chr13	44850383	44900261	Cmss1,Filip1l	Jarid2
chr16	3100168	3149912	chr15	75049941	75099970		
chr16	3300024	3350443	chr15	75049941	75099970		
chr16	3350440	3400083	chr15	75049941	75099970		
chr16	11100294	11150718	chr15	75049941	75099970	Txndc11,Zc3h7a	
chr16	57350249	57400043	chr15	75049941	75099970	Cmss1,Filip1l	
chr16	91499244	91549980	chr15	75049941	75099970	Ifnar1,Ifngr2	
chr16	3100168	3149912	chr2	181899774	181950034		
chr16	3181571	3250185	chr2	181899774	181950034		
chr16	3350440	3400083	chr2	181899774	181950034		
chr16	11100294	11150718	chr2	181899774	181950034	Txndc11,Zc3h7a	
chr16	17199671	17250186	chr2	181899774	181950034	Ube2l3,Hic2,Rimbp3	
chr16	57350249	57400043	chr2	181899774	181950034	Cmss1,Filip1l	
chr16	91499244	91549980	chr2	181899774	181950034	Ifnar1,Ifngr2	
chr16	3100168	3149912	chr3	8199969	8250010		
chr16	3350440	3400083	chr3	8199969	8250010		
chr16	11100294	11150718	chr3	8199969	8250010	Txndc11,Zc3h7a	
chr16	17199671	17250186	chr3	8199969	8250010	Ube2l3,Hic2,Rimbp3	
chr16	57350249	57400043	chr3	8199969	8250010	Cmss1,Filip1l	
chr16	3100168	3149912	chr4	3050773	3099791		
chr16	3250182	3300027	chr5	79399974	79449996		
chr16	11100294	11150718	chr5	146250715	146300005	Txndc11,Zc3h7a	Cdk8,NA
chr16	57350249	57400043	chr5	146250715	146300005	Cmss1,Filip1l	Cdk8,NA
chr16	57350249	57400043	chr6	3199752	3250020	Cmss1,Filip1l	Rn18s
chr17	3000418	3050005	chr1	88200133	88249820		Dnajb3,Ugt1a2,Ugt1a6a,Ugt1a10,Ugt1a7c,Ugt1a5,Ugt1a9,Ugt1a6b,Ugt1a1,Mroh2a
chr17	3000418	3050005	chr1	88249817	88299855		6430706D22Rik,Hjurp,Mroh2a
chr17	23300132	23349848	chr1	88200133	88249820	Vmn2r115,Vmn2r114	Dnajb3,Ugt1a2,Ugt1a6a,Ugt1a10,Ugt1a7c,Ugt1a5,Ugt1a9,Ugt1a6b,Ugt1a1,Mroh2a
chr17	23300132	23349848	chr1	88249817	88299855	Vmn2r115,Vmn2r114	6430706D22Rik,Hjurp,Mroh2a
chr17	23499743	23549799	chr1	88200133	88249820		Dnajb3,Ugt1a2,Ugt1a6a,Ugt1a10,Ugt1a7c,Ugt1a5,Ugt1a9,Ugt1a6b,Ugt1a1,Mroh2a
chr17	23499743	23549799	chr1	88249817	88299855		6430706D22Rik,Hjurp,Mroh2a
chr17	39800075	39850047	chr1	58599814	58650138	Rn45s	
chr17	39800075	39850047	chr1	102600222	102650516	Rn45s	
chr17	39800075	39850047	chr1	112449969	112500029	Rn45s	
chr17	39800075	39850047	chr1	183249934	183299851	Rn45s	Brox,Aida

chr17	39800075	39850047	chr1	195200096	195249561	Rn45s	
chr17	36199879	36250086	chr10	22099860	22149739	Gm10074	E030030I06Rik
chr17	39800075	39850047	chr10	11450721	11499362	Rn45s	Epm2a
chr17	39800075	39850047	chr10	22099860	22149739	Rn45s	E030030I06Rik
chr17	3050002	3099978	chr11	3149972	3199980	Pisd-ps2	Sfi1
chr17	36199879	36250086	chr11	108999946	109049739	Gm10074	
chr17	39800075	39850047	chr11	3149972	3199980	Rn45s	Sfi1
chr17	39800075	39850047	chr11	19999990	20050262	Rn45s	Spred2
chr17	39800075	39850047	chr11	54100052	54150476	Rn45s	P4ha2
chr17	39800075	39850047	chr11	106049959	106099962	Rn45s	Map3k3,Taco1,Dcaf7
chr17	39800075	39850047	chr11	108999946	109049739	Rn45s	
chr17	39800075	39850047	chr12	3349958	3399929	Rn45s	Kif3c
chr17	39800075	39850047	chr12	36849926	36900149	Rn45s	
chr17	39800075	39850047	chr12	78350565	78400123	Rn45s	Gphn
chr17	39800075	39850047	chr13	3350341	3399887	Rn45s	NA
chr17	39800075	39850047	chr13	9000057	9050074	Rn45s	
chr17	39800075	39850047	chr13	44850383	44900261	Rn45s	Jarid2
chr17	39800075	39850047	chr13	97149591	97200063	Rn45s	Hexb,Gfm2
chr17	39800075	39850047	chr13	99749094	99800064	Rn45s	
chr17	39800075	39850047	chr14	37850224	37900301	Rn45s	
chr17	36199879	36250086	chr15	75049941	75099970	Gm10074	
chr17	39800075	39850047	chr15	3149859	3199885	Rn45s	
chr17	39800075	39850047	chr15	3199882	3249847	Rn45s	
chr17	39800075	39850047	chr15	3249844	3300320	Rn45s	Selenop,Ccdc152
chr17	39800075	39850047	chr15	3950092	4000423	Rn45s	Fbxo4,AW549877,A630020A06
chr17	39800075	39850047	chr15	4000420	4049859	Rn45s	Oxct1,A630020A06,BC037032
chr17	39800075	39850047	chr15	4999963	5049403	Rn45s	C7
chr17	39800075	39850047	chr15	5550207	5599835	Rn45s	5430437J10Rik
chr17	39800075	39850047	chr15	50649998	50699885	Rn45s	Trps1
chr17	39800075	39850047	chr15	75049941	75099970	Rn45s	
chr17	39800075	39850047	chr16	3100168	3149912	Rn45s	
chr17	39800075	39850047	chr16	3181571	3250185	Rn45s	
chr17	39800075	39850047	chr16	3350440	3400083	Rn45s	
chr17	39800075	39850047	chr16	11100294	11150718	Rn45s	Txndc11,Zc3h7a
chr17	39800075	39850047	chr16	11499749	11549892	Rn45s	Snx29
chr17	39800075	39850047	chr16	17199671	17250186	Rn45s	Ube2l3,Hic2,Rimbp3
chr17	39800075	39850047	chr16	26699927	26750152	Rn45s	Il1rap,Gm20319
chr17	39800075	39850047	chr16	57350249	57400043	Rn45s	Cmss1,Filip1l
chr17	39800075	39850047	chr16	91499244	91549980	Rn45s	Ifnar1,Ifngr2
chr17	36199879	36250086	chr2	181899774	181950034	Gm10074	

chr17	39800075	39850047	chr2	22699938	22750211	Rn45s	
chr17	39800075	39850047	chr2	98649599	98700076	Rn45s	
chr17	39800075	39850047	chr2	102800197	102850133	Rn45s	Cd44
chr17	39800075	39850047	chr2	181899774	181950034	Rn45s	
chr17	36199879	36250086	chr3	8199969	8250010	Gm10074	
chr17	39800075	39850047	chr3	5849735	5900077	Rn45s	
chr17	39800075	39850047	chr3	8199969	8250010	Rn45s	
chr17	39800075	39850047	chr3	63500060	63550836	Rn45s	
chr17	39800075	39850047	chr3	152699887	152749949	Rn45s	Pigk
chr17	3000418	3050005	chr4	3050773	3099791		
chr17	39800075	39850047	chr4	3050773	3099791	Rn45s	
chr17	39800075	39850047	chr4	34900007	34950368	Rn45s	Cga,Platr9,Mob3b
chr17	39800075	39850047	chr4	123749855	123800155	Rn45s	Akirin1,Rhbdl2
chr17	36199879	36250086	chr5	146250715	146300005	Gm10074	Cdk8,NA
chr17	39800075	39850047	chr5	46099875	46149746	Rn45s	
chr17	39800075	39850047	chr5	109550198	109599891	Rn45s	Crlf2
chr17	39800075	39850047	chr5	146250715	146300005	Rn45s	Cdk8,NA
chr17	39800075	39850047	chr6	3199752	3250020	Rn45s	Rn18s
chr17	39800075	39850047	chr6	49199895	49250056	Rn45s	Tra2a,Igf2bp3
chr17	39800075	39850047	chr6	55550033	55600083	Rn45s	
chr17	39800075	39850047	chr7	110050177	110100455	Rn45s	Zfp143,Ipo7,AA474408
chr17	39800075	39850047	chr7	128699982	128750169	Rn45s	Sec23ip,Mcmbp
chr17	39800075	39850047	chr7	130800084	130849819	Rn45s	Btbd16,NA
chr17	39800075	39850047	chr8	3750280	3800042	Rn45s	
chr17	39800075	39850047	chr8	15500197	15549866	Rn45s	
chr17	39800075	39850047	chr8	125750083	125800175	Rn45s	Pcnx2
chr17	39800075	39850047	chr9	110250011	110300485	Rn45s	Cspg5
chr17	39800075	39850047	chr9	123449722	123500196	Rn45s	Limd1,Lars2
chr18	3001227	3050237	chr1	58599814	58650138		
chr18	3001227	3050237	chr1	195200096	195249561		
chr18	3001227	3050237	chr10	22099860	22149739		E030030I06Rik
chr18	40299987	40350143	chr10	22099860	22149739	Kctd16	E030030I06Rik
chr18	3001227	3050237	chr11	3149972	3199980		Sfi1
chr18	3001227	3050237	chr11	54100052	54150476		P4ha2
chr18	3001227	3050237	chr11	108999946	109049739		
chr18	3650329	3700227	chr11	3149972	3199980		Sfi1
chr18	7000467	7050018	chr11	3149972	3199980	Mkx	Sfi1
chr18	8399939	8449940	chr11	3149972	3199980		Sfi1
chr18	40299987	40350143	chr11	54100052	54150476	Kctd16	P4ha2
chr18	40299987	40350143	chr11	108999946	109049739	Kctd16	

chr18	3001227	3050237	chr13	3000480	3050284		
chr18	3001227	3050237	chr13	3350341	3399887		NA
chr18	3001227	3050237	chr13	9000057	9050074		
chr18	3001227	3050237	chr13	99749094	99800064		
chr18	40299987	40350143	chr13	3350341	3399887	Kctd16	NA
chr18	40299987	40350143	chr13	9000057	9050074	Kctd16	
chr18	3001227	3050237	chr15	75049941	75099970		
chr18	40299987	40350143	chr15	75049941	75099970	Kctd16	
chr18	68649747	68699965	chr15	75049941	75099970		
chr18	3001227	3050237	chr16	3100168	3149912		
chr18	3001227	3050237	chr16	3181571	3250185		
chr18	3001227	3050237	chr16	3250182	3300027		
chr18	3001227	3050237	chr16	11100294	11150718		Txndc11,Zc3h7a
chr18	3001227	3050237	chr16	57350249	57400043		Cmss1,Filip1l
chr18	3001227	3050237	chr17	36199879	36250086		Gm10074
chr18	3001227	3050237	chr17	39800075	39850047		Rn45s
chr18	3300451	3350460	chr17	39800075	39850047	Crem,Gm6225	Rn45s
chr18	3350457	3400233	chr17	39800075	39850047	Cul2,Gm6225	Rn45s
chr18	12900001	12949796	chr17	39800075	39850047	Osbpl1a	Rn45s
chr18	14099959	14150138	chr17	39800075	39850047		Rn45s
chr18	40299987	40350143	chr17	39800075	39850047	Kctd16	Rn45s
chr18	68649747	68699965	chr17	39800075	39850047		Rn45s
chr18	73499308	73549982	chr17	39800075	39850047		Rn45s
chr18	85650030	85699893	chr17	39800075	39850047		Rn45s
chr18	3001227	3050237	chr2	181899774	181950034		
chr18	40299987	40350143	chr2	181899774	181950034	Kctd16	
chr18	68649747	68699965	chr2	181899774	181950034		
chr18	3001227	3050237	chr3	8199969	8250010		
chr18	3001227	3050237	chr3	63500060	63550836		
chr18	40299987	40350143	chr3	8199969	8250010	Kctd16	
chr18	3001227	3050237	chr4	3050773	3099791		
chr18	3001227	3050237	chr5	146250715	146300005		Cdk8,NA
chr18	40299987	40350143	chr5	146250715	146300005	Kctd16	Cdk8,NA
chr18	3001227	3050237	chr6	3050001	3099781		
chr18	3001227	3050237	chr6	3199752	3250020		Rn18s
chr18	3001227	3050237	chr9	123449722	123500196		Limd1,Lars2
chr19	61149845	61200309	chr10	22099860	22149739	Gm7102	E030030I06Rik
chr19	19699726	19749863	chr11	3149972	3199980		Sfi1
chr19	48999779	49050046	chr11	3149972	3199980		Sfi1
chr19	49349792	49399957	chr11	3149972	3199980		Sfi1

chr19	49599931	49649844	chr11	3149972	3199980		Sfi1
chr19	51300018	51349614	chr11	3149972	3199980		Sfi1
chr19	61149845	61200309	chr13	9000057	9050074	Gm7102	
chr19	45649879	45699993	chr15	75049941	75099970	Fbxw4	
chr19	61149845	61200309	chr15	75049941	75099970	Gm7102	
chr19	61200306	61250122	chr15	75049941	75099970	Csf2ra,Gm4242	
chr19	61250119	61299557	chr15	75049941	75099970		
chr19	3150425	3200160	chr16	3250182	3300027	1700030N03Rik	
chr19	13849927	13901168	chr16	3250182	3300027	Olfr1504,Olfr1502	
chr19	49050043	49100267	chr16	3250182	3300027		
chr19	8249634	8300361	chr17	39800075	39850047		Rn45s
chr19	45649879	45699993	chr17	39800075	39850047	Fbxw4	Rn45s
chr19	61149845	61200309	chr17	39800075	39850047	Gm7102	Rn45s
chr19	61250119	61299557	chr17	39800075	39850047		Rn45s
chr19	45649879	45699993	chr18	3001227	3050237	Fbxw4	
chr19	45649879	45699993	chr18	40299987	40350143	Fbxw4	Kctd16
chr19	61149845	61200309	chr18	3001227	3050237	Gm7102	
chr19	61250119	61299557	chr18	3001227	3050237		
chr19	45649879	45699993	chr2	181899774	181950034	Fbxw4	
chr19	61149845	61200309	chr2	181899774	181950034	Gm7102	
chr19	61250119	61299557	chr2	181899774	181950034		
chr19	45649879	45699993	chr3	8199969	8250010	Fbxw4	
chr2	3050197	3099735	chr1	88200133	88249820		Dnajb3,Ugt1a2,Ugt1a6a,Ugt1a10,Ugt1a7c,Ugt1a5,Ugt1a9,Ugt1a6b,Ugt1a1,Mroh2a
chr2	181899774	181950034	chr1	58599814	58650138		
chr2	181899774	181950034	chr1	102600222	102650516		
chr2	181899774	181950034	chr1	112449969	112500029		
chr2	181899774	181950034	chr1	183249934	183299851		Brox,Aida
chr2	181899774	181950034	chr1	195200096	195249561		
chr3	3000788	3050004	chr1	88200133	88249820		Dnajb3,Ugt1a2,Ugt1a6a,Ugt1a10,Ugt1a7c,Ugt1a5,Ugt1a9,Ugt1a6b,Ugt1a1,Mroh2a
chr3	3000788	3050004	chr1	88249817	88299855		6430706D22Rik,Hjurp,Mroh2a
chr3	3050001	3099957	chr1	88200133	88249820		Dnajb3,Ugt1a2,Ugt1a6a,Ugt1a10,Ugt1a7c,Ugt1a5,Ugt1a9,Ugt1a6b,Ugt1a1,Mroh2a
chr3	3099954	3149828	chr1	88249817	88299855		6430706D22Rik,Hjurp,Mroh2a
chr3	3200068	3249959	chr1	88200133	88249820		Dnajb3,Ugt1a2,Ugt1a6a,Ugt1a10,Ugt1a7c,Ugt1a5,Ugt1a9,Ugt1a6b,Ugt1a1,Mroh2a
chr3	3200068	3249959	chr1	88249817	88299855		6430706D22Rik,Hjurp,Mroh2a
chr3	8199969	8250010	chr1	183249934	183299851		Brox,Aida
chr3	8199969	8250010	chr1	195200096	195249561		
chr3	14650284	14700461	chr1	88249817	88299855	Car13	6430706D22Rik,Hjurp,Mroh2a
chr3	5849735	5900077	chr2	181899774	181950034		
chr3	8199969	8250010	chr2	181899774	181950034		
chr3	63500060	63550836	chr2	181899774	181950034		

chr3	152699887	152749949	chr2	181899774	181950034	Pigk	
chr4	3050773	3099791	chr1	88200133	88249820		Dnajb3,Ugt1a2,Ugt1a6a,Ugt1a10,Ugt1a7c,Ugt1a5,Ugt1a9,Ugt1a6b,Ugt1a1,Mroh2a
chr4	3050773	3099791	chr1	88249817	88299855		6430706D22Rik,Hjurp,Mroh2a
chr4	3050773	3099791	chr2	181899774	181950034		
chr4	34900007	34950368	chr2	181899774	181950034	Cga,Platr9,Mob3b	
chr4	3050773	3099791	chr3	8199969	8250010		
chr4	34900007	34950368	chr3	8199969	8250010	Cga,Platr9,Mob3b	
chr5	146250715	146300005	chr2	181899774	181950034	Cdk8,NA	
chr5	146250715	146300005	chr3	5849735	5900077	Cdk8,NA	
chr5	146250715	146300005	chr3	8199969	8250010	Cdk8,NA	
chr5	77400011	77449890	chr4	3199973	3249853	Igfbp7	
chr5	146250715	146300005	chr4	3050773	3099791	Cdk8,NA	
chr6	3199752	3250020	chr2	181899774	181950034	Rn18s	
chr6	103599835	103649972	chr2	98649599	98700076	Chl1	
chr6	3050001	3099781	chr4	3050773	3099791		
chr6	3149518	3199755	chr4	3199973	3249853	Rn18s	
chr6	3399884	3449758	chr4	3050773	3099791		
chr6	3449755	3499596	chr4	3050773	3099791	Vps50,Hepacam2	
chr6	58550148	58600110	chr4	147400251	147449702	Abcg2	
chr6	58600107	58650375	chr4	146450134	146499188	Abcg2	Zfp992
chr6	58600107	58650375	chr4	147099542	147150197	Abcg2	Zfp991
chr6	58600107	58650375	chr4	147400251	147449702	Abcg2	
chr6	67649862	67699998	chr4	146450134	146499188		Zfp992
chr6	67649862	67699998	chr4	146499185	146549996		1700095A21Rik,Zfp981
chr6	67649862	67699998	chr4	147400251	147449702		
chr7	110050177	110100455	chr2	181899774	181950034	Zfp143,Ipo7,AA474408	
chr7	128699982	128750169	chr2	181899774	181950034	Sec23ip,Mcmbp	
chr7	3000001	3049771	chr4	3199973	3249853		
chr7	3049768	3100089	chr4	3199973	3249853		
chr8	125750083	125800175	chr2	181899774	181950034	Pcnx2	
chr8	3099911	3150195	chr4	3249850	3299716		NA
chr9	3300202	3349883	chr1	88200133	88249820	Alkbh8	Dnajb3,Ugt1a2,Ugt1a6a,Ugt1a10,Ugt1a7c,Ugt1a5,Ugt1a9,Ugt1a6b,Ugt1a1,Mroh2a
chr9	3300202	3349883	chr1	88249817	88299855	Alkbh8	6430706D22Rik,Hjurp,Mroh2a
chr9	3000001	3049479	chr2	98649599	98700076	Mir101c	
chr9	24501162	24550023	chr2	98649599	98700076	Dpy19l1	
chr9	110250011	110300485	chr2	181899774	181950034	Cspg5	
chr9	123449722	123500196	chr2	181899774	181950034	Limd1,Lars2	
chr9	123449722	123500196	chr3	5849735	5900077	Limd1,Lars2	
chr9	123449722	123500196	chr3	8199969	8250010	Limd1,Lars2	
chr9	24501162	24550023	chr6	103599835	103649972	Dpy19l1	Chl1

chrX	143450048	143500816	chr11	3149972	3199980		Sfi1
chrX	4849977	4899691	chr17	39800075	39850047		Rn45s
chrX	87199968	87250042	chr17	39800075	39850047	Il1rapl1	Rn45s
chrX	112000179	112050116	chr17	39800075	39850047	L1Md-Gf21	Rn45s
chrX	143450048	143500816	chr2	98649599	98700076		
chrX	124100008	124149871	chr4	156306042	156349983	Vmn2r121	Vmn2r-ps159,Vmn2r125
chrX	143450048	143500816	chr6	103599835	103649972		Chl1
chrX	143450048	143500816	chr9	24501162	24550023		Dpy19l1
chrY	90699748	90750101	chr13	119599778	119649908	NA	Ccl28,Tmem267
chrY	90799954	90844698	chr13	119599778	119649908	Erdr1	Ccl28,Tmem267
