# Supplementary material for: Genome-wide analysis reveals no evidence of trans chromosomal regulation of mammalian immune development
Source: PLoS Genet. 2018 Jun 8;14(6):e1007431. doi: 10.1371/journal.pgen.1007431 (PMC6010296; doi:10.1371/journal.pgen.1007431)
Supplement: S2 Table — (PDF) [file pgen.1007431.s005.pdf]

Supplemental Table 2: Post-blacklisting transchromosomal interactions unique to human B cells							
Chromosome	Start	End	Chromosome	Start	End	Gene associated with first anchor	Gene associated with second anchor
chr13	114300127	114349859	chr4	190100265	190173349	UPF3A,CHAMP1,LINC01054	
chr15	20349834	20399498	chr2	13400203	13449937	NA	
chr16	33600042	33649866	chr15	20199888	20250373		
chr20	29299930	29350304	chr16	34099510	34149997		
chr20	30811919	30850095	chr3	75649945	75700347		LINC00960,FRG2C
chr20	28600339	28650513	chr9	63849030	63899980	FRG1CP	FRG1JP
chr20	29050337	29100005	chr9	63799579	63849033	FRG1DP	FRG1JP,MIR4477B
chr20	29600069	29650090	chr9	61700037	61732933		
chr20	29849782	29899158	chr9	63799579	63849033		FRG1JP,MIR4477B
chr21	13999919	14049708	chr18	14097774	14150045		ZNF519
chr21	9749959	9800076	chr20	29050337	29100005	LINC01667	FRG1DP
chrY	56823168	56850027	chr21	7250367	7299248		
chrY	56823168	56850027	chr21	7951126	7999704		
chrY	56823168	56850027	chr21	8999951	9050237		LOC105372731
chrY	56850024	56900380	chr21	7951126	7999704		
chrY	56749632	56823171	chr22	18200307	18238906		LOC100996415
chrY	56823168	56850027	chr3	75649945	75700347		LINC00960,FRG2C
chrY	56850024	56900380	chr7	152249727	152300220		KMT2C

Supplemental Table 2: Post-blacklisting transchromosomal interactions unique to human CD4+ T cells

Chromosome	Start	End	Chromosome	Start	End	Gene associated with first anchor	Gene associated with second anchor
chr10	38598564	38649852	chr2	89754345	89800402		
chr16	70999961	71050384	chr1	146650529	146699783	HYDIN	NA,TRN-GTT7-1,TRN-GTT11-2,HYDIN2
chr18	49982	100133	chr4	190100265	190173349		
chr18	49982	100133	chr9	138100599	138149875		CACNA1B
chr19	57550126	57599953	chr12	133100006	133149598	ZNF416,ZNF550,ZIK1,ZNF530	ZNF10,ZNF140,ZNF891
chr19	57650177	57700161	chr12	132999908	133050006	ZNF154,ZNF551,ZSCAN4	ZNF26,ZNF84,LOC101928597
chr19	57700158	57749489	chr12	133149595	133199963	ZNF154,ZNF671,ZNF776	ZNF10,ZNF268
chr19	58049944	58100067	chr12	133149595	133199963	ZNF135,ZSCAN18,ZSCAN1	ZNF10,ZNF268
chr19	58150249	58200159	chr12	133050003	133100009	ZNF274,ZNF329	ZNF84,ZNF140
chr19	58150249	58200159	chr12	133100006	133149598	ZNF274,ZNF329	ZNF10,ZNF140,ZNF891
chr19	58200156	58249738	chr12	133050003	133100009	ZNF274,ZNF544	ZNF84,ZNF140
chr19	58249735	58299565	chr12	133199960	133250080	ZNF8,ZNF544	ZNF268,ANHX
chr19	58550132	58599999	chr12	133199960	133250080	MZF1,UBE2M,TRIM28,CHMP2A,CENPBD1P1,MZF1-AS1,MIR6807	ZNF268,ANHX
chr21	9549978	9599720	chr17	21649835	21700393		KCNJ18
chr7	76900021	76950000	chr1	83450098	83499886		NA

Supplemental Table 2: Post-blacklisting transchromosomal interactions unique to human CD8+ T cells							
Chromosome	Start	End	Chromosome	Start	End	Gene associated with first anchor	Gene associated with second anchor
chr11	38749473	38799904	chr8	51799829	51850052		PCMTD1,PXDNL
chr19	58500129	58550135	chr16	49876	100016	TRIM28,SLC27A5,ZBTB45	MPG,NPRL3,POLR3K,RHBDF1,SNRNP25,NA
chr21	46450088	46499859	chr13	114300127	114349859	DIP2A,DIP2A-IT1	UPF3A,CHAMP1,LINC01054
chr21	9749959	9800076	chr20	30811919	30850095	LINC01667	
chr7	76800089	76850042	chr1	83350033	83400125		NA

Supplemental Table 2: Post-blacklisting transchromosomal interactions common to human cells							
Chromosome	Start	End	Chromosome	Start	End	Gene associated with first anchor	Gene associated with second anchor
chr10	38250149	38299893	chr1	242300215	242350177		
chr10	38299890	38350019	chr1	242249759	242300218		
chr12	133050003	133100009	chr4	450109	499872		
chr12	133100006	133149598	chr4	350263	399918		
chr12	133100006	133149598	chr4	450109	499872		
chr12	133149595	133199963	chr4	99992	150244		
chr12	133149595	133199963	chr4	350263	399918		
chr12	133199960	133250080	chr4	150241	199975		
chr16	33600042	33649866	chr6	300053	350084	Slc12a8	
chr16	33600042	33649866	chr6	350081	399537	Slc12a8	
chr16	33649863	33699800	chr6	300053	350084	Heg1,Slc12a8	
chr16	33699797	33750323	chr6	300053	350084	Heg1	
chr17	21795739	21849776	chr7	63000284	63050047	Zfp820	
chr17	21795739	21849776	chr7	68149862	68200218	Zfp820	Igf1r
chr17	62450045	62500009	chr7	128500198	128549626		Bag3
chr18	80249971	80261931	chr4	190100265	190173349	Pqlc1	
chr18	15299582	15350150	chr9	65048966	65078822	E430002N23Rik	Dpp8
chr18	15350147	15398346	chr9	65048966	65078822	Aqp4	Dpp8
chr20	29350301	29399864	chr3	75649945	75700347		Platr10
chr20	29399861	29450169	chr3	75649945	75700347		Platr10
chr20	64299662	64334167	chr6	150175	199780		
chr20	28600339	28650513	chr9	40950212	41000187		Crtam,Jhy
chr20	30811919	30850095	chr9	63799579	63849033		
chr21	46599801	46650009	chr13	114300127	114349859		Ndufs4
chr21	13400435	13449816	chr18	14700077	14749818		Psma8
chr21	13449813	13499787	chr18	14650050	14700080		Ss18
chr21	5216622	5249633	chr20	28999722	29050340		
chr21	5216622	5249633	chr20	29050337	29100005		
chr21	5216622	5249633	chr20	29499581	29550142		

chr21	5216622	5249633	chr20	29849782	29899158		
chr21	5216622	5249633	chr20	30349871	30400222		
chr22	16400393	16449805	chr2	89754345	89800402		Olfr1254
chr22	32149887	32200280	chr9	98700128	98749677		E330023G01Rik
chr3	49649844	49700638	chr1	16599855	16649766		Ube2w,Eloc
chr3	75649945	75700347	chr1	143199926	143250600	Platr10	
chr7	62300512	62349945	chr1	143199926	143250600	Ndn	
chrY	56699637	56749635	chr1	143199926	143250600		
chrY	56749632	56823171	chr1	143199926	143250600		
chrY	56823168	56850027	chr1	143199926	143250600		
chrY	56850024	56900380	chr10	125850030	125900082		
chrY	56850024	56900380	chr10	125900079	125949982		
chrY	56823168	56850027	chr16	33699797	33750323		Heg1
chrY	56823168	56850027	chr16	34049728	34099513		Kalrn
chrY	56823168	56850027	chr18	100130	149971		
chrY	56823168	56850027	chr2	88750494	88799987		Olfr1200,Olfr1199,Olfr1201
chrY	56823168	56850027	chr2	95899859	95949911		
chrY	56823168	56850027	chr2	95949908	96000075		
chrY	56823168	56850027	chr2	97149974	97199975		
chrY	56850024	56900380	chr2	95899859	95949911		
chrY	11100271	11149871	chr21	8999951	9050237		
chrY	56823168	56850027	chr21	9749959	9800076		
chrY	56850024	56900380	chr21	5216622	5249633		
chrY	56850024	56900380	chr21	9749959	9800076		
chrY	56699637	56749635	chr22	18709576	18750410		
chrY	56699637	56749635	chr22	18850026	18899542		
chrY	56749632	56823171	chr22	18709576	18750410		
chrY	56749632	56823171	chr22	18850026	18899542		
chrY	56850024	56900380	chr7	152400017	152450104		
chr12	132999908	133050006	chr4	299942	350266		
chr12	132999908	133050006	chr4	399915	450112		

chr12	133199960	133250080	chr4	199972	249570		
chr18	14400332	14449709	chr13	18599942	18650338		
chr18	80199883	80249974	chr4	190100265	190173349	Txnl4a,Hsbp1l1,Rbfa	
chr20	199977	250026	chr1	257716	297872		
chr20	250023	299742	chr1	257716	297872		
chr21	5216622	5249633	chr20	29100002	29150103		
chr21	32399927	32450047	chr3	75400266	75449982		Wdr49
chrY	56850024	56900380	chr1	143199926	143250600		
chrY	56850024	56900380	chr7	152350028	152400020		
chr12	132999908	133050006	chr4	350263	399918		
chr12	133050003	133100009	chr4	350263	399918		
chr12	133100006	133149598	chr4	150241	199975		
chr12	133100006	133149598	chr4	299942	350266		
chr16	33649863	33699800	chr6	350081	399537	Heg1,Slc12a8	
chr21	13499784	13549777	chr18	14600085	14650053		Ss18
chrY	56823168	56850027	chr10	125900079	125949982		
chrY	56850024	56900380	chr16	33699797	33750323		Heg1
chrY	56823168	56850027	chr7	152400017	152450104		

Supplemental Table 2: Post-blacklisting transchromosomal interactions unique to mouse B cells							
Chromosome	Start	End	Chromosome	Start	End	Gene associated with first anchor	Gene associated with second anchor
chr18	3050234	3099928	chr16	3250182	3300027		
chr2	3150025	3200385	chr1	88249817	88299855	Fam171a1	6430706D22Rik,Hjurp,Mroh2a
chrY	90699748	90750101	chrX	169949960	169999781	NA	Mid1,G530011O06Rik

Supplemental Table 2: Post-blacklisting transchromosomal interactions unique to mouse CD4+ T cells

Chromosome	Start	End	Chromosome	Start	End	Gene associated with first anchor	Gene associated with second anchor
chr16	17199671	17250186	chr9	123449722	123500196	Ube2l3,Hic2,Rimbp3	Limd1,Lars2
chr17	23400235	23450582	chr1	88249817	88299855	Vmn2r116	6430706D22Rik,Hjurp,Mroh2a
chr18	3150071	3200056	chr16	3250182	3300027		
chr19	6650028	6687996	chr11	22999972	23049848		Cct4,Fam161a

Supplemental Table 2: Post-blacklisting transchromosomal interactions unique to mouse CD8+ T cells							
Chromosome	Start	End	Chromosome	Start	End	Gene associated with first anchor	Gene associated with second anchor
chr12	20200108	20249248	chr4	145650000	145699976	NA	Zfp980
chr12	20200108	20249248	chr4	146900733	146950126	NA	Gm13149,NA
chr16	3100168	3149912	chr13	9000057	9050074		
chr19	13849927	13901168	chr16	3250182	3300027	Olfr1504,Olfr1502	
chr19	49050043	49100267	chr16	3250182	3300027		
chr3	14650284	14700461	chr1	88249817	88299855	Car13	6430706D22Rik,Hjurp,Mroh2a
chr6	58600107	58650375	chr4	147099542	147150197	Abcg2	Zfp991
chr8	3099911	3150195	chr4	3249850	3299716		NA

Supplemental Table 2: Post-blacklisting transchromosomal interactions common to mouse cells

Chromosome	Start	End	Chromosome	Start	End	Gene associated with first anchor	Gene associated with second anchor
chr12	51450299	51499933	chr1	88249817	88299855		6430706D22Rik,Hjurp,Mroh2a
chr12	20200108	20249248	chr4	146149438	146200078	NA	Zfp600
chr12	20200108	20249248	chr4	146450134	146499188	NA	Zfp992
chr12	20200108	20249248	chr4	146499185	146549996	NA	1700095A21Rik,Zfp981
chr12	20200108	20249248	chr4	146708801	146750182	NA	NA,Gm20875
chr12	20200108	20249248	chr4	147299595	147350006	NA	
chr12	20200108	20249248	chr4	147400251	147449702	NA	
chr12	20200108	20249248	chr4	147750002	147800228	NA	Zfp984
chr12	3000001	3049529	chr5	3000001	3050004		V1rg10
chr13	3000480	3050284	chr4	3199973	3249853		
chr13	3000480	3050284	chr6	3149518	3199755		Rn18s
chr15	3050001	3100097	chr4	3199973	3249853		
chr15	3050001	3100097	chr4	3249850	3299716		NA
chr15	3149859	3199885	chr4	3199973	3249853		
chr16	3100168	3149912	chr4	3050773	3099791		
chr16	3250182	3300027	chr5	79399974	79449996		
chr17	3000418	3050005	chr1	88249817	88299855		6430706D22Rik,Hjurp,Mroh2a
chr17	23300132	23349848	chr1	88249817	88299855	Vmn2r115,Vmn2r114	6430706D22Rik,Hjurp,Mroh2a
chr17	23499743	23549799	chr1	88249817	88299855		6430706D22Rik,Hjurp,Mroh2a
chr17	3000418	3050005	chr4	3050773	3099791		
chr19	3150425	3200160	chr16	3250182	3300027	1700030N03Rik	
chr3	3000788	3050004	chr1	88249817	88299855		6430706D22Rik,Hjurp,Mroh2a
chr3	3099954	3149828	chr1	88249817	88299855		6430706D22Rik,Hjurp,Mroh2a
chr3	3200068	3249959	chr1	88249817	88299855		6430706D22Rik,Hjurp,Mroh2a
chr4	3050773	3099791	chr1	88249817	88299855		6430706D22Rik,Hjurp,Mroh2a
chr5	77400011	77449890	chr4	3199973	3249853	Igfbp7	
chr6	3050001	3099781	chr4	3050773	3099791		
chr6	3149518	3199755	chr4	3199973	3249853	Rn18s	
chr6	3399884	3449758	chr4	3050773	3099791		

chr6	3449755	3499596	chr4	3050773	3099791	Vps50,Hepacam2	
chr6	58550148	58600110	chr4	147400251	147449702	Abcg2	
chr6	58600107	58650375	chr4	146450134	146499188	Abcg2	Zfp992
chr6	58600107	58650375	chr4	147400251	147449702	Abcg2	
chr6	67649862	67699998	chr4	146450134	146499188		Zfp992
chr6	67649862	67699998	chr4	146499185	146549996		1700095A21Rik,Zfp981
chr6	67649862	67699998	chr4	147400251	147449702		
chr7	3000001	3049771	chr4	3199973	3249853		
chr7	3049768	3100089	chr4	3199973	3249853		
chr9	3300202	3349883	chr1	88249817	88299855	Alkbh8	6430706D22Rik,Hjurp,Mroh2a
chrX	124100008	124149871	chr4	156306042	156349983	Vmn2r121	Vmn2r-ps159,Vmn2r125
chr15	3100094	3149862	chr4	3199973	3249853		
chr16	3181571	3250185	chr4	3050773	3099791		
chr16	3181571	3250185	chr5	79399974	79449996		
chr19	3098793	3150428	chr16	3250182	3300027		
chr3	3050001	3099957	chr1	88249817	88299855		6430706D22Rik,Hjurp,Mroh2a
chr6	3349828	3399887	chr4	3050773	3099791	Samd9l	
