# Supplementary material for: Genome-wide analysis reveals no evidence of trans chromosomal regulation of mammalian immune development
Source: PLoS Genet. 2018 Jun 8;14(6):e1007431. doi: 10.1371/journal.pgen.1007431 (PMC6010296; doi:10.1371/journal.pgen.1007431)
Supplement: S3 Table — (PDF) [file pgen.1007431.s006.pdf]

Supplemental Table 3: Antibodies used in study

Target	Species	Clone	Source
TCRβ	Mouse	H57-597	BD
CD19	Mouse	ID3	BD
B220	Mouse	RA3-6B2	BD
IgM	Mouse	R6-60.2	BD
IgD	Mouse	IA6-2	BD
CD4	Mouse	GK1.5-7	In house
CD8	Mouse	53-6.7	eBioscience
CD62L	Mouse	MEL-14	In house
CD44	Mouse	IM7	Biolegend
Ly6G	Mouse	1A8	BD
Ly6C	Mouse	AL-21	BD
Flt3R	Mouse	A2F10	BD
CD138	Mouse	281-2	Biolegend
CD22	Mouse	12a/CD22	BD
αβ TCR	Human	IP26	eBioscience
CD4	Human	RPA-T4	BD
CD45RA	Human	5H9	BD
CD25	Human	M-A251	BD
CD14	Human	63D3	Biolegend
CD16	Human	3G8	BD
HLA-DR	Human	L243	eBioscience
CD19	Human	HIB19	Biolegend
