# Supplementary material for: Genome-wide analysis reveals no evidence of trans chromosomal regulation of mammalian immune development
Source: PLoS Genet. 2018 Jun 8;14(6):e1007431. doi: 10.1371/journal.pgen.1007431 (PMC6010296; doi:10.1371/journal.pgen.1007431)
Supplement: S4 Table — (PDF) [file pgen.1007431.s007.pdf]

Supplemental Table 4: Details of in situ HiC libraries

		valid pairs			same id				chimeras					Threshold used in HiC library processing			
**Sample**	**Processed library**	**Total**	**% mapped**		**dangling**	**% dangling**	**self.circle**	**% self.circle**	**total**	**mapped**	**multi**	**invalid**	**% invalid**	**min.inward**	**min.outward**	**max.frag**	**chim.dist**
CD4+ T cells 1 (mm)	CD4T1	166436549	74.14%	2.20%	1147179	0.69%	53663	0.03%	59662349	40885028	27289115	714070	1.20%	1000	25000	1400	1200
CD4+ T cells 2 (mm)	CD4T2	224467767	74.22%	2.36%	1109860	0.49%	66588	0.03%	85077998	58329520	39292730	1056225	1.24%	1000	25000	1400	1200
Mature B cells 1 (mm)	MATB2	159979424	74.88%	1.82%	1134844	0.71%	59916	0.04%	56190704	38793970	26084780	810259	1.44%	1000	25000	1400	1200
Mature B cells 2 (mm)	MATB1	374899629	74.63%	1.99%	2944324	0.79%	118348	0.03%	122431301	83875739	56029371	1769202	1.45%	1000	25000	1400	1200
CD8+ T cells 1 (mm)	CD8T1	200460280	75.29%	2.29%	1126203	0.56%	58932	0.03%	63677747	43943867	29420917	978149	1.54%	1000	25000	1400	1200
CD8+ T cells 2 (mm)	CD8T2	258152814	74.47%	2.39%	1319170	0.51%	73529	0.03%	82604796	56489591	37777038	1426997	1.73%	1000	25000	1400	1200
Mature B cells 1 (hg)	HB1	280648411	74.28%	2.47%	1305535	0.47%	51989	0.02%	87686106	57985298	35084117	1584402	1.81%	1000	4000	1200	1200
Mature B cells 2 (hg)	HB2	280520979	74.24%	2.35%	1389123	0.50%	45494	0.02%	94323123	62301914	37747552	1669207	1.77%	1000	4000	1200	1200
CD4+ T cells 1 (hg)	H4T1	225299757	73.98%	2.31%	943751	0.42%	46140	0.02%	70432552	45514570	26588471	1350506	1.92%	1000	4000	1200	1200
CD4+ T cells 2 (hg)	H4T2	258000998	74.32%	2.27%	1033899	0.40%	54339	0.02%	80667133	52301854	30540956	1678493	2.08%	1000	4000	1200	1200
CD8+ T cells 1 (hg)	H8T1	246951691	73.10%	2.74%	1148054	0.46%	51026	0.02%	83692260	54491187	32790547	1592679	1.90%	1000	4000	1200	1200
CD8+ T cells 2 (hg)	H8T2	224469689	74.51%	1.58%	954666	0.43%	41327	0.02%	72382443	47502613	27975070	1382217	1.91%	1000	4000	1200	1200

Details of PCHiC libraries processed

		valid pairs			same id				chimeras					Threshold used in HiC library processing			
**Sample**	**Processed library**	**Total**	**% mapped**	**% marked**	**dangling**	**% dangling**	**self.circle**	**% self.circle**	**total**	**mapped**	**multi**	**invalid**	**% invalid**	**min.inward**	**min.outward**	**max.frag**	**chim.dist**
CD4+ T cells 1 (hg)	nCD4 rep1	755775673	71.51%	12.20%	27548008	3.64%	6909442	0.91%	153979538	93282802	42774015	2090230	1.36%	1000	300000	800	800
CD4+ T cells 2 (hg)	nCD4 rep2	640489393	70.14%	15.51%	5607313	0.88%	5948503	0.93%	125494703	75191685	34623071	1410957	1.12%	1000	300000	800	800
CD4+ T cells 3 (hg)	nCD4 rep3	694136827	74.48%	8.89%	21408749	3.08%	4427383	0.64%	144044708	91621760	41879304	1426829	0.99%	1000	300000	800	800
CD4+ T cells 4 (hg)	nCD4 rep4	707459718	75.30%	8.97%	3193016	0.45%	4048681	0.57%	137147130	89024448	40431083	1502832	1.10%	1000	300000	800	800
